# Thermochemical Studies of Small Carbohydrates

**DOI:** 10.1021/acs.joc.3c02465

**Published:** 2024-01-16

**Authors:** Kathleen M. Morgan, Joshua H. Baraban

**Affiliations:** † Department of Chemistry, Xavier University of Louisiana, 1 Drexel Drive, New Orleans, Louisiana 70125, United States; ‡ Department of Chemistry, Ben-Gurion University of the Negev, Beer Sheva 841051, Israel

## Abstract

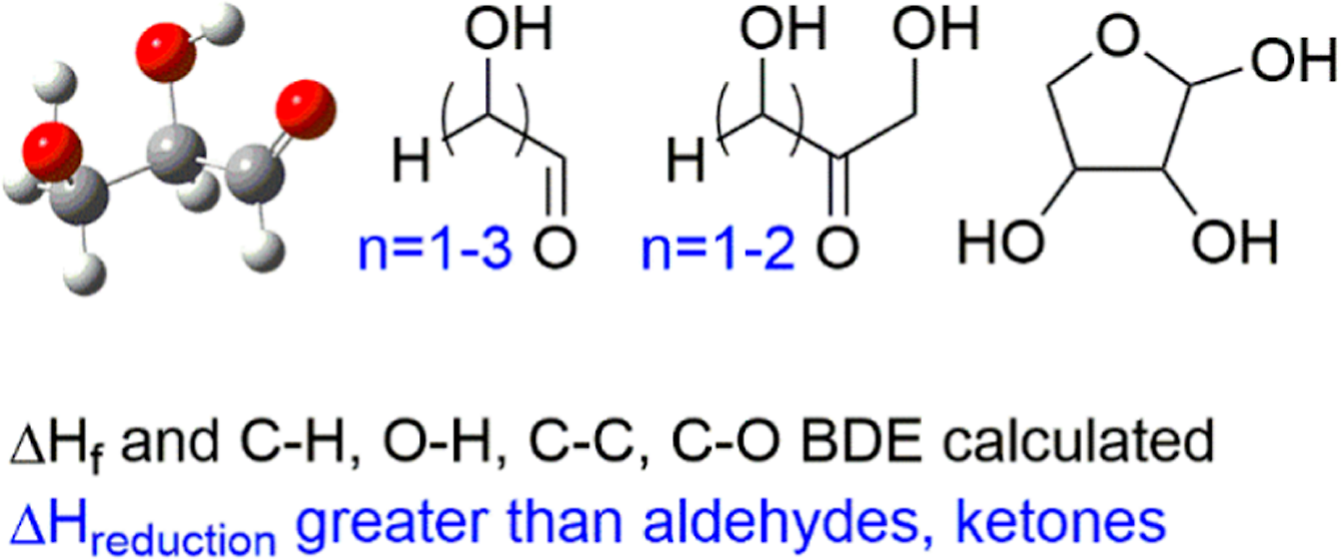

Despite their prevalence in biomass and importance in biochemistry, there is still much to be learned about simple carbohydrates. Gas-phase calculations are reported here on two trioses and three tetroses. For aldotetroses, both the open-chain and furanose forms are considered. Enthalpies of reduction to polyols are calculated at the CBS-APNO level of theory, and comparisons to simple aldehydes and ketones are made. Heats of formation are calculated in two ways with overall good agreement. The heat of formation of glyceraldehyde obtained from modified HEAT calculations is also reported. Finally, calculated bond energies are presented, and the influence of the structure on the bond energies is discussed.

## Introduction

Carbohydrates are among the most abundant molecules in the world. Degradation of biomass carbohydrates as a renewable fuel or feedstock for commodity chemicals has received much interest and development in the past decade.^1^ Carbohydrates play a major role in biochemistry.^2^ In addition, glycolaldehyde^3^ and dihydroxyacetone^4^ have been observed in interstellar space, with potential relevance to the origins of life on earth. However, there are many fundamental properties of simple carbohydrates that remain unknown. In this report, the results of a computational study of the gas-phase heats of reduction, heats of formation, and bond energies for carbohydrates with two, three, and four carbons are described.

**Figure 1:**
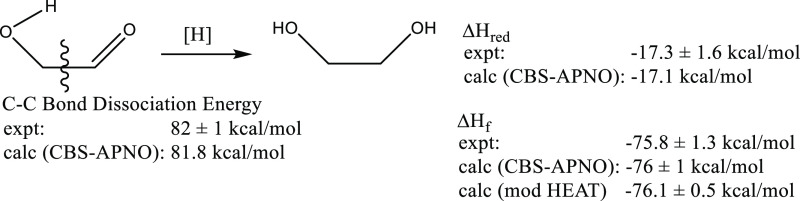
Heat of reduction, heat of formation, and C–C bond energy in glycolaldehyde.^5^

In a previous publication, we reported the gas-phase heat of reduction (Δ*H*_red_) and heat of formation (Δ*H*_f_) for glycolaldehyde, obtained via experiment and calculations.^5^ As shown in Figure 1, the methods are in excellent agreement. The Δ*H*_f_ determined by experiment is consistent with that obtained using rigorous modified HEAT calculations. The CBS-APNO-calculated Δ*H*_f_ is also in agreement and was obtained by combining the CBS-APNO-calculated Δ*H*_red_ with the experimentally determined Δ*H*_f_ for ethylene glycol from the literature. Experiment and theory were also the same for the carbon–carbon bond dissociation energy, the first reported carbon–carbon bond energy in a carbohydrate.

There are limited condensed-phase experimental thermochemical data for trioses glyceraldehyde and dihydroxyacetone and the three tetroses erythrose, threose, and erythrulose. The heat of combustion of solid glyceraldehyde has been reported as −336.9, −346.1, and −348.9 kcal/mol.^6^ The variation in these values may be because glyceraldehyde is difficult to purify and crystallize, and it is also possible that the experiments were carried out on different crystalline forms of the sugar. The literature values for the heat of combustion of liquid glyceraldehyde have an even greater range: −338.1, −359.44, and −423.02 kcal/mol.^6^ There is only one reported heat of combustion for solid dihydroxyacetone, −343.22 kcal/mol,^6^ and no heats of combustion or formation have been reported for any of the three tetroses. Given the limitations of these data as well as the low vapor pressures of the trioses and tetroses, gas-phase thermochemical data are very difficult to obtain. Note that the Δ*H*_f_ is available for several solid pentoses and hexoses, with some measurements obtained for the open-chain forms while others were obtained on cyclic forms.^6^ The behavior of carbohydrates in aqueous solution has received significantly more study.^7^ Such studies are clearly relevant to biochemical reactions, but an extension of these results to the gas phase is not straightforward due to strong solvent–solute interactions and the various dimers and oligomers that carbohydrates form in solution.

In this study, the CBS-APNO calculations in the gas phase as carried out previously for glycolaldehyde are extended to glyceraldehyde and dihydroxyacetone, as well as modified HEAT calculations for glyceraldehyde. Some of the CBS-APNO calculations are also reported for the three isomeric tetroses, including both the acyclic and furanose forms of the aldotetroses. Comparisons are made among isomeric compounds, homologous compounds, and simple aldehydes and ketones. Due to experimental limitations, it was not possible to measure the Δ*H*_red_ of the trioses and tetroses. Given the excellent agreement between experiment and theory for glycolaldehyde, as noted above, there is good reason to expect that the computational data reported here will provide meaningful insights into the gas-phase carbohydrates of interest.

Bond energies are all but unknown for carbohydrates, with the first experimentally determined value being for the carbon–carbon bond from our prior study of glycolaldehyde.^5^ Knowledge of bond energies is helpful in understanding decomposition pathways, such as degradation of biomass or metabolism of sugars. As such, the remaining bond energies in glycolaldehyde as well as all bond energies in glyceraldehyde and dihydroxyacetone are reported at the CBS-APNO level of theory. CBS-QB3 calculations of the tetrose bond energies are also provided and, while less accurate, allow qualitative discussion.

## Results and Discussion

The majority of thermochemical data in the literature has been reported for relatively simple monofunctional compounds. In this report, structures, gas-phase heats of reduction (Δ*H*_red_), heats of formation (Δ*H*_f_), and bond energies of carbohydrates having two to four carbons are obtained using computational methods linked to experiments. They are also compared to simple aldehydes and ketones as well as to each other. In this study, CBS-APNO calculations are used; Wiberg’s benchmarking study of similar reductions found the best agreement with experiment using G4, CBS-APNO, and W1BD methods.^8^ In addition, previous comparisons to experiment for heats of reduction^5,8,9^ and bond energies^5^ generally agree within experimental error.

### Structures

**Figure 2:**
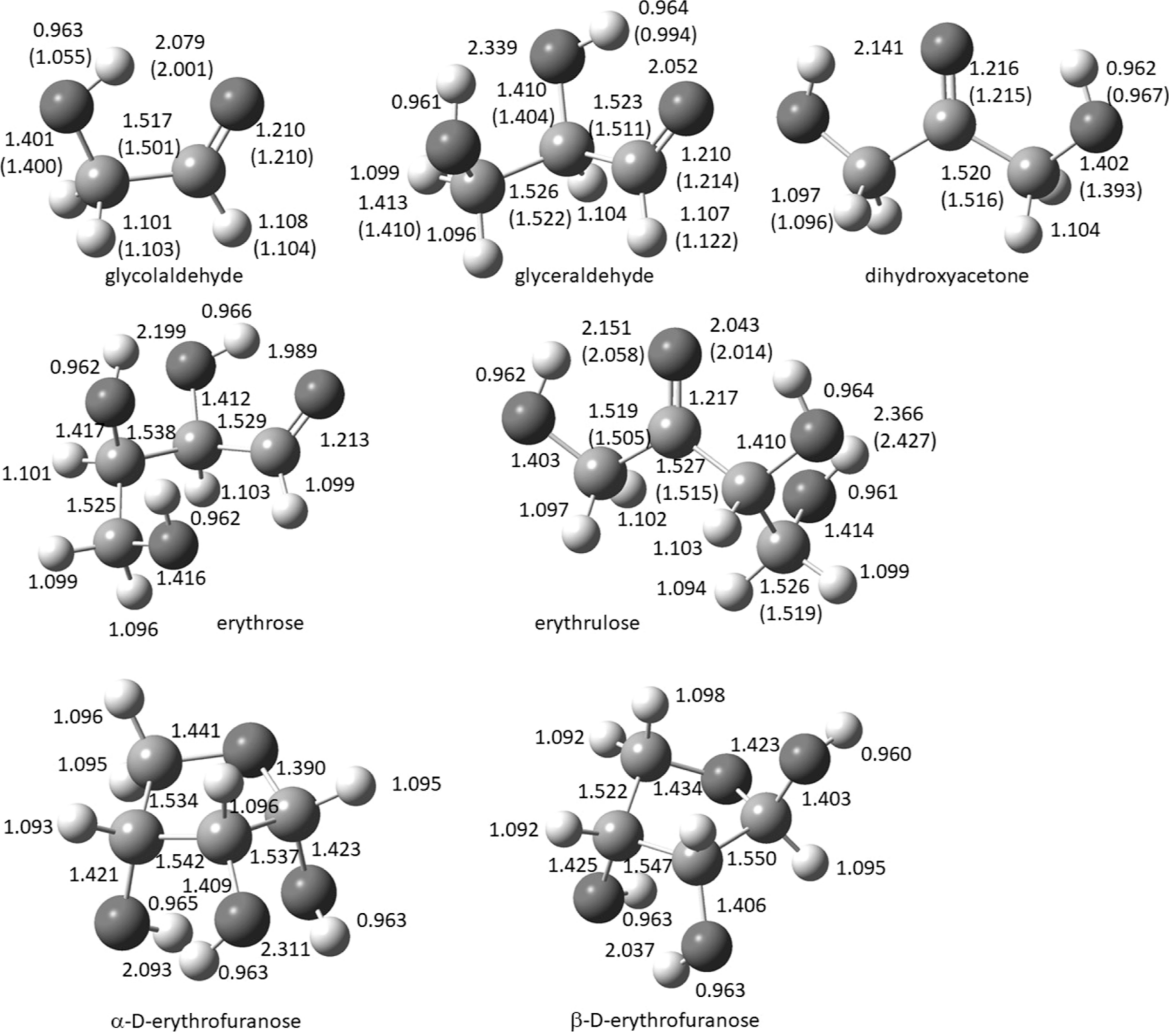
Calculated (experimental) structures of select carbohydrates.

Although the carbohydrates with two to four carbons have received prior computational attention,^5,10^ in this study, they are all re-evaluated using the CBS-APNO method [QCISD/6-311G(d,p) optimization] with similar results. Carbohydrates in the gas phase exist in multiple conformations, and the global minimum structures obtained for several of the compounds in the gas phase are shown in Figure 2, together with calculated bond lengths and, if available, experimental data. The minimum energy structures all exhibit the maximum number of hydrogen bonds. Structures for threose and its two furanose forms, for higher energy conformations of compounds in Figure 2, and for the lowest two conformations of glyceraldehyde optimized at the AE-CCSD(T)/cc-pVQZ level of theory for the modified HEAT calculations are available in the Supporting Information.

The calculated structure for glycolaldehyde agrees reasonably well with the experimental values determined by microwave spectroscopy, except for the O–H bond, but this discrepancy has been addressed previously and supports the calculated value.^5^ Previous computational study of glycolaldehyde revealed two additional conformations within 3.6 kcal/mol of the global minimum,^5^ but only the lowest-energy conformation has been observed experimentally.^11^ In the case of glyceraldehyde, one major and one minor conformation has been observed experimentally by gas-phase electron diffraction (*r*_e_ structure), and three other low-energy conformers are predicted computationally.^12^ Dihydroxyacetone was studied using a combination of electron diffraction, microwave experiments, G3X, and other calculations; nine conformations were reported with three of these being low-energy conformations.^10a^ The rotational spectrum of erythrulose, the ketotetrose, was also obtained in the gas phase, and only one open-chain conformation was observed.^10c^

The aldotetroses can adopt chain or furanose ring structures, and both were considered. Alonso described a gas-phase study of erythrose, where the sugar was vaporized using a laser ablation technique, allowing the rotational spectrum to be obtained using chirped pulse Fourier transform microwave spectroscopy.^10b^ This study showed that the furanose form is dominant in the gas phase with one α- and one β-structure identified with the support of calculations.

**Figure 3:**
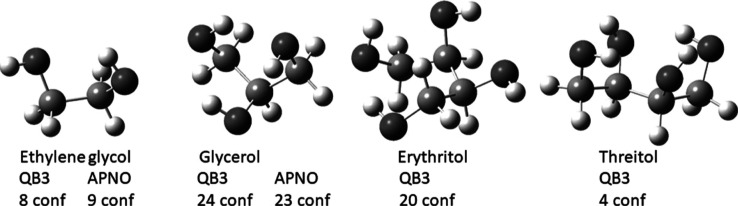
Lowest-energy conformations of polyols, with the number of conformations within 3 kcal/mol.

The reduced forms of the carbohydrates, the polyols, are relevant to the heat of reduction analysis and were also calculated. They have even greater conformational diversity. Cramer and Truhlar’s computational study of ethylene glycol describes 10 unique conformations.^13^ Hadad’s study of glycerol^14^ revealed 126 unique conformations, with the 26 lowest-energy conformations found to be within approximately 2.5 kcal/mol of the global minimum. In both studies, the computational method used influenced the relative stability of the conformations, with the more robust methods giving reasonably consistent results. Rosado studied erythritol and threitol, the meso and chiral diastereomers of the tetrol, using density functional theory, focusing on the lower-energy conformers.^15^ In the current study, the various low-energy conformations of ethylene glycol and glycerol were recalculated using CBS-QB3 and CBS-APNO methodology. In addition, the low-lying conformations of erythritol and threitol were calculated using the CBS-QB3 method. The global minimum of each tetrol was also recalculated at the CBS-APNO level of theory. The structures of the polyols in their lowest-energy conformations are shown in Figure 3. Also reported here is the number of conformations within 3 kcal/mol.

### Comparisons of Isomers

**Figure 4:**
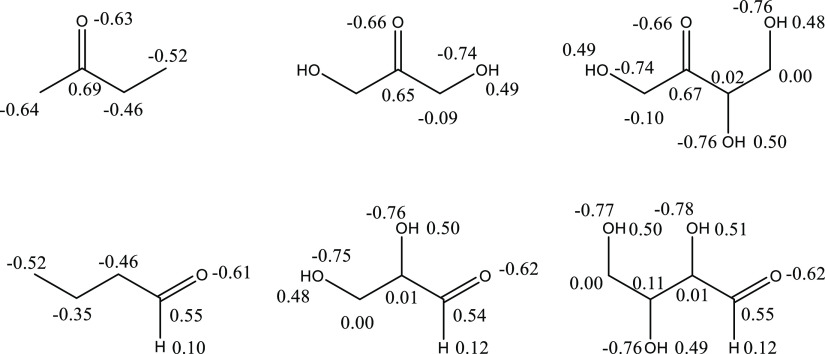
NPA charges from CBS-APNO calculations.

Simple ketones have generally lower energy than isomeric aldehydes, a result of the stabilizing effect of alkyl groups on the electron-poor carbonyl carbon.^16^ As examples, the calculated difference in enthalpy between acetone and propanal is 7.3 kcal/mol, and 7.5 kcal/mol between 2-butanone and butanal. The difference in enthalpy between aldoses and ketoses is significantly less. Glyceraldehyde is calculated to be 1.9 kcal/mol less stable than dihydroxyacetone, and erythrose is 2.9 kcal/mol less stable than erythrulose. Figure 4 shows the natural population analysis (NPA) atomic charges^17^ obtained for the global minimum structures during the CBS-APNO calculations.

The carbonyl carbons in ketones and ketoses are significantly more positive than those in aldehydes and aldoses, while the carbonyl oxygen charge is about the same in all molecules. In carbohydrates, the electron-withdrawing hydroxyl groups deplete the electron density on the α-carbons to near zero, decreasing their ability to stabilize the carbonyl carbons. This effect is greater in ketoses, where there are two such interactions. For example, the NPA charges on each α-carbon in butanone and butanal decrease by 0.5 electrons compared to the corresponding tetroses.

**Figure 5:**
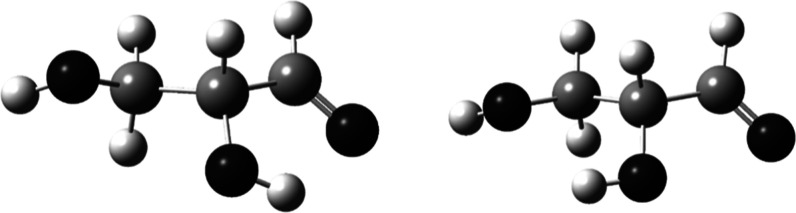
Glyceraldehyde with C2 OH hydrogen bonds with C1 (left) and C3 O (right).

An additional difference between the ketoses and aldoses is that the global minimum structure for the ketoses has two hydrogen bonds to the carbonyl oxygen, while aldoses have only one. Figure 5 shows the optimized structures of two conformations of glyceraldehyde, one having the C2 OH hydrogen bonded to the carbonyl oxygen and the other with the C2 OH hydrogen bonded to the C3 oxygen. The other parts of the structures are essentially the same, though with some differences arising from the optimization. The structure having the hydrogen bond to the carbonyl oxygen is more stable by 1.9 kcal/mol.

With regard to the aldotetroses, the furanose forms are more stable than the linear forms. This is consistent with simple aldehydes forming hemiacetals with favorable enthalpy.^18^ α-Erythrofuranose is 4.7 kcal/mol more stable than the chain form of erythrose and 1.5 kcal/mol more stable than the β-anomer. β-Threofuranose is 1.9 kcal/mol more stable than the chain form of threose and 0.8 kcal/mol more stable than the α-anomer.

### Heats of Reduction

One of the most fundamental reactions of a carbonyl group is the reduction to the corresponding alcohol. This reduction enthalpy (Δ*H*_red_) for the six smallest carbohydrates is included in Table 1, together with values for simple aldehyde and ketone reductions.^19^ The CBS-APNO-calculated Δ*H*_red_ is within 0.5 kcal/mol of the experiment, where available. The Δ*H*_red_ for the aldotetroses uses the chain form rather than the furanose form. Reduction of erythrulose can produce both meso erythritol and chiral threitol, and both are included in Table 1. Note that CBS-QB3 calculations find threitol to be more stable than erythritol by 2.9 kcal/mol. The four simple aldehydes averaged in Table 1 are acetaldehyde, propanal, butanal, and 2-methylpropanal, and the four simple ketones are acetone, 2-butanone, 3-methyl-2-butanone, and 2-pentanone. The tabulated CBS-QB3- and CBS-APNO-calculated Δ*H*_red_ uses the energy-weighted average of conformations. This becomes increasingly important as the size of the molecule and the number of low-energy conformations increase, as shown in Table S1. Due to the size and number of low-lying conformations of the tetrols, the CBS-QB3 minimum-energy structure was recalculated at CBS-APNO, and the enthalpy was corrected using the CBS-QB3 difference in enthalpy between the minimum and energy-weighted average.

**Table 1:** Gas-Phase Carbonyl Heats of Reduction (kcal/mol)^a^

compound	experiment	CBS-QB3	CBS-APNO
glycolaldehyde	–17.3 ± 1.6^5^	–16.0	–17.1
glyceraldehyde		–16.6	–17.8
dihydroxyacetone		–14.3	–15.9
erythulose to threitol		–17.0	–18.3
erythrulose to erythritol		–14.1	–15.5
erythrose		–17.1	–18.4
threose		–18.9	–20.1
propanal	–16.6 ± 0.2^19b^	–15.1	–16.3
acetone	–13.2 ± 0.2^19b^	–12.5	–13.5
average 4 aldehydes	–16.5 ± 0.2^19b^		
average 4 ketones	–12.7 ± 0.5^19b^		

a The calculated entries include the contributions of higher-energy conformations.

Reduction of carbohydrates is more exothermic than reduction of simple aldehydes and ketones and can be explained by stabilization of the reduction product combined with destabilization of the carbonyl compound. Carbohydrates and their reduced forms experience internal hydrogen bonding that is not present in the simple aldehydes, ketones, or alcohols. This hydrogen bonding can take place between the carbonyl and hydroxyl group in the carbohydrate or between two hydroxyl groups in larger carbohydrates and in the polyol reduction products. As noted above, the hydrogen bond from the OH to the carbonyl oxygen as found in carbohydrates is stronger than that between two alcohols, as found in the polyols. However, the polyols can form one more hydrogen bond than the carbohydrate can. For example, glyceraldehyde and dihydroxyacetone can form two hydrogen bonds, while glycerol can form three, thereby suggesting that the reduction product is stabilized. The electrostatic destabilization of carbohydrates has also been discussed above.

### Heats of Formation

While it is possible to calculate Δ*H*_f_ using rigorous computational methods, it is currently not routine to do so for molecules with six or more heavy atoms. In this study, the Δ*H*_f_ of the global minimum structure of glyceraldehyde and the next higher conformation were obtained by using fully ab initio modified HEAT calculations. In addition, gas-phase Δ*H*_f_ values for the carbohydrates having two to four carbons were obtained using two indirect methods that blend calculation with experimental data (Table 2). Here, reaction enthalpies are calculated, allowing greater cancellation of error due to imperfections in the computational model. The first method combines the carbonyl Δ*H*_red_ as obtained above with the gas-phase Δ*H*_f_ of the carbonyl reduction product, known from experiment. As noted above, this method was successfully applied for the reduction of glycolaldehyde to ethylene glycol. A limitation of this approach is that the Δ*H*_f_ of the reduction product must be known in the gas phase, and this is the case for glycerol^19b^ and erythritol^19b^ but not threitol. Heats of vaporization or sublimation can also be difficult to obtain for compounds of such a low volatility. The number of conformations of the polyols can increase rapidly with size, which makes the calculations nontrivial. As a result, this method of determining Δ*H*_f_ of the carbohydrates is not easily applied to molecules larger than tetroses.

**Table 2:** Calculated Gas-Phase Δ*H*_f_, kcal/mol

	mod HEAT	using Δ*H*_red_	using Scheme 1	literature
glycolaldehyde	–76.1 ± 0.5	–76.0	–76.1	–75.8 ± 1.3^5^ (expt.)
glyceraldehyde	–120.6 ± 0.5	–120.3	–122.0	
				
global minimum	–119.5 ± 0.5	–120.6	–122.3	
global min +1		–119.5	–121.1	
dihydroxyacetone		–122.2	–124.5	–125 ± 1^10a^ (calc)
erythulose		–169.8	–169.6	
erythrose, chain		–166.8	–165.8	
threose, chain			–166.9	
α-erythrofuranose			–170.8	
β-erythrofuranose			–169.3	
α-threofuranose			–168.3	
β-threofuranose			–169.2	

**Scheme 1:**
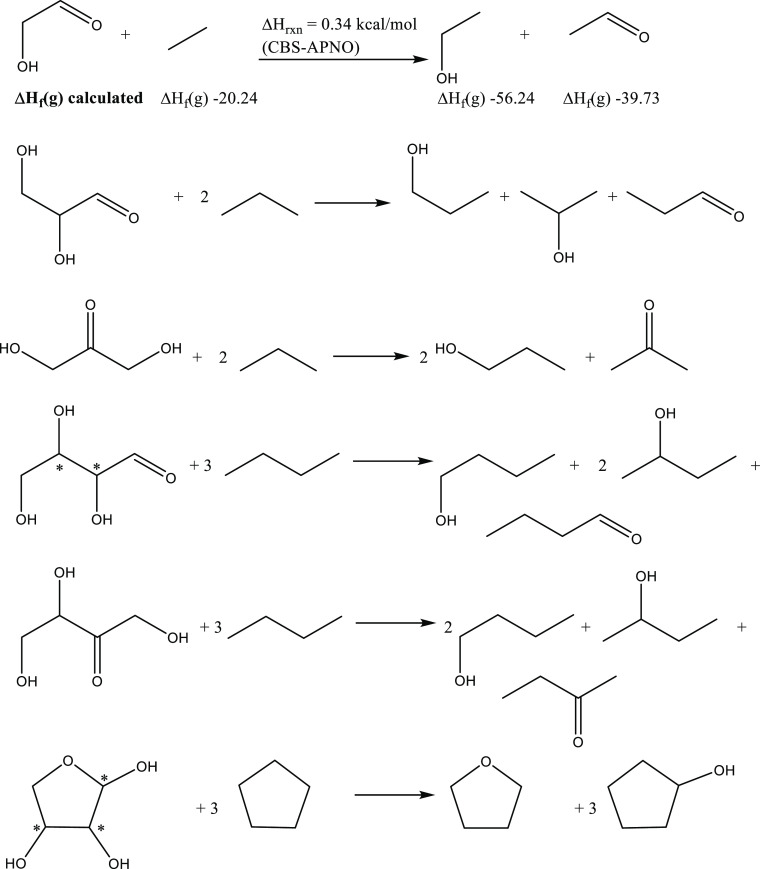
Isodesmic Reactions Used to Obtain Carbohydrate Δ*H*_f_

Another approach uses simple reference compounds in an isodesmic-type equation.^20^ Here, the number of each type of bond is the same on each side of the hypothetical chemical reaction. These equations are shown in Scheme 1. The heat of the reaction is obtained using CBS-APNO calculations. This result is then combined with well-established Δ*H*_f_ from experiment for all molecules except the carbohydrate, allowing the carbohydrate Δ*H*_f_ to be determined. This approach gives excellent agreement with the first method and with the modified HEAT calculations and values in the literature, where available. Δ*H*_f_ for the α- and β-erythrofuranoses and threofuranoses was also determined using the equation in Scheme 1.

### Bond Dissociation Energies

The homolytic bond dissociation energies at 298 K for the carbohydrates were also determined computationally, as shown in Tables 3 and 4. The bond energies were determined by using the lowest-energy conformations of the radicals and carbohydrates. The compounds having two or three carbons were calculated using both CBS-APNO, which has excellent agreement with experiment, and CBS-QB3 methodology. The tetrose radicals were calculated only at the lower level of theory.

**Table 3:** CBS-APNO (CBS-QB3) [Experiment] Bond Energies for Aldoses, kcal/mol

bond	acetaldehyde (21)	glycolaldehyde (5)	glyceraldehyde	erythrose	threose
C1–H	89.2 (89.7) [89.3 ± 0.4]	91.6 (92.2)	92.1 (92.7)	(93.1)	(93.4)
C1–C2	84.6 (85.3) [84.8 ± 0.2]	81.8 (82.9) [82 ± 1]	81.3 (82.4)	(80.7)	(81.4)
C2–H	95.5 (95.3) [95.6 ± 0.4]	79.2 (79.7)	80.1 (80.5)	(80.3)	(81.4)
C2–O		88.6 (89.2)	90.0 (90.4)	(89.8)	(90.5)
C2O–H		111.6 (112.1)	110.7 (110.5)	(108.7)	(108.3)
C2–C3			69.5 (70.6)	(70.1)	(70.8)
C3–H			95.4 (95.8)	(94.3)	(95.0)
C3–O			97.1 (98.2)	(99.7)	(100.5)
C3O–H			110.1 (109.9)	(108.9)	(110.1)
C3–C4				(86.7)	(87.4)
C4–H				(95.6)	(96.1)
C4–O				(97.6)	(99.1)
C4O–H				(107.3)	(107.6)

**Table 4:** CBS-APNO (CBS-QB3) Bond Energies for Ketoses, kcal/mol

bond	dihydroxyacetone	erythrulose
C1–H	80.3 (80.9)	(81.0)
C1–O	88.4 (89.6)	(90.2)
C1O–H	108.1 (109.3)	(110.0)
C1–C2	83.8 (85.3)	(86.4)
C2–C3		(85.3)
C3–H		(82.8)
C3–O		(91.2)
C3O–H		(109.7)
C3–C4		(72.3)
C4–H		(96.5)
C4–O		(98.7)
C4O–H		(108.3)

There are several trends in bond energies reported in Tables 3 and 4. Compared to acetaldehyde, the carbonyl C–H bond in the aldoses is a few kcal/mol stronger. In contrast, the aldoses have much weaker C–H bonds on C2, the α-carbon, compared to acetaldehyde. In addition to delocalizing into the carbonyl, the radicals are stabilized by the OH oxygen; for example, the C2–O bond is 1.41 Å in glyceraldehyde and 1.34 Å in the radical formed by loss of C2–H. The C–O bonds on C2 are also unusually weak due to delocalization. The C1–C2 bond in the aldoses is weaker than in acetaldehyde due to stabilization of the C2 radical by oxygen. The aldose C2–C3 bond is much weaker than the others again because of resonance and stabilizing OH oxygens. Similar trends are apparent for the ketoses but now on both alpha positions.

**Figure 6:**
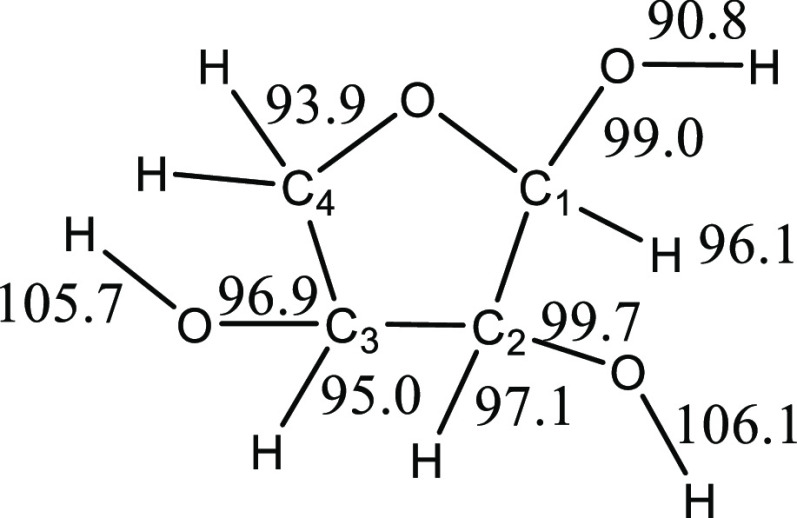
CBS-QB3 bond energies for α-D-erythrofuranose.

The CBS-QB3-calculated C–H and C–O bond energies for α-D-erythrofuranose, the most stable furanose isomer, are listed in Figure 6. The weakest bond is the O–H bond on the anomeric carbon C1, and the radical formed has several notable features. The C1–C2 bond is significantly elongated, from 1.54 Å in the furanose to 3.39 Å in the radical. The bond between the anomeric carbon C1 and the ring oxygen decreases from 1.39 to 1.33 Å in the radical, and the C1–O(H) bond decreases from 1.43 to 1.21 Å. The interaction between the C3O–H and the C1O(H) oxygen also changes significantly, decreasing from 2.13 Å in the furanose to 1.89 Å in the radical. A second conformation of the radical formed via C1O–H bond breaking was obtained and does not exhibit such significant distortions; the bond energy to form this radical is a more typical 106.9 kcal/mol. The C–H bond energies shown in Figure 6 are comparable to those obtained by Taylor, who studied furanoside and pyranoside bond dissociation energies using M06-2X/def2-TZVP calculations.^22^

## Conclusions

Carbohydrates having two to four carbons were studied using CBS-QB3, CBS-APNO, and modified HEAT computational methods. The ketoses are more stable than aldoses but not as much as in simple ketones and aldehydes. This may be due to the influence of the hydroxyl groups on the α-carbon electron density, offset by the differences in the hydrogen bond strengths. The carbonyl reduction of aldoses and ketoses is more exothermic than the carbonyl reduction of simple aldehydes and ketones. Heats of formation of the carbohydrates were obtained using two or three methods with good agreement. Calculated bond dissociation energies for bonds that include the carbons alpha to the carbonyl are decreased by the effects of both resonance into the carbonyl and stabilization by the adjacent oxygen atom.

## Computational Methods

The CBS calculations were completed on a standard PC with a Windows operating system. The molecules and radicals were first calculated using Spartan’08^23^ or Spartan’16.^24^ Initial geometries were optimized using the B3LYP/6-31G* method,^25^ then a conformer distribution was completed at the same level of theory. The structures having relative energies within 3 kcal/mol of the global minimum were brought forward for higher-level calculations.

The structures obtained using the conformer distribution were then calculated using Gaussian 09W^26^ or Gaussian 16W^27^ using the CBS-QB3 method,^28^ and most structures were also calculated using the CBS-APNO methodology.^29^ Each structure was confirmed to be a minimum on the potential energy surface with zero imaginary vibrational frequencies. Radical structures were calculated as doublets. CBS enthalpies at 298 K are reported and in some cases are corrected for higher-energy conformations using a Boltzmann distribution. Select Gaussian 09W CBS-APNO calculations were repeated using Gaussian 16W; the calculated enthalpies were identical or differed by 0.00001H and therefore had no impact on the calculated heats of reduction, heats of formation, or C–H or C–C bond dissociation energies.

The global minimum structure found using the B3LYP/6-31G* calculations was often and not surprisingly different from that obtained using CBS methods. For the smaller molecules, the CBS-QB3 and CBS-APNO calculations gave the same order of stability for the various conformations, with some variation in the relative enthalpies but the same increase in enthalpy due to higher conformations (Supporting Information). For the radicals and polyols having four carbons, only the CBS-QB3 minimum was recalculated using the CBS-APNO method.

The Δ*H*_f_ of glyceraldehyde was also obtained using modified HEAT calculations performed with the CFOUR program system,^30^ which involve a full ab initio coupled cluster analysis, as previously described for glycolaldehyde.^5^

## Data Availability

The data underlying this study are available in the article or the online Supporting Information.

## Supporting Information Available

Cartesian coordinates for calculated structures; calculated enthalpies; and summary of the number of conformations and change in enthalpy due to Boltzmann distributions (PDF)
