# Supplementary material for: Thermochemical Studies of Small Carbohydrates
Source: J Org Chem. 2024 Jan 16;89(3):1769–76. doi: 10.1021/acs.joc.3c02465 (PMC10845155; doi:10.1021/acs.joc.3c02465)
Supplement: Supplementary file 1 — jo3c02465_si_001.pdf [file jo3c02465_si_001.pdf]

Supporting Information

Thermochemical Studies of Small Carbohydrates

Kathleen M. Morgan*† and Joshua H. Baraban‡

†Department of Chemistry, Xavier University of Louisiana, 1 Drexel Drive, New Orleans LA 70125 USA, kmmorgan@xula.edu; ‡Department of Chemistry, Ben-Gurion University of the Negev, Beer Sheva, 841051 Israel

Table of Contents

Table S1. Number of conformations within 3 kcal mol^{-1} of global minimum, included in Boltzmann distributions, and increases in enthalpy, kcal mol^{-1} p. S2

Cartesian Coordinates from AE-CCSD(T) /cc-pVQZ optimizations and modified HEAT ΔH_f for the lowest two conformations of glyceraldehyde p. S3

Cartesian Coordinates (from CBS-APNO calculation if available, otherwise from CBS-QB3 calculation), CBS-QB3 enthalpies and select CBS-APNO enthalpies (Hartrees)

Carbohydrates:
- Glycolaldehyde p. S4
- Glyceraldehyde p. S4
- Dihydroxyacetone p. S8
- Erythulose, acyclic p. S9
- Erythrose, acyclic p. S14
- Erythrose, cyclic p. S22
- Threose, acyclic p. S25
- Threose, cyclic p. S28

Polyols
- Ethylene glycol p. S31
- Glycerol p. S34
- Erythritol p. S45
- Threitol p. S55

Other Compounds, for reductions and isodesmic equations
- 2 carbons p. S58
- 3 carbons p. S59
- 4 carbons p. S63
- 5 carbons p. S76

Radicals
- No carbons p. S78
- 1 carbon p. S79
- 2 carbons p. S79
- 3 carbons p. S81
- 4 carbons p. S85

Table S1. Number of conformations within ~3 kcal mol^{-1} of global minimum, included in Boltzmann distributions, and increases in enthalpy, kcal mol^{-1}

Compound	# conformers, CBS-QB3	increase in enthalpy, CBS-QB3	# conformers, CBS-APNO	increase in enthalpy, CBS-APNO
glycolaldehyde	1	0.0	1	0.0
glyceraldehyde	8	0.3	8	0.3
dihydroxyacetone	3	0.2	3	0.4
erythrulose	11	0.4	12	0.5
erythrose	17	0.7	17	0.7
threose	6	0.3	6	0.3
ethylene glycol	10	0.2	10	0.2
glycerol	26	0.6	26	0.6
erythritol	21	0.6		
threitol	4	0.1		

Modified HEAT results

Glyceraldehyde, global minimum (Vogt conformation I)

ΔHf @ 0 K = -117.04 kcal/mol

ΔHf @ 298 K = -120.62 kcal/mol

Z-matrix Symbol	Atomic Number	Coordinates (in bohr) X	Y	Z
O	8	-3.61243591	-1.02886514	0.70579582
C	6	-1.96865970	-1.16468251	-0.88586133
C	6	-0.01563902	0.90489365	-1.20151436
C	6	2.64520439	-0.19173284	-1.08566275
O	8	-0.29527874	2.71321598	0.73353535
O	8	3.06262543	-1.39665570	1.26372447
H	1	-1.83261934	-2.78833502	-2.18518597
H	1	-0.31464836	1.75920990	-3.07482686
H	1	4.00481500	1.33229425	-1.41905129
H	1	2.89545497	-1.61860919	-2.55636595
H	1	-1.86416673	2.28553250	1.57819613
H	1	2.65405294	-0.15983162	2.53860457

Glyceraldehyde, conformation 2 (Vogt conformation II)

ΔHf @ 0 K = -116.06 kcal/mol H_{rel} = +0.98 kcal/mol

ΔHf @ 298 K = -119.54 kcal/mol H_{rel} = +1.08 kcal/mol

Z-matrix Symbol	Atomic Number	Coordinates (in bohr) X	Y	Z
O	8	-4.25720473	-0.17830323	0.60206060
C	6	-2.53324677	-1.24579048	-0.47230544
C	6	-0.01633101	0.03069720	-0.89391874
O	8	-0.08020690	2.54393814	-0.01253769
C	6	2.08973982	-1.39889556	0.47680939
O	8	4.43079047	-0.17152088	0.11221515
H	1	-2.72606956	-3.20396587	-1.15982976
H	1	0.39525286	0.01201985	-2.92741484
H	1	-1.73139043	2.76032953	0.74909200
H	1	1.60758367	-1.52608005	2.49079687
H	1	2.28271146	-3.31007344	-0.27851167
H	1	4.16513381	1.56990529	0.57888487

CBS-QB3 and CBS-APNO results
Carbohydrates
Glycolaldehyde, global minimum, planar Cs, OH oriented towards carbonyl
H CBS-QB3 -228.71694
H CBS-APNO -228.97709
C,0,0.8283690745,0.4801636448,0.
H,0,1.4293476765,1.410738476,0.
O,0,1.3478299811,-0.6127636671,0.
C,0,-0.6790070889,0.6469887419,0.
H,0,-0.9468559907,1.2429803642,0.8866227074
H,0,-0.9468559907,1.2429803642,-0.8866227074
O,0,-1.3379280234,-0.5894800585,0.
H,0,-0.6328996421,-1.2456078721,0.

Glyceraldehyde, global minimum (Vogt conformation I)
H CBS-QB3 -343.08876
H CBS-APNO -343.47905
C,0,1.0725768411,0.6327894459,0.4169739581
H,0,0.9814925588,1.5505872142,1.0290844552
O,0,1.9750506768,0.4774704597,-0.3735429519
C,0,0.0137951835,-0.4394251798,0.6373638657
H,0,0.151743986,-0.826853155,1.6619010142
C,0,-1.3838778065,0.1564829351,0.4947136018
H,0,-1.5286403314,0.967301186,1.217291428
H,0,-2.1267728256,-0.6278099906,0.6970554796
O,0,0.1623014245,-1.4618552907,-0.321649212
H,0,1.0281835761,-1.3100421181,-0.7184412028
O,0,-1.5412306921,0.7109645428,-0.7954506431
H,0,-1.3116225914,-0.0006100494,-1.3992997928

Glyceraldehyde, conformation 2 (Vogt conformation II)
H CBS-QB3 -343.08668 H_{rel} = +1.31 kcal/mol
H CBS-APNO -343.47729 H_{rel} = +1.10 kcal/mol
C,0,1.3810191297,0.6339129335,0.2056460423
H,0,1.4969205515,1.6869078642,0.5278398052
O,0,2.2771712163,0.0350043994,-0.3465566179
C,0,0.0365878709,-0.0164280119,0.4720512936
H,0,-0.1673507882,0.0409034069,1.5521840448
C,0,-1.0850420213,0.7159519409,-0.2715471988
H,0,-0.8389505241,0.727361967,-1.3456984394
H,0,-1.1741014997,1.7501098876,0.0795817341
O,0,0.0449437957,-1.3661320336,0.0635631482
H,0,0.9088621844,-1.5074186979,-0.340170517

O,0,-2.3219104616,0.0839329054,-0.0303769308
H,0,-2.1711494535,-0.8441065616,-0.2295163644

Glyceraldehyde, conformation 3 (Vogt conformation III)
H CBS-QB3 -343.08518 H_{rel} = +2.25 kcal/mol
H CBS-APNO -343.47579 H_{rel} = +2.05 kcal/mol
C,0,1.3024762642,0.4264126508,0.0290634743
H,0,1.8623048386,1.3455197026,0.2950958622
O,0,1.7771949536,-0.6757485645,0.2004130328
C,0,-0.1100371017,0.6756298027,-0.5029146429
H,0,-0.0296017104,1.2303399421,-1.4473482706
C,0,-0.8706320354,-0.6297950456,-0.7095115258
H,0,-0.3407450186,-1.2776449199,-1.4188850403
H,0,-1.8643913257,-0.4031268301,-1.1053194108
O,0,-0.7838800516,1.5170227913,0.4180004993
H,0,-1.0974501949,0.9193427447,1.1051963613
O,0,-1.0673325096,-1.2718775245,0.5439904341
H,0,-0.2139061085,-1.6450747497,0.7782192266

Glyceraldehyde, conformation 4 (Vogt conformation IV)
H CBS-QB3 -343.08536 H_{rel} = +2.13 kcal/mol
H CBS-APNO -343.47556 H_{rel} = +2.19 kcal/mol
C,0,0.7958520165,-0.9090222888,-0.1591723163
H,0,0.2383865684,-1.8338698654,-0.3834406703
O,0,1.9288096523,-0.9251690178,0.2728736589
C,0,0.0733301357,0.4065437271,-0.4137614876
H,0,-0.1624530299,0.4437425678,-1.4896776651
C,0,-1.2444761281,0.4519759131,0.3593777976
H,0,-1.7119452533,1.4293673372,0.1811343645
H,0,-1.0261601454,0.361328057,1.4338647555
O,0,0.8744951053,1.4935802293,-0.0314727483
H,0,1.7169545291,1.1033623131,0.2285293671
O,0,-2.0445811111,-0.6209676588,-0.1152596577
H,0,-2.8962123397,-0.5588713138,0.3170046016

Glyceraldehyde, conformation 5 (Vogt conformation V)
H CBS-QB3 -343.08474 H_{rel} = +2.52 kcal/mol
H CBS-APNO -343.47513 H_{rel} = +2.46 kcal/mol
C,0,0.7931653685,-0.9064647574,-0.1385588127
H,0,0.2238744396,-1.8390800859,-0.294355869
O,0,1.9486608257,-0.9066638739,0.2275576233
C,0,0.0504569385,0.3959126131,-0.3971349382
H,0,-0.2089935657,0.4074735808,-1.4714955389

C,0,-1.2517437004,0.4384956644,0.4138627701
H,0,-1.6989141614,1.4349465256,0.3051781677
H,0,-1.0228069925,0.2857816776,1.4731318728
O,0,0.8506653872,1.4984232401,-0.0630541074
H,0,1.715696004,1.1271334484,0.1456946176
O,0,-2.1382493122,-0.600010301,0.0277619266
H,0,-2.4948112314,-0.3619477316,-0.8295877119

Glyceraldehyde, conformation 6 (Vogt conformation VI)
H CBS-QB3 -343.08456 H_{rel} = +2.64 kcal/mol
H CBS-APNO -343.47479 H_{rel} = +2.67 kcal/mol
C,0,-0.1897757761,-1.2693541512,-0.1112502018
H,0,0.863855362,-1.536673941,0.100359435
O,0,-0.9522089201,-2.0436969968,-0.6408188081
C,0,-0.6321862741,0.1261471537,0.3135971664
H,0,-0.5265051287,0.1749892091,1.4111046631
C,0,0.3012460189,1.1641460098,-0.3003420768
H,0,1.2869928572,1.1059341876,0.1898028508
H,0,-0.1285421635,2.157149445,-0.115530642
O,0,-1.9653684284,0.3546666158,-0.0550354266
H,0,-2.2368615313,-0.4398576222,-0.5277098984
O,0,0.3948217952,0.8740384808,-1.6851867658
H,0,0.7751654288,1.6368711094,-2.1196674159

Glyceraldehyde, conformation 7 (Vogt conformation 8)
H CBS-QB3 -343.0838 H_{rel} = +3.11 kcal/mol
H CBS-APNO -343.47427 H_{rel} = +3.00 kcal/mol
C,0,1.3074706022,0.3254635772,0.2628656711
H,0,1.5037376802,1.0515157449,1.0794224141
O,0,2.0682978654,-0.5730524921,-0.0052809619
C,0,0.0061170953,0.5791431054,-0.5102704828
H,0,0.2879022616,0.9455513136,-1.5052054477
C,0,-0.782285964,-0.7153860696,-0.6505290112
H,0,-0.131324307,-1.496469674,-1.0612585798
H,0,-1.6401217351,-0.5484806009,-1.315278519
O,0,-0.7657456987,1.5864783563,0.1074426775
H,0,-1.223120388,1.1437058425,0.8282444836
O,0,-1.2139024864,-1.0287478297,0.672387106
H,0,-1.7760249256,-1.8017212735,0.6254606499

Glyceraldehyde, conformation 8 (Vogt conformation 7)
H CBS-QB3 -343.08358 H_{rel} = +3.25 kcal/mol
H CBS-APNO -343.47372 H_{rel} = +3.34 kcal/mol

C,0,1.5483226418,0.2277397014,-0.0436910775
H,0,2.0443136854,1.1785792172,0.2377028103
O,0,2.1428529012,-0.8235332931,-0.0966946197
C,0,0.0673790724,0.3652767862,-0.3643468538
H,0,-0.0357420036,0.3910442358,-1.4627448823
C,0,-0.7306725613,-0.8176452927,0.1639784756
H,0,-0.5885026458,-0.8805161349,1.2522406406
H,0,-0.3757675109,-1.7442490515,-0.302089914
O,0,-0.3719402419,1.5770406164,0.2144338137
H,0,-1.3299712227,1.5533462931,0.1491410856
O,0,-2.0892777885,-0.5376132158,-0.1605577483
H,0,-2.6349943262,-1.166469862,0.3116282699

Glyceraldehyde, Figure 5 conformation with C2 OH hydrogen bonded to carbonyl oxygen
H CBS-QB3 -343.08181 H_{rel} = 0 kcal/mol
H CBS-APNO -343.47223 H_{rel} = 0 kcal/mol
C,0,-0.1495539774,0.9563495497,-0.607511836
H,0,-0.1257169271,2.002733059,-0.244526998
O,0,-0.2826103835,0.695903881,-1.7821790921
C,0,0.0131845844,-0.1328884657,0.4416638328
H,0,1.0016817123,0.0247692792,0.9074192433
C,0,-1.0313528536,0.0228418993,1.5416336725
H,0,-1.9941257599,-0.3187712972,1.1362080819
H,0,-1.1185017996,1.0855463553,1.8235191137
O,0,-0.0633685961,-1.4039315358,-0.14716571
H,0,-0.1131500479,-1.2371993647,-1.0949886582
O,0,-0.6133306289,-0.7602985594,2.6439452577
H,0,-1.3865546026,-0.9321942107,3.1810602123

Glyceraldehyde, Figure 5 conformation with C2 OH hydrogen bonded to C3 OH
H CBS-QB3 -343.07865 H_{rel} = +1.98 kcal/mol
H CBS-APNO -343.46913 H_{rel} = +1.95 kcal/mol
C,0,1.4188069392,0.5648288008,0.2342305274
H,0,1.5059958499,1.5760627618,0.6932251458
O,0,2.3307410262,0.0672608174,-0.3748098972
C,0,0.0612029827,-0.0845805676,0.4496352073
H,0,-0.1364613374,-0.0635742129,1.5369700727
C,0,-1.0239837932,0.7464580341,-0.2325562992
H,0,-0.8203240636,0.7580844309,-1.3126637296
H,0,-1.024654931,1.7770714974,0.1511183201
O,0,0.0697852356,-1.3937150654,-0.0526383326
H,0,-0.852941392,-1.6617936069,-0.0470127581
O,0,-2.2523691303,0.0861689537,0.0506350108

H,0,-2.9055459858,0.4092056167,-0.5703067576

Dihydroxyacetone, C2 global minimum
H CBS-QB3 -343.09234
H CBS-APNO -343.48219
C,0,0.0000001235,0.000000062,0.0515360627
C,0,-0.0656257732,1.306593548,-0.7223020359
H,0,0.6416361056,1.2718393066,-1.5603482525
H,0,-1.081438756,1.3649600547,-1.1504002935
C,0,0.0656253914,-1.3065936408,-0.7223017117
H,0,1.0814380805,-1.3649603208,-1.1504006522
H,0,-0.641637066,-1.2718396418,-1.5603474422
O,0,0.0000006946,0.0000002502,1.2674913771
O,0,0.2291864555,2.4091484068,0.0915126537
H,0,0.0308675809,2.1224421866,0.9885686439
O,0,-0.2291863747,-2.4091482551,0.0915134836
H,0,-0.0308664622,-2.1224419609,0.988569217

Dihydroxyacetone, conformer 2
H CBS-QB3 -343.08907 H_{rel} = +2.05 kcal/mol
H CBS-APNO -343.47949 H_{rel} = +1.69 kcal/mol
C,0,-0.5951740268,0.0497943047,0.2698176066
O,0,-1.7020310647,0.2118809996,0.7426688001
C,0,0.2232344506,1.237833982,-0.2126590894
H,0,1.1757669062,1.2387185297,0.3329762745
H,0,0.4627740066,1.0746617372,-1.2734601355
C,0,-0.0141528437,-1.3466100774,0.1091340575
H,0,-0.5718302261,-1.8229903854,-0.7116684427
H,0,-0.2204716633,-1.903150382,1.0330701319
O,0,-0.4769258341,2.4394458816,-0.0253741808
H,0,-1.3195539719,2.1799618699,0.3627358793
O,0,1.3692055287,-1.2478910772,-0.1723549579
H,0,1.6691587385,-2.1116563827,-0.4548859436

Dihydroxyacetone, conformer 3
H CBS-QB3 -343.08828 H_{rel} = +2.55 kcal/mol
H CBS-APNO -343.47883 H_{rel} = +2.11 kcal/mol
C,0,-0.592205527,-0.0825734319,-0.2195867672
O,0,-1.7698660334,-0.0581415309,-0.5165555328
C,0,0.1818945343,1.2159052212,-0.0339023499
H,0,0.6276258468,1.2208345086,0.9699707767
H,0,1.0167160052,1.2109342755,-0.7506952891
C,0,0.1588306898,-1.4042860325,-0.0858439482

H,0,0.3430913891,-1.7690475744,-1.1027875178
H,0,-0.5042211182,-2.1253462227,0.4097126713
O,0,-0.6512206597,2.3291496085,-0.2123871255
H,0,-1.5002917889,1.963934454,-0.4848743273
O,0,1.4210776216,-1.2865623393,0.5418006231
H,0,1.2685710402,-1.2148009361,1.4851477868

Erythrulose, acyclic, global minimum
H CBS-QB3 -457.46500
H CBS-APNO -457.98531
C,0,-1.0633353223,1.3458252595,-0.1742629352
H,0,-0.1301902471,1.9246320998,-0.0779201711
H,0,-1.2883967521,1.2426646528,-1.242955872
C,0,-0.771469411,-0.0175995133,0.4282217055
C,0,0.1062214915,-1.0065434354,-0.3354756648
H,0,-0.4816180155,-1.3615077688,-1.199262925
C,0,1.3947894586,-0.3455605467,-0.8172147065
H,0,1.1831046451,0.4655395577,-1.520592497
H,0,2.0015761296,-1.1024778475,-1.3330891763
O,0,-1.2235759334,-0.321554703,1.5160724731
O,0,-2.14623919,1.9709745663,0.4620637937
H,0,-2.1655085007,1.6058103919,1.3519472825
O,0,0.4678774685,-2.075240898,0.5107269523
H,0,-0.157251202,-2.0430870001,1.2440115268
O,0,2.0835480812,0.2197163296,0.2808780521
H,0,2.1914672997,-0.5035911449,0.9048521617

Erythrulose, acyclic, conformation 2
H CBS-QB3 -457.46263 H_{rel} = +1.49 kcal/mol
H CBS-APNO -457.98312 H_{rel} = +1.37 kcal/mol
C,0,-1.4021369926,0.9688537948,0.6980960835
H,0,-0.8490794187,1.8559368526,0.343098015
H,0,-2.120461233,0.6944553593,-0.0838479542
C,0,-0.380175665,-0.1440818918,0.8663894222
C,0,0.2304220509,-0.7873791596,-0.3689347822
H,0,-0.5777806718,-1.2708767086,-0.9386016025
C,0,0.9113962127,0.2500052284,-1.2697742456
H,0,1.6346425899,0.8170975799,-0.6607144985
H,0,0.1785420382,0.9493078519,-1.6861334536
O,0,-0.0282959833,-0.4941943996,1.9779616883
O,0,-2.0895134037,1.2251997799,1.8923641547
H,0,-1.5046505992,0.9261864688,2.595577476
O,0,1.1930569121,-1.750952878,-0.0027311272

H,0,1.1961017363,-1.7641989088,0.9610707989
O,0,1.5375193534,-0.388919939,-2.3575072802
H,0,2.0694130738,-1.0874390302,-1.9663126945

Erythrulose, acyclic, conformation 3
H CBS-QB3 -457.46202 H_{rel} = +1.87 kcal/mol
H CBS-APNO -457.98266 H_{rel} = +1.66 kcal/mol
C,0,1.239420828,-0.0896930137,1.409683723
H,0,2.105639522,0.5125176274,1.1096562792
H,0,0.49136343,0.5997077272,1.832033217
C,0,0.6164284165,-0.7045842441,0.1680911672
C,0,0.2113086687,0.2156161928,-0.9896993144
H,0,0.7679566451,-0.1234879185,-1.8708748154
C,0,-1.2836370654,0.0698570317,-1.2452967659
H,0,-1.5342750752,-0.9898437085,-1.3712180808
H,0,-1.5515737259,0.633774735,-2.1485435284
O,0,0.4308559781,-1.9037980013,0.0982280911
O,0,1.6097461937,-1.078636418,2.3333704947
H,0,1.2536854844,-1.8991554156,1.975435151
O,0,0.5537291451,1.5668914037,-0.7622746711
H,0,-0.1551497346,1.9201568233,-0.2164597915
O,0,-1.9047902914,0.6286113898,-0.0887551418
H,0,-2.8507084191,0.6420657889,-0.2343760138

Erythrulose, acyclic, conformation 4
H CBS-QB3 -457.46164 H_{rel} = +2.11 kcal/mol
H CBS-APNO -457.98225 H_{rel} = +1.92 kcal/mol
C,0,1.1184289862,-1.455809829,0.7575825904
H,0,0.4978318617,-2.3530988842,0.644861683
H,0,1.8052853974,-1.4295488677,-0.1018963466
C,0,0.2370695825,-0.2189507381,0.657382398
C,0,-0.7969638167,-0.1735127524,-0.4721284639
H,0,-1.6116225687,-0.8596690827,-0.2027230845
C,0,-1.3365867707,1.2383377431,-0.6714729253
H,0,-1.8092239109,1.6057498002,0.2464920034
H,0,-2.0754646439,1.2219362511,-1.4774545073
O,0,0.3858622578,0.7021451789,1.4412605077
O,0,1.8021029105,-1.4986066273,1.9830823466
H,0,1.7986016577,-0.5923603257,2.3068390869
O,0,-0.2181378116,-0.666718691,-1.668442742
H,0,0.2644979702,0.0833762241,-2.0323045612
O,0,-0.2811969635,2.0903274072,-1.1006116026
H,0,0.219515862,2.3064031936,-0.3104663827

Erythrulose, acyclic, conformation 5
H CBS-QB3 -457.46143 H_{rel} = +2.24 kcal/mol
H CBS-APNO -457.98219 H_{rel} = +1.96 kcal/mol
C,0,1.25809536,-0.3489125449,1.5803242218
H,0,2.1651289534,0.2026506662,1.2955190606
H,0,0.6019829096,0.3649474498,2.0962245627
C,0,0.5600871546,-0.7880323178,0.3037678974
C,0,0.1487317552,0.2698006818,-0.7092429795
H,0,0.697819119,0.0602007218,-1.6409227989
C,0,-1.3452305757,0.1624768749,-0.9981261977
H,0,-1.8980277683,0.2910455682,-0.0558679048
H,0,-1.5694012919,-0.8226456071,-1.4231272161
O,0,0.3303728197,-1.9645670101,0.0964932801
O,0,1.5573009971,-1.4512773936,2.3954703842
H,0,1.2494331994,-2.2191908062,1.9020485885
O,0,0.4687507398,1.5498699695,-0.2064420312
H,0,-0.0102353805,2.1651153326,-0.7683503051
O,0,-1.6320223807,1.2201041646,-1.9063095469
H,0,-2.5837856106,1.3074142502,-1.9614590153

Erythrulose, acyclic, conformation 6
H CBS-QB3 -457.46104 H_{rel} = +2.48 kcal/mol
H CBS-APNO -457.98192 H_{rel} = +2.13 kcal/mol
C,0,-0.0576399816,-1.9539151749,0.6728113914
H,0,0.5173349541,-1.9804747948,1.6114113966
H,0,-0.9310764147,-2.59974442,0.8055222567
C,0,-0.5694799175,-0.5226018998,0.5002460111
C,0,0.0295128753,0.2790997468,-0.6639871245
H,0,-0.2070765116,-0.2286106699,-1.6051688091
C,0,-0.4673007996,1.7182219348,-0.6724091409
H,0,-1.5605250088,1.7456047874,-0.7563845972
H,0,-0.0287730414,2.2426077107,-1.526067358
O,0,-1.3591548195,-0.0379517179,1.2882366529
O,0,0.6737381061,-2.4407985458,-0.4282254121
H,0,1.4315664513,-1.8570582879,-0.5244768886
O,0,1.4498147254,0.2482877024,-0.5172919439
H,0,1.6427569379,0.9564105582,0.1077725236
O,0,-0.0099556785,2.3863617819,0.4965438817
H,0,-0.5537418767,2.0445612888,1.2114671605

Erythrulose, acyclic, conformation 7
H CBS-QB3 -457.46091 H_{rel} = +2.57 kcal/mol

H CBS-APNO -457.98189 H_{rel} = +2.15 kcal/mol
C,0,-0.7326626312,1.0108580432,1.5786108644
H,0,-0.2527593451,0.5027725632,2.4265990132
H,0,-0.0318792991,1.7844231003,1.2355183253
C,0,-0.8958804595,-0.0053963976,0.4609683604
C,0,0.3444070556,-0.6784882688,-0.1055862326
H,0,0.1641343023,-1.7660272203,-0.0760423141
C,0,0.5446056771,-0.2515228756,-1.5640483938
H,0,0.6445376597,0.837748432,-1.6109415926
H,0,-0.3151324855,-0.5596468196,-2.1701044205
O,0,-1.9951715365,-0.2608032373,0.0070322842
O,0,-1.9696571784,1.5663615294,1.9392752965
H,0,-2.6183124768,1.1172934836,1.3866348573
O,0,1.4732940067,-0.3284436967,0.6648296633
H,0,2.2285537596,-0.4791649907,0.088687303
O,0,1.7732607237,-0.7768788148,-2.0515130723
H,0,1.6366622275,-1.7130848302,-2.2119199418

Erythrulose, acyclic, conformation 8
H CBS-QB3 -457.46135 H_{rel} = +2.29 kcal/mol
H CBS-APNO -457.98125 H_{rel} = +2.55 kcal/mol
C,0,0.8815956595,-1.2212964312,0.9777044834
H,0,-0.1172125978,-1.6665225366,0.9758693051
H,0,1.521211961,-1.8226369775,0.3073510977
C,0,0.8055568191,0.1568833168,0.3517322332
C,0,0.0965144709,0.3537793708,-0.988971481
H,0,0.2439642993,-0.5326531753,-1.6180049074
C,0,-1.4076570893,0.5544285621,-0.7689986783
H,0,-1.8563512615,0.7665138116,-1.7481317035
H,0,-1.5465303845,1.4321817531,-0.1196908036
O,0,1.334723253,1.1145581082,0.8878830514
O,0,1.3876207298,-1.1741484902,2.2858151168
H,0,1.8645789971,-0.3407899044,2.3456327081
O,0,0.6260772845,1.4775746812,-1.6510582969
H,0,0.98476042,2.0353686245,-0.9519437698
O,0,-1.9356638042,-0.6259790041,-0.1846700898
H,0,-2.882188757,-0.5092617091,-0.1005182655

Erythrulose, acyclic, conformation 9
H CBS-QB3 -457.46085 H_{rel} = +2.60
H CBS-APNO -457.98108 H_{rel} = +2.65
C,0,0.6153757881,-1.0316382828,1.1676604383
H,0,-0.3872749603,-0.9651923235,1.6163640297

H,0,0.6028613331,-1.8913979249,0.4893723241
C,0,0.8354178819,0.2459627228,0.3726409457
C,0,0.0765644358,0.4980465513,-0.9296399936
H,0,0.379681679,-0.2934203789,-1.63740904
C,0,-1.4457017307,0.4221630342,-0.7364031419
H,0,-1.9140940794,0.8385439236,-1.6369330062
H,0,-1.7367896167,1.0477903889,0.1134416084
O,0,1.6153110366,1.0896584047,0.7791511383
O,0,1.6219974488,-1.2103739796,2.13054752
H,0,1.9679368082,-0.3305134116,2.3075076215
O,0,0.3943300945,1.7673018913,-1.4390022946
H,0,1.0984443651,2.1016308435,-0.8716853683
O,0,-1.895556585,-0.8925613364,-0.4558462546
H,0,-1.8285038992,-1.3950001225,-1.2697665268

Erythrulose, acyclic, conformation 10
H CBS-QB3 -457.45960 H_{rel} = +3.39 kcal/mol
H CBS-APNO -457.98061 H_{rel} = +2.95 kcal/mol
C,0,1.332671012,-0.5031460856,1.2133563632
H,0,1.2289451842,-1.2985106798,1.9615035128
H,0,2.3986588192,-0.3520978457,1.0091219857
C,0,0.6720985737,-1.0014454047,-0.0774199195
C,0,0.1693763693,0.0423488369,-1.0933438219
H,0,0.366441601,-0.3784457515,-2.0850456961
C,0,-1.3459123887,0.1739505035,-0.9453971345
H,0,-1.8134900999,-0.7768125323,-1.2336575603
H,0,-1.6904073339,0.9800425575,-1.6049214459
O,0,0.536769791,-2.1853965629,-0.2970807808
O,0,0.822774951,0.7391192037,1.6722372231
H,0,-0.1370149567,0.656484865,1.6817291098
O,0,0.8525552075,1.2731811963,-1.0273766402
H,0,0.8227101585,1.5521571714,-0.1053855729
O,0,-1.6351568318,0.4785648955,0.4232243039
H,0,-2.5810200564,0.6000056328,0.5074560735

Erythrulose, acyclic, conformation 11
H CBS-QB3 -457.46017 H_{rel} = +3.03 kcal/mol
H CBS-APNO -457.98047 H_{rel} = +3.04 kcal/mol
C,0,-1.534666768,-1.0934580545,0.6388339521
H,0,-2.0616447834,-1.2740483994,-0.3063235462
H,0,-0.9212035357,-1.9842872305,0.8391640066
C,0,-0.574853404,0.0690552006,0.4496506046
C,0,0.3508189084,0.0565089367,-0.7608018498

H,0,-0.1764366196,0.617138315,-1.5502917928
C,0,1.6722652969,0.7791656748,-0.4689992781
H,0,2.3455210716,0.6297739428,-1.3207726066
H,0,2.1398058379,0.3227374345,0.4163156637
O,0,-0.5539700087,0.9833439086,1.2561246041
O,0,-2.4530381866,-0.8362350142,1.667792013
H,0,-2.0870694497,-0.0927849011,2.1574746016
O,0,0.5458276638,-1.3014350151,-1.1211559666
H,0,0.9160318085,-1.3156752195,-2.0049427493
O,0,1.4866957178,2.1692952839,-0.3248981922
H,0,0.9029164508,2.2699051374,0.4318305359

Erythrulose, acyclic, conformation 12
H CBS-QB3 -457.45923 H_{rel} = +3.62 kcal/mol
H CBS-APNO -457.98017 H_{rel} = +3.23 kcal/mol
C,0,1.3480258899,1.221237961,-0.101201033
H,0,1.2595476331,1.2545019864,-1.1991286181
H,0,1.722954874,2.1842052854,0.2546965553
C,0,-0.0257025594,0.9897546339,0.5239993161
C,0,-1.0467734642,0.1225355554,-0.2313160149
H,0,-1.4470958193,0.7426766709,-1.052852899
C,0,-0.4212325254,-1.1414490634,-0.8086116266
H,0,0.3213358973,-0.9052427775,-1.5760766645
H,0,-1.2117197357,-1.7526515121,-1.2610669559
O,0,-0.3360521552,1.4467596992,1.6051118085
O,0,2.2570421165,0.2227372674,0.3239809041
H,0,1.7878010173,-0.6192529327,0.3428866136
O,0,-2.0660630272,-0.279461876,0.6556510293
H,0,-2.016404982,0.3437582663,1.3907323661
O,0,0.2591571622,-1.8579577101,0.2143155487
H,0,-0.3858203221,-1.9731514539,0.9188796702

Erythrose, acyclic, global minimum
H CBS-QB3 -457.46058
H CBS-APNO -457.98094
C,0,1.2876471082,0.8739937415,0.67320791
H,0,2.0554711318,0.1288587609,0.421615242
O,0,1.3709468028,1.5833964844,1.6534741136
C,0,0.1100974798,1.0312353167,-0.2887384821
H,0,0.5342534655,1.2944685285,-1.2724319741
C,0,-0.7544491688,-0.2307537532,-0.4445952621
H,0,-1.5031854914,-0.0025857035,-1.2192822682
C,0,0.0003810829,-1.492789853,-0.8470699719

H,0,-0.734960267,-2.2602271199,-1.126940974
H,0,0.6379041088,-1.2956131942,-1.7161237665
O,0,-0.7405509175,2.055480281,0.1824429265
H,0,-0.2598777228,2.4542918828,0.9192605255
O,0,-1.3881069243,-0.5224384516,0.7890590351
H,0,-1.727172157,0.3204336776,1.1046405209
O,0,0.8393537235,-1.9523117549,0.1974032687
H,0,0.2732477455,-1.984438843,0.9740791565

Erythrose, acyclic, conformation 2
H CBS-QB3 -457.45956 H_{rel} = +0.64 kcal/mol
H CBS-APNO -457.97987 H_{rel} = +0.67 kcal/mol
C,0,2.1152733122,0.2755347702,-0.3321870027
H,0,2.181011002,1.0419118296,-1.1234791522
O,0,3.0587851716,-0.4164701002,-0.0146486279
C,0,0.7657021989,0.1171803972,0.3453137746
H,0,0.5524222615,1.0662759627,0.8652771569
C,0,-0.3390595834,-0.0818337511,-0.7090461836
H,0,-0.0307435482,-0.8497155129,-1.4298135415
C,0,-1.6492284185,-0.505958888,-0.0529185719
H,0,-2.4250638952,-0.5486494621,-0.8235961774
H,0,-1.5423983105,-1.4911727505,0.4117523182
O,0,0.7905529086,-0.9549174321,1.2551673338
H,0,1.713168157,-1.234371386,1.2938078963
O,0,-0.5017977209,1.1263428103,-1.4339679392
H,0,-1.0176707687,1.697624047,-0.8560365402
O,0,-2.0582780073,0.4816479197,0.8898516272
H,0,-1.6136747591,0.2745715462,1.7145236296

Erythrose, acyclic, conformation 3
H CBS-QB3 -457.45932 H_{rel} = +0.79 kcal/mol
H CBS-APNO -457.97962 H_{rel} = +0.83 kcal/mol
C,0,2.1952883968,-0.0843964868,-0.2471679165
H,0,2.5307905982,0.7715632208,0.361251188
O,0,2.9216961085,-0.6342038204,-1.0482272523
C,0,0.762836475,-0.551505392,-0.0420823273
H,0,0.6666112919,-0.8830160879,1.004157533
C,0,-0.2139724687,0.6179596422,-0.2618510849
H,0,-0.0931026878,0.9777563129,-1.290550045
C,0,-1.6545617092,0.1708434435,-0.057109988
H,0,-2.322908046,0.9967493957,-0.3368034333
H,0,-1.8605925828,-0.7087353465,-0.6771149225
O,0,0.4596169209,-1.6003104923,-0.9288142

H,0,1.2891032211,-1.7904409593,-1.3831757003
O,0,0.0981994543,1.6926514344,0.608398914
H,0,-0.319506816,1.4629401008,1.4441743934
O,0,-1.7696632305,-0.1235411997,1.3352618612
H,0,-2.6888349257,-0.3153137653,1.5196529807

Erythrose, acyclic, conformation 4
H CBS-QB3 -457.45896 H_{rel} = +1.02 kcal/mol
H CBS-APNO -457.97924 H_{rel} = +1.07 kcal/mol
C,0,-0.1004040435,-1.5601306448,-1.6556257156
H,0,0.4421819155,-2.4026350679,-1.1965379721
O,0,-0.25099136,-1.4582976889,-2.8544687026
C,0,-0.6627371858,-0.5078912318,-0.7090374195
H,0,-1.3887077011,-1.0181181802,-0.0535228565
C,0,0.4493430259,0.0557362983,0.1767915671
H,0,1.2163658898,0.5141704972,-0.4680331591
C,0,-0.0765800695,1.1203822255,1.1268747206
H,0,-0.452845591,1.9780970187,0.5581191722
H,0,-0.8912031465,0.6856793117,1.7252578135
O,0,-1.2757583128,0.5281348042,-1.4331451063
H,0,-1.2303837404,0.2494983827,-2.3556344674
O,0,0.9854694076,-1.0343811877,0.9068058794
H,0,1.5077066864,-0.6329095248,1.6065496178
O,0,1.0314402029,1.4717607195,1.9542557505
H,0,0.6991040226,2.0129042684,2.6703508778

Erythrose, acyclic, conformation 5
H CBS-QB3 -457.45867 H_{rel} = +1.20 kcal/mol
H CBS-APNO -457.97921 H_{rel} = +1.09 kcal/mol
C,0,0.5093811958,1.1985964511,-1.5254542253
H,0,1.5155864692,0.9804609293,-1.9320635797
O,0,0.0566773624,2.321929806,-1.5137813099
C,0,-0.287117115,0.0124425947,-1.0049468366
H,0,-0.4596727961,-0.6616924708,-1.859601196
C,0,0.5108317916,-0.7872140181,0.0425931706
H,0,1.5462818535,-0.8685536273,-0.31754352
C,0,0.5010650233,-0.1009000218,1.4164251881
H,0,1.258008404,-0.566170149,2.0541269127
H,0,0.7366113818,0.9697409106,1.3172497267
O,0,-1.5193428382,0.467752064,-0.4773320873
H,0,-1.5867463806,1.3902543593,-0.7538200709
O,0,0.0144752423,-2.1028854199,0.1614594468
H,0,-0.6554940595,-2.0518265374,0.8514127362

O,0,-0.7462853478,-0.3066626028,2.0602047377
H,0,-1.3942601866,0.1037277323,1.478070907

Erythrose, acyclic, conformation 6
H CBS-QB3 -457.45889 H_{rel} = +1.06 kcal/mol
H CBS-APNO -457.97919 H_{rel} = +1.10 kcal/mol
C,0,0.0480222093,-1.1153342573,-1.4074936951
H,0,-0.9746025724,-1.2408630494,-1.8009348549
O,0,0.8463506239,-2.0266508353,-1.3750642147
C,0,0.4516565704,0.2845253966,-0.9663434447
H,0,0.3979187923,0.9323465561,-1.8569081928
C,0,-0.4658102251,0.9015767832,0.0996060472
H,0,-1.4681121214,1.0464730287,-0.320776359
C,0,-0.5843759623,0.011942412,1.332357655
H,0,0.4230585797,-0.218373593,1.7044931378
H,0,-1.137179531,0.5587417706,2.1065072011
O,0,1.7703904011,0.2705292551,-0.4639718959
H,0,2.0821851423,-0.631738111,-0.6065260842
O,0,0.0260371998,2.1796576372,0.451429059
H,0,0.9680620594,2.0572692356,0.602466637
O,0,-1.2750160251,-1.1651284803,0.9248002911
H,0,-1.1095851409,-1.8459737489,1.5773587131

Erythrose, acyclic, conformation 7
H CBS-QB3 -457.45871 H_{rel} = +1.17 kcal/mol
H CBS-APNO -457.97904 H_{rel} = +1.19 kcal/mol
C,0,0.2283840038,-0.7409324932,-2.1037982182
H,0,1.2371451246,-1.1844527153,-2.0802141846
O,0,-0.299752579,-0.3779164586,-3.1331185243
C,0,-0.4909147307,-0.5975159722,-0.7690665987
H,0,-0.6053802078,-1.6123690344,-0.3519944128
C,0,0.3555712018,0.2173839661,0.2098306786
H,0,0.4914608551,1.2279732434,-0.2147245943
C,0,-0.3165686658,0.3467748922,1.5726497931
H,0,-1.2694230117,0.8800662067,1.483951434
H,0,-0.4991011441,-0.6540899874,1.9778417407
O,0,-1.7392821254,0.0226357872,-0.9459931005
H,0,-1.8361534721,0.1227589345,-1.900641301
O,0,1.6007386076,-0.4433023525,0.3451013157
H,0,1.9762038746,-0.1061474433,1.1636616789
O,0,0.577131012,0.9829694923,2.4853722807
H,0,0.5899412571,1.9151639343,2.2591420126

Erythrose, acyclic, conformation 8
H CBS-QB3 -457.45796 H_{rel} = +1.64 kcal/mol
H CBS-APNO -457.97840 H_{rel} = +1.59 kcal/mol
C,0,0.5290392825,0.8103586171,1.5789860356
H,0,0.9578382129,-0.1301474739,1.9689659217
O,0,0.113721327,1.67267094,2.3199416747
C,0,0.5162566051,0.9989755019,0.0658216758
H,0,1.5678212164,1.0289793971,-0.2688233985
C,0,-0.2193881449,-0.1343012584,-0.652848041
H,0,-0.2438942981,0.1167317318,-1.7233167021
C,0,0.3928970342,-1.5208081292,-0.5125089948
H,0,1.4248970403,-1.5146529045,-0.879663396
H,0,0.3978483708,-1.8236252477,0.5456718695
O,0,-0.1524197114,2.1995079229,-0.2533750345
H,0,-0.2370161547,2.6716908017,0.5835198988
O,0,-1.5430416523,-0.2307264262,-0.1422453092
H,0,-1.9385957062,0.6345988546,-0.2762185036
O,0,-0.3234804811,-2.4520194017,-1.2978974956
H,0,-1.2444829404,-2.3272329255,-1.055010201

Erythrose, acyclic, conformation 9
H CBS-QB3 -457.45813 H_{rel} = +1.54 kcal/mol
H CBS-APNO -457.97822 H_{rel} = +1.71 kcal/mol
C,0,-0.1733382831,2.1443508535,-0.3977399371
H,0,0.140557572,2.7561793432,-1.265833765
O,0,-0.9066901564,2.5793999277,0.4648574932
C,0,0.3919612344,0.7333016033,-0.3631438904
H,0,1.239886883,0.7519367019,0.3373018873
C,0,-0.6393748425,-0.2568207293,0.2106333616
H,0,-1.611349216,-0.1068939002,-0.2850710764
C,0,-0.1899377753,-1.6931017005,-0.0078490061
H,0,-0.8750825954,-2.3615519108,0.530518427
H,0,-0.2238648932,-1.9387111414,-1.0720736723
O,0,0.811959499,0.4110493192,-1.6733513588
H,0,1.4557713177,-0.2949981489,-1.5814194145
O,0,-0.7455754045,-0.0620108151,1.6129842575
H,0,-1.000480369,0.8579361632,1.7322879563
O,0,1.1559389695,-1.875696643,0.41226516
H,0,1.1686180598,-1.6443689228,1.3446335778

Erythrose, acyclic, conformation 10
H CBS-QB3 -457.45729 H_{rel} = +2.06 kcal/mol
H CBS-APNO -457.97814 H_{rel} = +1.76 kcal/mol

C,0,0.6793721039,-0.1174168354,-1.6640113595
H,0,1.6228244074,-0.1685184468,-2.2426927945
O,0,-0.3221962988,-0.6834454716,-2.0472376757
C,0,0.7886392425,0.7622761416,-0.415300564
H,0,0.4018201863,1.7505432161,-0.7047734435
C,0,0.0407987299,0.2677480695,0.8333685424
H,0,0.2331191897,1.0328023039,1.5965913439
C,0,-1.4793791071,0.068419573,0.7160769937
H,0,-1.9070786717,0.1173111797,1.7216224534
H,0,-1.9250717325,0.8696473347,0.1097956456
O,0,2.1565532453,0.9324702451,-0.0981680413
H,0,2.3890478801,0.1501548612,0.413200539
O,0,0.6569427757,-0.9327030822,1.2763425251
H,0,0.0511280047,-1.632925546,1.005137176
O,0,-1.8023128609,-1.2182595185,0.2185536898
H,0,-1.5852070946,-1.1991040244,-0.7195050305

Erythrose, acyclic, conformation 11
H CBS-QB3 -457.45716 H_{rel} = +2.15 kcal/mol
H CBS-APNO -457.97785 H_{rel} = +1.94 kcal/mol
C,0,0.2793837547,-0.7918180386,-1.8030934386
H,0,-0.2337772857,-0.165037251,-2.557327475
O,0,0.2968646992,-2.0001289321,-1.8792297816
C,0,0.9584539531,-0.0608104003,-0.6581571597
H,0,1.7038605854,0.6251341838,-1.0896075729
C,0,-0.0381405162,0.7799480132,0.1526111013
H,0,-0.4304149135,1.5964770363,-0.4654333521
C,0,-1.2142189406,-0.0313700234,0.6977450525
H,0,-1.8300174534,-0.4237286713,-0.1205904416
H,0,-0.8255560054,-0.8837234267,1.2755206687
O,0,1.6012326297,-0.9733592718,0.2041094999
H,0,1.4066532289,-1.8488907963,-0.1504455268
O,0,0.6470974126,1.398026098,1.2340261732
H,0,1.1741098926,0.6970821446,1.6321801842
O,0,-2.048948783,0.7944556856,1.4830198609
H,0,-1.4455822586,1.2887436498,2.0446722076

Erythrose, acyclic, conformation 12
H CBS-QB3 -457.45749 H_{rel} = +1.94 kcal/mol
H CBS-APNO -457.97784 H_{rel} = +1.95 kcal/mol
C,0,0.1612526707,2.1125587357,-0.3060475324
H,0,-0.1833329603,2.6098529205,0.6155101238
O,0,0.1682843045,2.6686513089,-1.3832859085

C,0,0.6193212067,0.6673965344,-0.1740111831
H,0,1.4701016452,0.6433834792,0.5250577715
C,0,-0.494943858,-0.1898569553,0.440838242
H,0,-1.4215616243,-0.0444597159,-0.1369837831
C,0,-0.15304816,-1.6843005793,0.4082828821
H,0,0.8630639624,-1.8265446748,0.8104835843
H,0,-0.8510261705,-2.2125057888,1.0700500299
O,0,1.0050549345,0.1678321354,-1.4350621707
H,0,0.874996598,0.9011768713,-2.049774074
O,0,-0.624362606,0.2932602481,1.7721075877
H,0,-1.362302244,-0.1676021227,2.1747521705
O,0,-0.3077933183,-2.2420019488,-0.8773786917
H,0,0.2342956192,-1.6978404479,-1.4555390483

Erythrose, acyclic, conformation 13
H CBS-QB3 -457.45707 H_{rel} = +2.20 kcal/mol
H CBS-APNO -457.97758 H_{rel} = +2.11 kcal/mol
C,0,-1.580836801,0.7584829493,0.4374566239
H,0,-2.3586029461,0.2284970148,-0.1402684877
O,0,-1.6488736076,1.9437488006,0.6732939622
C,0,-0.4423606824,-0.0830197357,0.9971431475
H,0,-0.8912687776,-0.804222656,1.7002905852
C,0,0.329813179,-0.8919800475,-0.0560576616
H,0,-0.3369580543,-1.6480697303,-0.4928495434
C,0,0.8734236758,-0.0022504231,-1.1752163483
H,0,1.4281779779,0.833808648,-0.7395369286
H,0,1.5616576225,-0.5931238083,-1.7925019015
O,0,0.4754484199,0.7402644023,1.6820777409
H,0,0.0968610059,1.6273170874,1.6427648953
O,0,1.3788391508,-1.5959874838,0.5799820296
H,0,1.8178326703,-0.9464838711,1.1376563464
O,0,-0.1849093691,0.5631216609,-1.9413171736
H,0,-0.5182434642,-0.1301028075,-2.5139172864

Erythrose, acyclic, conformation 14
H CBS-QB3 -457.45631 H_{rel} = +2.68 kcal/mol
H CBS-APNO -457.97653 H_{rel} = +2.77 kcal/mol
C,0,0.5492774034,-2.0979822933,-0.472696271
H,0,0.5704534329,-2.9833466213,0.1917293242
O,0,0.5380168832,-2.2079479271,-1.6805647702
C,0,0.5269567138,-0.7680464154,0.2729643598
H,0,1.5763292313,-0.4369427446,0.3763835082
C,0,-0.2209019695,0.319950979,-0.5184459032

H,0,-1.2617374836,-0.0207761366,-0.6606314549
C,0,-0.2297824275,1.6609458226,0.2176994468
H,0,0.801787102,1.9616795993,0.4251016714
H,0,-0.6824948448,2.4159609078,-0.4365779052
O,0,-0.0616837279,-1.0604405133,1.5237874599
H,0,-0.2893914727,-0.2129825829,1.9203826085
O,0,0.4179226831,0.5786299875,-1.7505974969
H,0,0.4918370092,-0.2716069884,-2.1953228058
O,0,-0.8928608096,1.595000188,1.4811390588
H,0,-1.8337277234,1.5279047386,1.3056491696

Erythrose, acyclic, conformation 15
H CBS-QB3 -457.45625 H_{rel} = +2.72 kcal/mol
H CBS-APNO -457.97652 H_{rel} = +2.77 kcal/mol
C,0,1.0888224626,0.566442175,1.8921349566
H,0,2.0759293647,0.2253141882,2.2595572397
O,0,0.5360613085,1.5376945373,2.3636734364
C,0,0.5324547873,-0.2875379584,0.756630417
H,0,-0.0962576268,-1.0714818311,1.2177452669
C,0,-0.3692413258,0.5304201972,-0.1862974737
H,0,0.2466760226,1.3178628608,-0.6491564667
C,0,-0.9844718266,-0.3256262223,-1.2887537487
H,0,-1.5891875215,-1.1134272475,-0.8176439303
H,0,-1.6418328806,0.3136680915,-1.8909436255
O,0,1.6732929624,-0.828823248,0.1235443523
H,0,1.3855539366,-1.1228600906,-0.7469210117
O,0,-1.4764272392,1.0636519967,0.5112172536
H,0,-1.1099257818,1.5594077475,1.2500467174
O,0,0.0648320969,-0.8824101521,-2.0823029551
H,0,-0.3332787393,-1.4812950442,-2.7145304282

Erythrose, acyclic, conformation 16
H CBS-QB3 -457.45579 H_{rel} = +3.01 kcal/mol
H CBS-APNO -457.97642 H_{rel} = +2.84 kcal/mol
C,0,-2.0864666381,0.1000014784,-0.4030156935
H,0,-2.6099891249,-0.3605053077,0.4512283963
O,0,-2.6014847118,0.226364747,-1.4927706623
C,0,-0.6550503886,0.5573435833,-0.1615110103
H,0,-0.6811272792,1.3470384355,0.6096667533
C,0,0.1831596435,-0.609306849,0.396115798
H,0,0.0474873731,-1.4927744332,-0.2377573518
C,0,1.6806123908,-0.2861973535,0.4393979677
H,0,1.8203348626,0.6745592177,0.969827179

H,0,2.1710972601,-1.0696037669,1.0260206333
O,0,-0.1082179428,1.0711798402,-1.3551043626
H,0,-0.8182535904,1.012645142,-2.0073886241
O,0,-0.3163282245,-0.9566271341,1.6811794914
H,0,-0.0480279419,-0.2592397438,2.2850380922
O,0,2.2784503695,-0.2755477379,-0.835733489
H,0,1.7438039425,0.3226698821,-1.3651931176

Erythrose, acyclic, conformation 17
H CBS-QB3 -457.45559 H_{rel} = +3.13 kcal/mol
H CBS-APNO -457.97620 H_{rel} = +2.97 kcal/mol
C,0,1.4654564103,1.0724391781,0.9085690816
H,0,2.4279617829,0.6565325769,1.2734243843
O,0,1.3768004974,2.1861498106,0.44651788
C,0,0.2772770046,0.1289523883,1.0781066275
H,0,-0.1474194496,0.343800767,2.069207265
C,0,-0.8294304478,0.3417441011,0.0349825914
H,0,-1.2239463852,1.3570593195,0.1497361673
C,0,-0.3516053149,0.165967998,-1.4077265541
H,0,-1.186158245,0.3905895556,-2.0841707942
H,0,0.4735800794,0.8441809767,-1.636957011
O,0,0.69247661,-1.2279631082,1.1265870214
H,0,0.9006852379,-1.4685946999,0.2155507013
O,0,-1.8839855253,-0.5806081639,0.2903601179
H,0,-1.4748700471,-1.3224415675,0.7452154782
O,0,0.1295651909,-1.1625903914,-1.6306480534
H,0,-0.6463873984,-1.726218741,-1.5777549033

Erythrose, α-furanose, global minimum CBS APNO
H CBS-QB3 -457.46597
H CBS-APNO -457.98643
O,0,0.0298717544,-0.0667134586,-1.5921830785
C,0,0.0350516236,1.0747481984,-0.7638992314
C,0,-0.3642909559,0.6176499945,0.6589759786
C,0,-0.907727153,-0.7998808887,0.4056766915
C,0,-0.0145226935,-1.2318009736,-0.762236039
H,0,-0.6740945807,1.791343475,-1.1930777511
H,0,-1.0970531167,1.2901001058,1.1182765884
H,0,-1.9491022519,-0.7555430576,0.0681364333
H,0,-0.4219243911,-2.052694299,-1.3553154188
H,0,0.9858889083,-1.4989129718,-0.3948511388
O,0,1.3228706665,1.6499610783,-0.6929489791
H,0,1.691788643,1.5885995614,-1.5766209777

O,0,0.7619536619,0.478418699,1.5075524665
H,0,1.4843233005,0.9357276759,1.0651829619
O,0,-0.8524614774,-1.6304712162,1.5334668723
H,0,-0.0305719379,-1.3905319227,1.973865622

Erythrose, α-furanose, global minimum CBS-QB3
H CBS-QB3 -457.46618 H_{rel} = -0.13 kcal/mol
H CBS-APNO -457.98639 H_{rel} = +0.02 kcal/mol
O,0,-0.0355675125,-0.0988839018,-1.7266198223
C,0,0.2202376256,0.9568492206,-0.8588995086
C,0,-0.650495377,0.6861186399,0.3780206075
C,0,-0.4874211231,-0.841709725,0.5019313358
C,0,-0.3946720736,-1.2699626141,-0.9676967039
H,0,-0.0088416122,1.8899620362,-1.3841964961
H,0,-1.6922233163,0.9437279163,0.1530953278
H,0,-1.3217140582,-1.3167587042,1.0233794497
H,0,-1.3577599347,-1.6300463457,-1.3460408968
H,0,0.3612049666,-2.0513981566,-1.0995739596
O,0,1.5919618818,0.9273644709,-0.4810281432
H,0,1.6720608374,1.597085809,0.2066047222
O,0,-0.2197948458,1.3920500684,1.5190198568
H,0,0.2356882106,0.7366589397,2.058745657
O,0,0.6750903939,-1.1476306122,1.2603168122
H,0,1.4122459376,-0.7734280415,0.7629417616

Erythrose, β-furanose, global minimum
H CBS-QB3 -457.46503
H CBS-APNO -457.98524
O,0,-0.186252559,1.0430598968,-0.9832528724
C,0,-1.0754912782,0.0012176979,-0.5978056555
C,0,-0.3615298225,-0.805517296,0.5169775202
C,0,1.0082457938,-0.0931010738,0.6151763727
C,0,1.1287586156,0.5082922977,-0.777637008
H,0,-1.9933086562,0.4782812053,-0.2391649352
H,0,-0.2388912293,-1.8326545682,0.1578873963
H,0,1.8246173708,-0.7599886932,0.8998670755
H,0,1.3602494813,-0.2577852323,-1.5290384605
H,0,1.8481415377,1.3277249126,-0.837700818
O,0,-1.3161823507,-0.8730592244,-1.6680580874
H,0,-1.6897768938,-0.3419208812,-2.3750626984
O,0,-1.053292605,-0.8320112637,1.740650739
H,0,-0.6416722597,-0.1449150003,2.2747782107
O,0,0.9482977866,0.9348843311,1.6003330702

H,0,0.4380860685,1.6474938919,1.202050151

Erythrose, β-furanose, conformation 2
H CBS-QB3 -457.46502 H_{rel} = +0.01 kcal/mol
H CBS-APNO -457.98523 H_{rel} = +0.00 kcal/mol
O,0,0.1229088498,-0.9171204885,-1.1680874937
C,0,1.1937648381,-0.3187719094,-0.4672622384
C,0,0.5594922574,0.0937772183,0.8509133638
C,0,-0.7467486594,0.7399580434,0.3492402322
C,0,-1.0469190503,-0.1035904668,-0.9201519184
H,0,1.9742171904,-1.0810264872,-0.3680599489
H,0,1.1800253623,0.7551657126,1.4562809719
H,0,-0.564382134,1.7819944907,0.0773517333
H,0,-1.252861403,0.5227782193,-1.7940259686
H,0,-1.8938257853,-0.7736654472,-0.746482529
O,0,1.659423907,0.8456779531,-1.0945316859
H,0,1.9285011053,0.5916429415,-1.9802073967
O,0,0.2610022867,-1.055907419,1.6336472322
H,0,-0.002257804,-1.7416847426,1.0115787541
O,0,-1.7741317679,0.7202066352,1.3117041317
H,0,-1.5982081931,-0.0594342532,1.8480927606

Erythrose, β-furanose, conformation 3
H CBS-QB3 -457.46434 H_{rel} = +0.43 kcal/mol
H CBS-APNO -457.98427 H_{rel} = +0.61 kcal/mol
O,0,0.0624484715,-0.837437742,-1.4141650564
C,0,1.1852154124,-0.4158489909,-0.6731191537
C,0,0.6670859459,-0.0674404496,0.7132781751
C,0,-0.6714190913,0.5899906679,0.3414859045
C,0,-1.1229943146,-0.2712658022,-0.8459378025
H,0,1.905783697,-1.2396600171,-0.6760747682
H,0,1.3522443312,0.6059188744,1.2435832874
H,0,-0.5043001786,1.6271937197,0.032815746
H,0,-1.6573275198,0.3079019839,-1.6056083877
H,0,-1.7684883087,-1.0780689013,-0.4817755335
O,0,1.7353579783,0.770140102,-1.201907494
H,0,1.8902457995,0.5979365823,-2.1329966235
O,0,0.4394804448,-1.2610287671,1.4283962622
H,0,-0.3573737609,-1.1018818526,1.9433580129
O,0,-1.6345285961,0.5021450795,1.3839221358
H,0,-1.5214273107,1.2714065131,1.9447452956

Erythrose, β-furanose, conformation 4

H CBS-QB3 -457.46403 H_{rel} = +0.63 kcal/mol
H CBS-APNO -457.98411 H_{rel} = +0.70 kcal/mol
O,0,-0.1109114553,-0.6613590283,-1.4352163096
C,0,1.0475520794,-0.2153911306,-0.773252326
C,0,0.6326683684,0.0610949076,0.6672050945
C,0,-0.7647986376,0.653298058,0.4503624426
C,0,-1.2736386417,-0.2100328578,-0.7215620774
H,0,1.8041550967,-1.0035348466,-0.8761203609
H,0,1.3213818898,0.7416854893,1.1782442525
H,0,-0.6664236488,1.6969076508,0.1434073987
H,0,-1.92591302,0.3442808279,-1.4031929506
H,0,-1.8099596313,-1.0831639653,-0.3324713554
O,0,1.5076101983,1.0094903776,-1.29659611
H,0,1.5169615548,0.9012301245,-2.250110958
O,0,0.4306409469,-1.1442560036,1.3934394585
H,0,1.2864926464,-1.4777438296,1.6640539474
O,0,-1.5844684104,0.6202426144,1.5913082792
H,0,-1.4113493353,-0.232749388,2.0005015744

Erythrose, β-furanose, conformation 5
H CBS-QB3 -457.46304 H_{rel} = +1.25 kcal/mol
H CBS-APNO -457.98314 H_{rel} = +1.32 kcal/mol
O,0,-0.079545065,-0.7463847375,-1.42525629
C,0,0.9251593137,-0.1448426495,-0.6359589338
C,0,0.1923314271,0.7378090825,0.3702851398
C,0,-1.0687145022,-0.0765710068,0.6272512568
C,0,-1.3529920894,-0.6037070498,-0.7765776557
H,0,1.4978750187,-0.9274337892,-0.1170121789
H,0,-0.0608539246,1.6799906502,-0.1375113323
H,0,-1.8952736328,0.5284512516,1.0238379867
H,0,-1.9740222918,0.1029737903,-1.3428119247
H,0,-1.8472945644,-1.5781877126,-0.7338643357
O,0,1.7726461274,0.6622167629,-1.4026640436
H,0,2.2394087627,0.0839805414,-2.0091276892
O,0,0.8946367268,0.9590914764,1.5751103251
H,0,1.7533516041,1.3147374471,1.3368608531
O,0,-0.7766677739,-1.1728757697,1.4738295336
H,0,-0.2200451361,-0.8192502872,2.1736082891

Threose, acyclic, global minimum
H CBS-QB3 -457.46171
H CBS-APNO -457.98216
C,0,-2.0703791928,-0.0911190135,-0.532895425

H,0,-2.3770216111,0.7855599335,-1.1324372822
O,0,-2.6528686602,-1.1495990978,-0.595079753
C,0,-0.8760052358,0.0968562219,0.3922862174
C,0,0.3125552279,0.6478366801,-0.4093844037
H,0,0.0778769028,1.6853815733,-0.6848594225
C,0,1.6162909765,0.6186489289,0.3993260472
H,0,2.3753206886,1.2058222401,-0.1257088667
H,0,1.456124847,1.0565690885,1.3957137079
O,0,0.4768375673,-0.0810847927,-1.6107273839
H,0,1.07295497,-0.8018547296,-1.3809131171
O,0,2.119871354,-0.7054013754,0.476601473
H,0,1.435621215,-1.2119225843,0.9265272937
H,0,-1.1616107307,0.832840831,1.1634206061
O,0,-0.5564673897,-1.1383057131,1.0020569928
H,0,-1.2471009287,-1.749228191,0.715073316

Threose, acyclic, conformation 2
H CBS-QB3 -457.45977 H_{rel} = +1.22 kcal/mol
H CBS-APNO -457.98018 H_{rel} = +1.24 kcal/mol
C,0,0.1299986096,-2.1626588719,-0.294318208
H,0,-0.862780385,-2.4737249171,-0.6710716871
O,0,1.1286913777,-2.8136159066,-0.4989713049
C,0,0.1776539312,-0.874491977,0.5173900682
C,0,-0.4706389838,0.269115543,-0.2631582163
H,0,-1.473816109,-0.0252610986,-0.5955993688
C,0,-0.5861726811,1.5548344534,0.5518467499
H,0,-1.2405679622,1.3973101698,1.4166962397
H,0,0.4128838138,1.8362251538,0.9181924293
O,0,0.2802298848,0.5130935832,-1.4468429871
H,0,1.1957299322,0.5586212989,-1.1504532721
O,0,-1.1652089043,2.5776396375,-0.2328330826
H,0,-0.6882390052,2.5495825863,-1.0665507626
H,0,-0.3869416384,-1.0524042486,1.4488659096
O,0,1.5183851874,-0.5323470929,0.7904293325
H,0,2.0287929322,-1.3229183132,0.5763781604

Threose, acyclic, conformation 3
H CBS-QB3 -457.45908 H_{rel} = +1.65 kcal/mol
H CBS-APNO -457.97941 H_{rel} = +1.73 kcal/mol
C,0,-1.8249223413,-0.4044951023,1.1056604596
H,0,-1.7036072625,0.0763397241,2.0950530375
O,0,-2.9079295851,-0.7523666141,0.6913196704
C,0,-0.5564469835,-0.6246167007,0.29841499

C,0,0.2062687466,0.693194634,0.1342654597
H,0,0.3632423033,1.1490734885,1.1194430643
C,0,1.5683068835,0.4663514727,-0.5141588889
H,0,1.4195228128,-0.0269328256,-1.4854660938
H,0,2.0371126818,1.4461029105,-0.6780625462
O,0,-0.5713854095,1.5985311833,-0.624351254
H,0,-0.8696863341,1.0883541121,-1.3839864596
O,0,2.3249143533,-0.3428385823,0.375859476
H,0,3.1461313723,-0.5629402037,-0.0639297022
H,0,0.0755536149,-1.3406798563,0.844331945
O,0,-0.8874981661,-1.11035869,-0.9865840096
H,0,-1.8195766863,-1.3527189504,-0.9288091483

Threose, acyclic, conformation 4
H CBS-QB3 -457.45834 H_{rel} = +2.11 kcal/mol
H CBS-APNO -457.97919 H_{rel} = +1.86 kcal/mol
C,0,1.2072822131,-1.204538569,-0.1217028322
H,0,0.5808152327,-2.1118405147,-0.1247494635
O,0,2.1846327104,-1.0947156295,-0.8306319899
C,0,0.8350026116,-0.1093218114,0.8686402027
C,0,-0.6316523007,0.3494540311,0.7527105175
H,0,-0.7697872322,1.150432905,1.4874679381
C,0,-0.9534541939,0.897326649,-0.6296420456
H,0,-1.9360851936,1.387181594,-0.6075446527
H,0,-0.1828096225,1.6179031475,-0.9252711777
O,0,-1.5109136836,-0.7081280175,1.0929979757
H,0,-1.7147286452,-1.1517836165,0.2651708155
O,0,-0.9779776057,-0.2384942012,-1.4972571491
H,0,-1.1197796466,0.0695415159,-2.3924033065
H,0,0.9455703702,-0.5534204491,1.8721574118
O,0,1.685113005,0.9983704153,0.706235892
H,0,2.361771981,0.7010325512,0.0858218637

Threose, acyclic, conformation 5
H CBS-QB3 -457.45735 H_{rel} = +2.74 kcal/mol
H CBS-APNO -457.97757 H_{rel} = +2.88 kcal/mol
C,0,-1.6230086341,1.270236385,-0.8029551481
H,0,-2.7153147597,1.3188519054,-0.62748061
O,0,-1.1127354111,1.7705995317,-1.781812043
C,0,-0.8499380226,0.5029283834,0.2641162614
C,0,0.6148850337,0.291345158,-0.1440471049
H,0,1.0804378962,1.2871532266,-0.2035005663
C,0,1.3846546108,-0.5485922226,0.8714018434

H,0,2.4513491804,-0.5387372731,0.6121472302
H,0,1.2613981267,-0.1431988878,1.8806776455
O,0,0.7405888515,-0.4026068041,-1.3744379256
H,0,0.3465956125,0.1602507493,-2.0448136614
O,0,0.8876082612,-1.8846356486,0.8993656629
H,0,0.9575481491,-2.1945896699,-0.0090860577
H,0,-0.8499189379,1.1266373417,1.1789406498
O,0,-1.5948353982,-0.6822544202,0.4627105286
H,0,-0.9803145585,-1.333387755,0.8187732951

Threose, acyclic, conformation 6
H CBS-QB3 -457.45656 H_{rel} = +3.23 kcal/mol
H CBS-APNO -457.97698 H_{rel} = +3.25 kcal/mol
C,0,-1.624558776,1.4035488657,-0.7567204298
H,0,-2.5978115869,0.9852914148,-1.0837526973
O,0,-1.2268754568,2.4869326325,-1.1094637999
C,0,-0.832742729,0.506599877,0.1928285369
C,0,0.5950300716,0.2822257396,-0.3255427846
H,0,1.0020264363,1.2534778797,-0.6303967073
C,0,1.5007916056,-0.3381706648,0.7362830247
H,0,2.5231090503,-0.4121442223,0.3431447979
H,0,1.5163602471,0.2800657275,1.6400188367
O,0,0.5536246943,-0.5629409054,-1.4689274306
H,0,-0.2439813574,-1.0911340704,-1.3653157404
O,0,1.0200265372,-1.6256902457,1.1290661028
H,0,1.174818996,-2.1961523261,0.3721767034
H,0,-0.7906591674,1.0218248866,1.1650056722
O,0,-1.5414676283,-0.7204236021,0.28217259
H,0,-1.0276909365,-1.2743109864,0.8804233252

Threose, α-furanose, global minimum
H CBS-QB3 -457.46352
H CBS-APNO -457.98347
O,0,-0.191179408,0.8560229999,-1.2939010433
C,0,-1.1113485981,-0.0524717464,-0.7082752892
C,0,-0.2609373309,-0.9515955632,0.2097741622
C,0,0.7937748296,0.0382526376,0.6886608513
C,0,1.0602321974,0.8034988847,-0.6071594875
H,0,-1.594708848,-0.6168246423,-1.5123272032
H,0,-0.8533245811,-1.3645656726,1.0339480017
H,0,1.6812107687,-0.4646085094,1.0892444391
H,0,1.7989350248,0.2691388521,-1.2159501427
H,0,1.4103602529,1.8266585997,-0.4238091485

O,0,-2.1342377445,0.6029285425,-0.0233339601
H,0,-1.7079902236,1.0354072122,0.7236338676
O,0,0.4362544613,-1.9480392785,-0.5169057784
H,0,-0.2190428353,-2.4874787954,-0.9625060738
O,0,0.1574046104,0.8540336393,1.6724245136
H,0,0.7345984245,1.5996428399,1.8464812911

Threose, α-furanose, conformation 2
H CBS-QB3 -457.46345 H_{rel} = +0.04 kcal/mol
H CBS-APNO -457.98332 H_{rel} = +0.09 kcal/mol
O,0,-0.137456088,0.8482821467,-1.3726743042
C,0,-1.0742828324,-0.0020342776,-0.7457854934
C,0,-0.2346680565,0.9047224127,0.1753889898
C,0,0.8257044697,0.0855259921,0.6564701129
C,0,1.0835684443,0.8796838352,-0.6221284941
H,0,-1.5988108569,-0.5645100302,-1.5251387727
H,0,-0.8417478879,-1.3208360265,0.9902745179
H,0,1.7230517929,-0.4207129761,1.0296051658
H,0,1.8781854644,0.4112719356,-1.2117672027
H,0,1.3468141915,1.915962552,-0.389471189
O,0,-2.0582376613,0.7047403638,-0.0483685092
H,0,-1.5876162814,1.2011241327,0.6290390421
O,0,0.46077727,-1.9067723969,-0.5435546967
H,0,-0.182962936,-2.3834515959,-1.0703874732
O,0,0.2804065957,0.9822080133,1.6253350901
H,0,0.1172743717,0.4742397443,2.4231622164

Threose, β-furanose, global minimum
H CBS-QB3 -457.46499
H CBS-APNO -457.98521
O,0,0.0145597169,-0.7924880839,-1.5027076515
C,0,1.0237362945,-0.0826685013,-0.8383676526
C,0,0.6586802997,-0.1530006255,0.6413086383
C,0,-0.8613732721,0.0154529122,0.5626456659
C,0,-1.1993459425,-0.7438059782,-0.7223957565
H,0,1.980951684,-0.5428869977,-1.103262321
H,0,0.8769622309,-1.154262644,1.0282469609
H,0,-1.0855748947,1.0857990343,0.4506268546
H,0,-1.9888207076,-0.2676304349,-1.3114402101
H,0,-1.4971061912,-1.7672725429,-0.4706407068
O,0,1.020333918,1.2945613813,-1.1629378956
H,0,1.2025105013,1.3664659844,-2.1015840678
O,0,1.3148928111,0.7758347285,1.4695572534

H,0,1.3365637181,1.595567619,0.967145032
O,0,-1.5609144119,-0.5416866092,1.6562606816
H,0,-1.2360557547,-0.087978242,2.4375461751

Threose, β-furanose, conformation 2
H CBS-QB3 -457.46439 H_{rel} = +0.38 kcal/mol
H CBS-APNO -457.98443 H_{rel} = +0.49 kcal/mol
O,0,0.2108729381,-0.8556302844,1.1034816525
C,0,1.0794315947,-0.4127178507,0.0780113287
C,0,0.2860578959,0.6202441326,-0.7708231823
C,0,-0.9095699992,0.918347988,0.1444710735
C,0,-1.129314571,-0.4638111607,0.748045482
H,0,1.9573356453,0.0241495922,0.5683398294
H,0,0.8781139723,1.5208226801,-0.9711484792
H,0,-1.7584333849,1.2972272872,-0.4273772341
H,0,-1.558156157,-1.1569747426,0.015920592
H,0,-1.7246403448,-0.4503079722,1.6631475215
O,0,1.4694684548,-1.4686267423,-0.7665973232
H,0,1.6906605128,-2.2084508014,-0.197758876
O,0,-0.2117683006,0.0511291302,-1.9619701651
H,0,0.3301198136,-0.7237107373,-2.1358613174
O,0,-0.5963161362,1.8737972446,1.1427084257
H,0,-0.0138609339,1.4345102366,1.7674106718

Threose, β-furanose, conformation 3
H CBS-QB3 -457.46391 H_{rel} = +0.68 kcal/mol
H CBS-APNO -457.98376 H_{rel} = +0.91 kcal/mol
O,0,0.1637920996,-0.858715952,-1.214061113
C,0,1.0829954998,-0.0756147022,-0.4854772422
C,0,0.2718256154,0.679047069,0.5842075096
C,0,-0.8861865603,-0.2915177299,0.8137392473
C,0,-1.1339075799,-0.7817007121,-0.6072922314
H,0,1.8338118479,-0.7400487395,-0.0414331115
H,0,0.8628094148,0.8601206309,1.4936185528
H,0,-1.7595438848,0.2159017021,1.2416093285
H,0,-1.7638042203,-0.0704426383,-1.1528797724
H,0,-1.581844877,-1.7782535809,-0.6175998563
O,0,1.7014275765,0.872421344,-1.3246547051
H,0,1.8321363321,0.4344130354,-2.1680793035
O,0,-0.2576159519,1.8764931929,0.0630114961
H,0,0.3516276782,2.1383561017,-0.6351113563
O,0,-0.4727237804,-1.4062862428,1.5900084312
H,0,-0.2448002098,-1.0741717779,2.4603921261

Threose, β-furanose, conformation 4
H CBS-QB3 -457.46221 H_{rel} = +1.74 kcal/mol
H CBS-APNO -457.98253 H_{rel} = +1.68 kcal/mol
O,0,0.479550346,-1.0996427313,-0.956519723
C,0,1.1682795496,0.0355501298,-0.4362418684
C,0,0.192567467,0.6784219781,0.6012487561
C,0,-0.8743244708,-0.4054158348,0.7737516156
C,0,-0.9126911781,-0.9633780685,-0.6441654572
H,0,2.0938165412,-0.3289700057,0.0153212101
H,0,0.6832497737,0.9089419519,1.552221977
H,0,-1.819517194,0.0080684096,1.1304510498
H,0,-1.4132382911,-0.2711931105,-1.334282172
H,0,-1.3703770922,-1.9529976238,-0.7070544905
O,0,1.5513173179,0.9356745454,-1.4264366653
H,0,0.7435692165,1.376183474,-1.7067414891
O,0,-0.4448129208,1.8207076313,0.041116118
H,0,0.1543185264,2.5612146551,0.1504489276
O,0,-0.4542918978,-1.3940561712,1.6971781625
H,0,0.2225853065,-1.9091082296,1.2497030489

Polyols
Ethylene glycol, global minimum, Cramer Truhlar conformation 1, tG+g-
H CBS-QB3 -229.90557
H CBS-APNO -230.16667
C,0,0.0015915346,0.0021202327,0.003260895
C,0,-0.0003295883,0.0033392075,1.5218603109
O,0,1.3692859925,0.0258060412,1.9199646629
O,0,0.6613286332,-1.1450442772,-0.4966894104
H,0,1.4078711158,-0.2329739569,2.840322093
H,0,1.5007553358,-1.1635484033,-0.0307006498
H,0,-0.5423467482,0.8808178747,1.9047028795
H,0,-0.4950744173,-0.9114680594,1.876662023
H,0,-1.0262539161,-0.0232705998,-0.3728401476
H,0,0.4805074042,0.9273841399,-0.3527629128

Ethylene glycol, Cramer Truhlar conformation 2, g+G+g-
H CBS-QB3 -229.90480 H_{rel} = +0.48 kcal/mol
H CBS-APNO -230.16606 H_{rel} = +0.38 kcal/mol
C,0,0.0033961221,0.0014856606,0.0047871235
C,0,-0.0004943907,-0.0010382358,1.5268523334
O,0,1.3375086066,0.0273050418,2.0214647401
O,0,0.7427518867,-1.0956342697,-0.4928368275

H,0,1.6879937176,0.8974398301,1.8205615411
H,0,1.5349882799,-1.1251141094,0.049794799
H,0,-0.5944598309,0.836919643,1.9201588331
H,0,-0.4298000206,-0.9394564014,1.8889054226
H,0,-1.0177981984,-0.086929278,-0.3816011639
H,0,0.420487988,0.9588195058,-0.3549897091

Ethylene glycol, Cramer Truhlar conformation 3, g-G+g-
H CBS-QB3-229.90499 H_{rel} = +0.36 kcal/mol
H CBS-APNO -230.16528 H_{rel} = +0.87 kcal/mol
C,0,0.0006441596,0.0028688036,-0.0003110651
C,0,-0.0020096374,-0.002146237,1.5236491135
O,0,1.3269018999,0.0057457824,2.032075089
O,0,0.7416019324,-1.1003309684,-0.5087370405
H,0,1.6481059665,-0.8920353926,1.9397161927
H,0,1.6645687393,-0.8609246952,-0.4163781441
H,0,-0.4959048291,0.8987095751,1.9038249175
H,0,-0.5544032706,-0.8805655062,1.8866182949
H,0,0.4162231493,0.9536859478,-0.3632802465
H,0,-1.0219996881,-0.0954837012,-0.3804868691

Ethylene glycol, Cramer Truhlar conformation 4, tTg+
H CBS-QB3 -229.90121 H_{rel} = +2.74 kcal/mol
H CBS-APNO -230.16247 H_{rel} = +2.64 kcal/mol
C,0,0.0065439992,0.0006098009,-0.0027819131
C,0,-0.0014370058,-0.0073927106,1.520457065
O,0,1.3505518758,-0.0129211619,1.9501316765
O,0,-1.3114480055,0.0233965941,-0.5278393792
H,0,1.3470399365,0.0736581063,2.9031670418
H,0,-1.7172903606,-0.8130828353,-0.2955785064
H,0,-0.5459625775,0.8796481065,1.8742011403
H,0,-0.5393217695,-0.9026892645,1.875579688
H,0,0.5007016106,0.9091172646,-0.3571876573
H,0,0.5800538612,-0.8641824512,-0.3660163949

Ethylene glycol, Cramer Truhlar conformation 5, g+Tg-
H CBS-QB3 -229.90110 H_{rel} = +2.80 kcal/mol
H CBS-APNO -230.16246 H_{rel} = +2.64 kcal/mol
C,0,0.0060360275,0.0020124934,0.0000334198
C,0,-0.0060360275,-0.0020124934,1.5279426866
O,0,1.3059571105,0.0075559948,2.0663862191
O,0,-1.3059571105,-0.0075559948,-0.5384101127
H,0,1.7008155471,0.8447556705,1.8176720339

H,0,-1.7008155471,-0.8447556705,-0.2896959275
H,0,-0.6092820695,0.844821294,1.885690562
H,0,-0.4760158729,-0.923828363,1.8900967856
H,0,0.4760158729,0.923828363,-0.3621206792
H,0,0.6092820695,-0.844821294,-0.3577144556

Ethylene glycol, Cramer Truhlar conformation 6, g+G+g+
H CBS-QB3 -229.90083 H_{rel} = +2.97 kcal/mol
H CBS-APNO -230.16213 H_{rel} = +2.85 kcal/mol
C,0,0.0209098329,0.0077977151,-0.0001171543
C,0,0.020126518,0.0096409931,1.5250173189
O,0,1.3187950286,-0.0490037662,2.0836940421
O,0,0.8800575301,0.9834228187,-0.5587938775
H,0,1.788148293,-0.7493973931,1.6274902696
H,0,0.7016026145,1.8074359026,-0.102590105
H,0,-0.6170152934,-0.8178538301,1.877554923
H,0,-0.4199275493,0.9446532136,1.8939620449
H,0,0.3886041831,-0.9579643153,-0.3690618803
H,0,-1.0170309706,0.1234534913,-0.3526547584

Ethylene glycol, Cramer Truhlar conformation 7, tG+g+
H CBS-QB3 -229.90012 H_{rel} = +3.42 kcal/mol
H CBS-APNO -230.16133 H_{rel} = +3.35 kcal/mol
C,0,-0.0026557946,0.0062922996,-0.0029984694
C,0,-0.0023903957,-0.0021557989,1.5169273088
O,0,1.3380409756,-0.007639112,1.9727084006
O,0,0.5809716661,-1.1497348882,-0.5733765561
H,0,1.3098716288,-0.0304572313,2.9293551952
H,0,0.1247612744,-1.9045361013,-0.1983832342
H,0,-0.5481174069,0.8885784771,1.8683965103
H,0,-0.5493582668,-0.8932939224,1.87186225
H,0,-1.0404943216,0.1470979165,-0.3473344869
H,0,0.5928520341,0.8543699862,-0.3539576525

Ethylene glycol, Cramer Truhlar conformation 8, g+Tg+
H CBS-QB3 -229.90077 H_{rel} = +3.01 kcal/mol
H CBS-APNO -230.16220 H_{rel} = +2.80 kcal/mol
C,0,0.0063922137,0.0014943087,0.0000789511
C,0,-0.0064900995,0.0009858963,1.5276009094
O,0,1.3036693601,0.0067702965,2.0685297902
O,0,-1.3001480499,-0.0959931733,-0.5408499292
H,0,1.7213619469,0.8193090687,1.7783177121
H,0,-1.7805777794,0.6810998922,-0.2506378584

H,0,-0.4918263899,-0.9120465528,1.8825452899
H,0,-0.5974264427,0.8589541469,1.891319862
H,0,0.5278739122,0.9033657592,-0.3636400094
H,0,0.5621751742,-0.8704488514,-0.3548654213

Ethylene glycol, Cramer Truhlar conformation 9, tTt
H CBS-QB3 -229.90149 H_{rel} = +2.56 kcal/mol
H CBS-APNO -230.16263 H_{rel} = +2.54 kcal/mol
C,0,0.0020993315,0.,-0.000385347
C,0,-0.0020993315,0.,1.5176333669
O,0,1.3550635203,0.,1.935642937
O,0,-1.3550635203,0.,-0.4183949171
H,0,1.3547112988,0.,2.8924350938
H,0,-1.3547112988,0.,-1.3751870739
H,0,-0.5388878549,-0.8920720029,1.8710512832
H,0,-0.5388878549,0.8920720029,1.8710512832
H,0,0.5388878549,-0.8920720029,-0.3538032633
H,0,0.5388878549,0.8920720029,-0.3538032633

Ethylene glycol, Cramer Truhlar conformation 10, tG+t
H CBS-QB3-229.90090 H_{rel} = +2.93 kcal/mol
H CBS-APNO -230.16192 H_{rel} = +2.98 kcal/mol
C,0,-0.0334476571,0.0053876194,-0.0002524152
C,0,-0.015184396,0.0302854144,1.5142830862
O,0,1.3368176881,0.0600647533,1.9453985707
O,0,0.3442013718,-1.2931414158,-0.4313678997
H,0,1.3621703499,-0.3084257204,2.8273183456
H,0,0.703294325,-1.2066530485,-1.3132876746
H,0,-0.5616078707,0.9187674157,1.8678475076
H,0,-0.5359601405,-0.8681366733,1.8742073832
H,0,0.667090486,0.7719487284,-0.3601767122
H,0,-1.0450219052,0.2597425306,-0.3538168366

Glycerol, global minimum, Hadad conformation 100
H CBS-QB3 -344.27847
H CBS-APNO -344.66993
C,0,1.305352315,0.0368482861,0.6091446159
C,0,-0.0109680455,0.7973409058,0.4431173041
C,0,-1.2400435017,-0.0868237079,0.6805121489
O,0,1.4211841934,-1.002987739,-0.3613825071
O,0,-1.4385321483,-0.9800985753,-0.4080086285
O,0,-0.0622163761,1.3436546414,-0.8704484719
H,0,1.4554702816,-0.546487242,-1.206210106

H,0,-0.6615763555,0.7666020056,-1.3543114886
H,0,-0.6279031955,-1.49645358,-0.4653686324
H,0,1.3526663054,-0.4442255901,1.5920041596
H,0,2.1451277284,0.7397135707,0.522469641
H,0,-0.0275660907,1.626302112,1.164272613
H,0,-1.1388973272,-0.623490795,1.6351534155
H,0,-2.1378427834,0.5376817076,0.7279219365

Glycerol, conformation 2, Hadad conformation 95
H CBS-QB3 -344.27791 H_{rel} = +0.35 kcal/mol
H CBS-APNO -344.66938 H_{rel} = +0.35 kcal/mol
C,0,-0.9524381637,-0.7913455658,-0.0108273008
C,0,-0.0002692462,0.2294335121,0.5972058745
C,0,1.4453278015,-0.2536549099,0.5901824919
O,0,-2.2206108123,-0.1438507796,-0.1146423867
O,0,1.9067405697,-0.4562713023,-0.7333910608
O,0,-0.0238255393,1.4227367448,-0.1753775628
H,0,-2.7546680011,-0.6516262348,-0.7253392759
H,0,-0.9487436156,1.5534725613,-0.4021037219
H,0,1.6871205303,0.3604997362,-1.1901316417
H,0,-0.5722441714,-1.0819479948,-0.9972812792
H,0,-1.0108055026,-1.6801432696,0.6339107522
H,0,-0.3144471301,0.4350832771,1.6328437398
H,0,2.0650442616,0.4851314835,1.119133383
H,0,1.5272590193,-1.2123522584,1.1138619883

Glycerol, conformation 3, Hadad conformation 46
H CBS-QB3 -344.27713 H_{rel} = +0.84 kcal/mol
H CBS-APNO -344.66870 H_{rel} = +0.77 kcal/mol
C,0,0.9503151467,-0.798037927,-0.0596909638
C,0,0.0114906128,0.2160643102,0.5879328819
C,0,-1.438932297,-0.2534781827,0.6053778052
O,0,2.2369142684,-0.2160510613,-0.2680987763
O,0,-1.9248668015,-0.439085682,-0.7105683005
O,0,0.0351343971,1.4246947439,-0.1596649813
H,0,2.6447531044,-0.1221247971,0.5952807783
H,0,0.937987977,1.5056073131,-0.480952415
H,0,-1.7119143476,0.3813656963,-1.163771005
H,0,0.5693251505,-1.0608018989,-1.048564678
H,0,1.0127797586,-1.7097963353,0.5509906457
H,0,0.3443392029,0.4023770492,1.6245548724
H,0,-1.5191247944,-1.2168859931,1.1210105618
H,0,-2.042301378,0.4848337646,1.1538295745

Glycerol, conformation 4, Hadad conformation 101
H CBS-QB3 -344.27728 H_{rel} = +0.75 kcal/mol
H CBS-APNO -344.66868 H_{rel} = +0.78 kcal/mol
C,0,1.3454649436,0.2386460602,0.4461645916
C,0,-0.0730660572,0.8044533616,0.4311827638
C,0,-1.135243323,-0.2610611298,0.7363506018
O,0,1.3876783621,-0.9122565794,-0.4010166511
O,0,-1.3468250882,-1.1045552707,-0.3867797686
O,0,-0.3669502094,1.4320915918,-0.8014954619
H,0,2.3011572482,-1.1842476115,-0.4855713694
H,0,-0.6945937105,0.7236714491,-1.3639489538
H,0,-0.4974447099,-1.5232978162,-0.5543172892
H,0,2.0289210386,1.0158431222,0.080721643
H,0,1.6206117104,-0.0321513729,1.4774547603
H,0,-0.1168304281,1.5806812147,1.2069161914
H,0,-0.8504593664,-0.8370292819,1.6300109371
H,0,-2.09171541,0.2333382629,0.9292630052

Glycerol, conformation 5, Hadad conformation 109
H CBS-QB3 -344.27683 H_{rel} = +1.03 kcal/mol
H CBS-APNO -344.66817 H_{rel} = +1.10 kcal/mol
C,0,1.2853625569,-0.7087760834,0.192112593
C,0,0.0151425382,-0.0104409533,-0.2681250808
C,0,-1.2543382835,-0.7151099116,0.1790477023
O,0,2.4315006351,-0.0001770656,-0.2357203263
O,0,-2.3362203957,0.0999237569,-0.2662057141
O,0,0.0633139075,1.2970202879,0.2902520934
H,0,2.2663180109,0.9116981851,0.0171195635
H,0,-0.7894152915,1.6918629333,0.0928807263
H,0,-3.1260425592,-0.1920706883,0.1888354552
H,0,1.3445632339,-1.7119857141,-0.2437803377
H,0,1.2602855693,-0.803034764,1.2896988858
H,0,0.0214656154,0.0484190791,-1.3674230561
H,0,-1.249400007,-0.795661909,1.2755191487
H,0,-1.3057635302,-1.722101153,-0.2603896531

Glycerol, conformation 6, Hadad conformation 48
H CBS-QB3 -344.27637 H_{rel} = +1.32 kcal/mol
H CBS-APNO -344.66770 H_{rel} = +1.40 kcal/mol
C,0,0.9918808196,-0.7881876329,-0.1080894146
C,0,0.0446269795,0.1936136219,0.5802116757
C,0,-1.3963557798,-0.2935478784,0.624742706

O,0,2.3013897428,-0.2542896283,-0.1473175264
O,0,-1.8464041058,-0.3528508355,-0.7284521309
O,0,0.1109081489,1.4543938903,-0.0813958079
H,0,2.1813156032,0.654601898,-0.4356879731
H,0,-0.4353197642,1.3462211358,-0.8662953102
H,0,-2.8014825823,-0.4102422167,-0.7156134438
H,0,0.6136760186,-1.0084464743,-1.1175272762
H,0,1.0393903686,-1.727220662,0.4554120794
H,0,0.4049119811,0.3666926906,1.6006467929
H,0,-1.4501513301,-1.2803934698,1.1072312356
H,0,-1.9935631002,0.4240105611,1.2029533936

Glycerol, conformation 7, Hadad conformation 7
H CBS-QB3 -344.27617 H_{rel} = +1.44 kcal/mol
H CBS-APNO -344.66762 H_{rel} = +1.45 kcal/mol
C,0,-1.2576038101,-0.718923569,0.2094337949
C,0,0.009181814,-0.0152770035,-0.2591683381
C,0,1.2901211372,-0.7032896234,0.1871419403
O,0,-2.4044963511,0.06074505,-0.1244486434
O,0,2.4273772917,0.0100437893,-0.2565966877
O,0,0.0541481666,1.2974837984,0.283584217
H,0,-2.5049665599,0.0112245364,-1.0773432244
H,0,-0.838843984,1.6424849444,0.2033831003
H,0,2.272622761,0.9184426464,0.0143229395
H,0,-1.25358658,-0.7898366877,1.301409865
H,0,-1.320871826,-1.7318624027,-0.2133950228
H,0,0.007354458,0.0362965587,-1.3620867156
H,0,1.3516086063,-1.7079973525,-0.245241582
H,0,1.2760228763,-0.7929276848,1.2849993569

Glycerol, conformation 8, Hadad conformation 43
H CBS-QB3 -344.27549 H_{rel} = +1.87 kcal/mol
H CBS-APNO -344.66713 H_{rel} = +1.76 kcal/mol
C,0,-1.4077385422,-0.2806397588,0.6237735288
C,0,0.0400558827,0.2025937853,0.5862724616
C,0,0.999631964,-0.7891745532,-0.0711742878
O,0,-1.9691003333,-0.290596409,-0.6890932202
O,0,2.2963672116,-0.2388653609,-0.1565591672
O,0,0.111116827,1.444857086,-0.1070157813
H,0,-1.6477959295,-1.0762890486,-1.1334995109
H,0,-0.4993600562,1.3500141404,-0.8456235935
H,0,2.1525950225,0.665735536,-0.4496507813
H,0,-2.0084045688,0.4287849058,1.1992174549

H,0,-1.476904478,-1.2686337947,1.1006746634
H,0,0.3917046204,0.3969602971,1.6063349725
H,0,0.6190743144,-1.054817086,-1.0733090048
H,0,1.0684030654,-1.7073707393,0.5241232659

Glycerol, conformation 9, Hadad conformation 45
H CBS-QB3 -344.27574 H_{rel} = +1.71 kcal/mol
H CBS-APNO -344.66710 H_{rel} = +1.78 kcal/mol
C,0,-1.4228985798,-0.2745868091,0.6103086602
C,0,0.0255990465,0.2039956697,0.5644905577
C,0,0.9784221217,-0.7922425485,-0.0825568856
O,0,-1.9365073092,-0.4695982736,-0.6931951958
O,0,2.2975253322,-0.2813069935,-0.1110214073
O,0,0.1024919012,1.3900805255,-0.2451689399
H,0,-1.6893281023,0.3231391513,-1.1769188262
H,0,-0.1533115568,2.1340004743,0.302286117
H,0,2.2148519164,0.5825630784,-0.5216965105
H,0,-2.0263083735,0.460019682,1.1694889738
H,0,-1.4899587728,-1.2343797606,1.134599687
H,0,0.3882993723,0.4178225315,1.5801798087
H,0,0.6123560954,-1.0338790074,-1.0895442245
H,0,1.0005259087,-1.7140607198,0.5079031854

Glycerol, conformation 10, Hadad conformation 120
H CBS-QB3 -344.27505 H_{rel} = +2.15 kcal/mol
H CBS-APNO -344.66654 H_{rel} = +2.13 kcal/mol
C,0,0.279878447,-0.0246226759,-0.0000347712
C,0,-0.1688758956,-0.7318237742,-1.2725443756
O,0,0.2046702343,0.004327447,2.422870203
O,0,0.2042754568,0.0018995413,-2.4229701216
O,0,-0.3042711495,1.2918878545,-0.0007059468
H,0,-0.1922993363,0.8691398677,2.2963124944
H,0,0.4157989476,1.9214989757,-0.0013642115
H,0,-0.192776878,0.86679927,-2.2972935146
H,0,0.3116058094,-1.7117738901,1.3485462921
H,0,-1.2593111487,-0.8793344802,1.2335434851
H,0,1.3738277608,0.0621781492,-0.0001188825
H,0,-1.259454318,-0.8806459367,-1.2325247365
H,0,0.3114840239,-1.7131056805,-1.3469677723

Glycerol, conformation 11, Hadad conformation 64
H CBS-QB3 -344.27473 H_{rel} = +2.35 kcal/mol
H CBS-APNO -344.66596 H_{rel} = +2.49 kcal/mol

C,0,1.1299500286,0.4452570693,-0.3921385636
C,0,-0.1240966972,0.6494243067,0.4612284628
C,0,-0.8741444741,-0.661890125,0.6781266353
O,0,1.813327382,-0.7099724367,0.0858668097
O,0,-1.2000794628,-1.2590229647,-0.5754007718
O,0,-0.959989298,1.6068657614,-0.1699938771
H,0,2.6553714432,-0.7517704659,-0.3678325413
H,0,-1.3799753669,1.1320973494,-0.893392233
H,0,-0.4062657529,-1.7157899877,-0.861192116
H,0,1.7561214925,1.345096089,-0.3345784355
H,0,0.815985539,0.3121242712,-1.4379045468
H,0,0.1535296911,1.0665986483,1.4359777953
H,0,-0.2883874313,-1.3430973112,1.3044881247
H,0,-1.823347093,-0.4409202041,1.1757452574

Glycerol, conformation 12, Hadad conformation 34
H CBS-QB3 -344.27473 H_{rel} = +2.35 kcal/mol
H CBS-APNO -344.66591 H_{rel} = +2.52 kcal/mol
C,0,-0.8432378637,-0.7137100141,0.6543527244
C,0,-0.018947716,0.5531071094,0.4682564978
C,0,1.1614492579,0.3312762118,-0.4748484433
O,0,-1.4310104378,-0.9939929541,-0.6183601973
O,0,2.0053271667,-0.6476323914,0.1201170427
O,0,-0.8393165399,1.603458934,-0.0145233513
H,0,-2.0870614237,-1.6780197226,-0.4887090696
H,0,-1.3679131726,1.2010068016,-0.7094038884
H,0,2.733073745,-0.797922257,-0.4829299264
H,0,-0.1988510331,-1.5356436912,0.9870958082
H,0,-1.6219025253,-0.5187035159,1.4043982369
H,0,0.3713021143,0.8691095178,1.4422839648
H,0,0.7817552979,-0.0042499757,-1.4501196823
H,0,1.6794031302,1.2913929473,-0.6045967161

Glycerol, conformation 13, Hadad conformation 9
H CBS-QB3 -344.27431 H_{rel} = +2.61 kcal/mol
H CBS-APNO -344.66590 H_{rel} = +2.53 kcal/mol
C,0,0.2239472142,0.699562896,1.2801594362
C,0,0.2209527948,-0.1247236791,0.
C,0,0.2239472142,0.699562896,-1.2801594362
O,0,0.2443690762,-0.1350361067,2.4203368664
O,0,0.2443690762,-0.1350361067,-2.4203368664
O,0,-0.9133727229,-1.0065276877,0.
H,0,-0.3862070967,-0.8348906225,2.232557975

H,0,-1.6950163278,-0.4452322396,0.
H,0,-0.3862070967,-0.8348906225,-2.232557975
H,0,-0.6613870049,1.360789276,1.2845839715
H,0,1.1171086741,1.332065827,1.3225727671
H,0,1.0878565262,-0.7931639346,0.
H,0,1.1171086741,1.332065827,-1.3225727671
H,0,-0.6613870049,1.360789276,-1.2845839715

Glycerol, conformation 14, Hadad conformation 83
H CBS-QB3 -344.27448 H_{rel} = +2.50 kcal/mol
H CBS-APNO -344.66574 H_{rel} = +2.63 kcal/mol
C,0,-1.113005307,0.5257071067,-0.3702219849
C,0,0.1539265879,0.6220037585,0.4921937999
C,0,0.800813182,-0.7412605907,0.6843608269
O,0,-1.9844742789,-0.504355808,0.0560434601
O,0,0.9450778403,-1.4234579971,-0.554141504
O,0,1.1254329636,1.432567197,-0.1699193208
H,0,-1.6035809901,-1.319911765,-0.27735416
H,0,0.7137215236,2.2747143635,-0.3713680368
H,0,1.4780918456,-0.8455811963,-1.1057488526
H,0,-0.7996129366,0.3869372114,-1.4127471714
H,0,-1.6734502786,1.4667805378,-0.3078019588
H,0,-0.0918313621,1.0356486245,1.4822209876
H,0,1.7691897829,-0.6085084991,1.1859386901
H,0,0.1635024275,-1.3657349433,1.3149892248

Glycerol, conformation 15, Hadad conformation 54
H CBS-QB3 -344.27448 H_{rel} = +2.50 kcal/mol
H CBS-APNO -344.66574 H_{rel} = +2.63 kcal/mol
C,0,-1.1084019796,0.5377947235,-0.3654717761
C,0,0.1634090385,0.6223077888,0.4909772978
C,0,0.7975578769,-0.7471486395,0.6815671113
O,0,-1.9881098743,-0.4831041122,0.0658858318
O,0,0.9292737488,-1.4319801678,-0.5568796783
O,0,1.1398686688,1.4224851932,-0.1764612238
H,0,-1.6169108927,-1.3027478504,-0.2684204466
H,0,0.7356431552,2.2684959377,-0.376878995
H,0,1.4654601933,-0.8599962842,-1.1115459722
H,0,-0.8012419267,0.3948737679,-1.4092902264
H,0,-1.659144362,1.4844712124,-0.3014374382
H,0,-0.0736238932,1.0393650661,1.481699989
H,0,1.7695238017,-0.6235627928,1.1785232657
H,0,0.1569834452,-1.3646098428,1.3157842611

Glycerol, conformation 16, Hadad conformation 115
H CBS-QB3 -344.27451 H_{rel} = +2.48 kcal/mol
H CBS-APNO -344.66569 H_{rel} = +2.66 kcal/mol
C,0,0.7864078548,-0.7887682529,0.2885766837
C,0,-0.0083491562,0.3650698672,-0.3038811
C,0,-1.4396497785,0.3922928318,0.205419398
O,0,2.123145424,-0.6273620928,-0.1868714046
O,0,-2.0514525595,-0.8356819774,-0.1672255572
O,0,0.5776293292,1.6007507861,0.0666927719
H,0,2.6768741827,-1.2398315009,0.296776286
H,0,1.5205316305,1.4757077881,-0.0673222842
H,0,-2.9718478851,-0.7820964816,0.0892337008
H,0,0.7507079751,-0.7109084278,1.385776778
H,0,0.3556849056,-1.7464848787,-0.025448454
H,0,-0.0216722221,0.2508125864,-1.3989260243
H,0,-1.4232799459,0.5244561385,1.2979339052
H,0,-1.9498977543,1.257001614,-0.240800699

Glycerol, conformation 17, Hadad conformation 2
H CBS-QB3 -344.2744 H_{rel} = +2.55 kcal/mol
H CBS-APNO -344.66569 H_{rel} = +2.66 kcal/mol
C,0,-0.8005403888,-0.7752549927,0.3137860493
C,0,0.016742869,0.3570588731,-0.2871090539
C,0,1.4456045863,0.3789807065,0.2435922203
O,0,-2.1172680874,-0.6374380664,-0.2196971606
O,0,2.1489310904,-0.8124738263,-0.0794363204
O,0,-0.5662154008,1.6056497122,0.0365798983
H,0,-2.6940264643,-1.2116107151,0.2838863798
H,0,-1.5076636663,1.4863173587,-0.1131604531
H,0,2.2556241116,-0.8203093816,-1.0321445715
H,0,-0.3553306468,-1.7434352037,0.0548557819
H,0,-0.8065355848,-0.6565683085,1.4074407371
H,0,0.0419209607,0.2150129124,-1.3824626935
H,0,1.9518079637,1.2749682286,-0.1410829573
H,0,1.4269046573,0.4447517029,1.3360791435

Glycerol, conformation 18, Hadad conformation 116
H CBS-QB3 -344.27436 H_{rel} = +2.60 kcal/mol
H CBS-APNO -344.66561 H_{rel} = +2.71 kcal/mol
C,0,0.7940004467,-0.7818619018,0.3401939077
C,0,-0.00433801,0.352853386,-0.292287214
C,0,-1.4407014543,0.3955870158,0.2008911905

O,0,2.1653066517,-0.6864867465,-0.048129078
O,0,-2.0579879052,-0.8242121601,-0.1895878769
O,0,0.5854417297,1.5971141476,0.0367232509
H,0,2.2084600441,-0.9458389121,-0.9707156257
H,0,1.5329367594,1.4409082307,-0.0034572363
H,0,-2.9740778625,-0.7786245823,0.0835338888
H,0,0.3606562797,-1.7545510568,0.081516365
H,0,0.7836283331,-0.6624045532,1.4287612127
H,0,-0.0180146969,0.2063676721,-1.3866576396
H,0,-1.4344925453,0.519024023,1.2944245236
H,0,-1.9375957704,1.2684904378,-0.2444436684

Glycerol, conformation 19, Hadad conformation 66
H CBS-QB3 -344.27420 H_{rel} = +2.68 kcal/mol
H CBS-APNO -344.66561 H_{rel} = +2.71 kcal/mol
C,0,1.127107371,0.3947034161,-0.4840765286
C,0,-0.0538477878,0.6010914343,0.4709386605
C,0,-0.8426066433,-0.68672336,0.6940571845
O,0,1.9450243728,-0.708049761,-0.1121794783
O,0,-1.3743276061,-1.158615293,-0.5422444727
O,0,-0.9001826081,1.6165775924,-0.0426697124
H,0,2.3943411871,-0.4645529013,0.6988763084
H,0,-1.4032305208,1.1884614629,-0.741978984
H,0,-0.6779959916,-1.662633033,-0.9671474426
H,0,1.7001525519,1.3282371742,-0.5599815275
H,0,0.7390698506,0.1645838009,-1.481736962
H,0,0.3158358618,0.9610067161,1.4411473725
H,0,-0.2179503701,-1.446919568,1.1767641906
H,0,-1.6999856673,-0.4654366806,1.3368373913

Glycerol, conformation 20, Hadad conformation 80
H CBS-QB3 -344.27413 H_{rel} = +2.72 kcal/mol
H CBS-APNO -344.66542 H_{rel} = +2.83 kcal/mol
C,0,1.1576705693,0.2988939083,-0.5152746036
C,0,-0.0014814791,0.5437895474,0.4569250665
C,0,-0.8423096772,-0.710238447,0.657701142
O,0,2.0613773074,-0.6835162338,-0.0251159777
O,0,-1.4807326943,-0.9576088182,-0.5954059924
O,0,-0.8134442463,1.613250865,0.0041535525
H,0,2.5070563414,-0.2960541364,0.7300834085
H,0,-1.3648268228,1.2277923783,-0.6827053225
H,0,-2.1200053409,-1.656896228,-0.462940636

H,0,1.6578006681,1.2570085764,-0.7117456747
H,0,0.7637481975,-0.0877622068,-1.4591685192
H,0,0.4103220986,0.8497013248,1.4284820149
H,0,-0.1966180925,-1.5472739684,0.9502983274
H,0,-1.587817829,-0.5186235615,1.4416902143

Glycerol, conformation 21, Hadad conformation 53
H CBS-QB3 -344.27417 H_{rel} = +2.70 kcal/mol
H CBS-APNO -344.66540 H_{rel} = +2.84 kcal/mol
C,0,-1.1403206395,0.3142154427,-0.480681385
C,0,0.0359891718,0.5238534536,0.4669604614
C,0,0.8638823864,-0.7438929489,0.6386485578
O,0,-2.068082434,-0.5386725764,0.173906574
O,0,1.4430906493,-1.1454110728,-0.5913565928
O,0,0.9321106446,1.4988780371,-0.063362998
H,0,-2.6011625896,-0.9536696016,-0.5034055831
H,0,0.4335338822,2.302930581,-0.2149071709
H,0,1.9143164567,-0.371024197,-0.9086266974
H,0,-1.5995530838,1.2895553187,-0.7131764626
H,0,-0.7590868492,-0.1263445104,-1.4097290513
H,0,-0.3548319205,0.8338386025,1.4479627448
H,0,1.6357185119,-0.5625559158,1.4009704388
H,0,0.2143538136,-1.5560986126,0.9731701642

Glycerol, conformation 22, Hadad conformation 78
H CBS-QB3 -344.27409 H_{rel} = +2.75 kcal/mol
H CBS-APNO -344.66536 H_{rel} = +2.87 kcal/mol
C,0,1.1537998404,0.4455497635,-0.3995433057
C,0,-0.0888264122,0.6468884965,0.4840017306
C,0,-0.8227440993,-0.6655707112,0.7151034176
O,0,1.9263039248,-0.6796122543,-0.0109073934
O,0,-1.0474738119,-1.2386369763,-0.5767341351
O,0,-0.9453406383,1.6121457551,-0.108087868
H,0,1.4677686371,-1.442766335,-0.3676796673
H,0,-1.2946271549,1.1891594173,-0.8970526215
H,0,-1.6741861519,-1.9543605873,-0.471041525
H,0,1.7968528481,1.327050666,-0.3230809773
H,0,0.8233517063,0.356456935,-1.4431829104
H,0,0.2059416146,1.0528793257,1.4579536458
H,0,-0.217989769,-1.3330869592,1.3419077684
H,0,-1.7724755337,-0.4502115358,1.2211908412

Glycerol, conformation 23, Hadad conformation 1

H CBS-QB3 -344.27376 H_{rel} = +2.96 kcal/mol
H CBS-APNO -344.66515 H_{rel} = +3.00 kcal/mol
C,0,-0.8076299271,-0.7726054255,0.3526605268
C,0,0.0121765227,0.3458291547,-0.2802608024
C,0,1.4447066274,0.3823453482,0.240600607
O,0,-2.1685762816,-0.6833410883,-0.0694626084
O,0,2.1560976433,-0.8016889743,-0.0928968838
O,0,-0.5756088618,1.598976369,0.010382576
H,0,-2.1930378877,-0.9590982374,-0.9878852954
H,0,-1.5238427916,1.449485786,-0.0401768782
H,0,2.2976591525,-0.7820189138,-1.0406956546
H,0,-0.8224455714,-0.6316489866,1.4383304382
H,0,-0.3691986826,-1.7516279384,0.1261197473
H,0,0.039763213,0.1795861724,-1.3754946748
H,0,1.9404314011,1.2848779447,-0.1420240953
H,0,1.429746444,0.4429797892,1.3334329977

Glycerol, conformation 24, Hadad conformation 86
H CBS-QB3 -344.27369 H_{rel} = +3.00 kcal/mol
H CBS-APNO -344.66490 H_{rel} = +3.16 kcal/mol
C,0,1.4306144539,0.3870851358,0.2216814638
C,0,-0.0075881336,0.3434761409,-0.2787153523
C,0,-0.803276416,-0.809949996,0.3146249227
O,0,2.0707603579,-0.8028525249,-0.2136670934
O,0,-2.1304153878,-0.8097649999,-0.1737101548
O,0,-0.6940906257,1.534654676,0.1154013795
H,0,2.9611046992,-0.7935108159,0.1376901064
H,0,-0.4795385721,2.2187731289,-0.5194047318
H,0,-2.4405676963,0.0876941032,-0.0308435661
H,0,1.9241667746,1.2838039294,-0.1852468784
H,0,1.4181465159,0.4711377896,1.3189121691
H,0,-0.0126883288,0.2412045895,-1.3729330578
H,0,-0.77962971,-0.7173739181,1.4132343774
H,0,-0.3439369313,-1.7599792385,0.0339644157

Glycerol, conformation 25, Hadad conformation 85
H CBS-QB3 -344.27324 H_{rel} = +3.28 kcal/mol
H CBS-APNO -344.66460 H_{rel} = +3.34 kcal/mol
C,0,1.4382809652,0.3827651553,0.2364468966
C,0,-0.0028457965,0.3418461084,-0.2697363602
C,0,-0.8133471278,-0.7913964789,0.3399213294
O,0,2.154549374,-0.7970453208,-0.0919636272
O,0,-2.1216817021,-0.8219123172,-0.1962158412

O,0,-0.6902066753,1.5424504472,0.0851154795
H,0,2.1989924146,-0.8400903124,-1.0489044798
H,0,-0.3788722489,2.2416925854,-0.4910178871
H,0,-2.4525539979,0.07268047,-0.0859135055
H,0,1.4346063089,0.4546371071,1.3285451214
H,0,1.9376954084,1.282886986,-0.1569001213
H,0,-0.0093957362,0.2110526585,-1.3647669155
H,0,-0.8279361689,-0.6561885782,1.433559373
H,0,-0.3395880173,-1.7494675103,0.1147895381

Glycerol, conformation 26, Hadad conformation 77
H CBS-QB3 -344.27323 H_{rel} = +3.29 kcal/mol
H CBS-APNO -344.66456 H_{rel} = +3.37 kcal/mol
C,0,-0.0503750701,1.2284753872,0.339834633
C,0,-0.648300106,-0.0190779031,-0.3217691057
C,0,0.0112358779,-1.3067351786,0.1559562575
O,0,1.2064789957,1.5832039644,-0.1985072004
O,0,1.4173589932,-1.1938899457,-0.0575992879
O,0,-2.0198030769,-0.173903541,0.0208187061
H,0,1.7381650748,0.7849581007,-0.1440508999
H,0,-2.4960304796,0.5530642868,-0.3825901597
H,0,1.8352753098,-1.9530736468,0.3486430963
H,0,-0.007694943,1.0663687283,1.4295739199
H,0,-0.7217940234,2.0766192727,0.1575492402
H,0,-0.5098611698,0.0737567875,-1.4094860448
H,0,-0.2219495996,-1.4282889285,1.2236859486
H,0,-0.4091907831,-2.156505384,-0.3973881032

Erythritol, global minimum
H CBS-QB3 -458.65062
H CBS-APNO -459.17224
C,0,0.0225368925,0.0203234899,-1.5782346891
H,0,-0.9240911588,-0.5362601826,-1.5286334584
H,0,0.5432882769,-0.2923746079,-2.493759265
C,0,0.9026607279,-0.376902366,-0.3870357791
H,0,1.7866458747,0.2785832891,-0.3839957137
C,0,0.2452410178,-0.310293027,0.9966987447
H,0,1.0326192703,-0.5566998714,1.7262454991
C,0,-0.3608723009,1.0349896028,1.3891097223
H,0,0.330185382,1.8507678247,1.1580531033
H,0,-0.553974619,1.0295513854,2.4699750246
O,0,-1.5701130352,1.2910542753,0.6821113549
H,0,-2.0734111372,0.4737306119,0.7543772464

O,0,-0.8172527337,-1.2519826005,1.0854928205
H,0,-0.4785402584,-2.0580143428,0.6858698671
O,0,1.2846505448,-1.7515164589,-0.5013803251
H,0,1.6402451947,-1.8824278693,-1.3819339603
O,0,-0.1709626158,1.4129101967,-1.6776995495
H,0,-0.8388563226,1.6245606505,-1.0152606424

Erythritol, conformation 2
H CBS-QB3 -458.64989 H_{rel} = +0.46 kcal/mol
C,0,-0.2447663243,0.1488550602,-1.5289505907
H,0,-0.2699340182,1.2373712887,-1.3947301742
H,0,-0.8189229018,-0.1033394581,-2.4241730703
C,0,-0.9365224574,-0.5325126576,-0.3454116764
H,0,-0.8581461426,-1.6161891617,-0.479826318
C,0,-0.428710558,-0.1596463849,1.0628621987
H,0,-1.0403686887,-0.7428179169,1.7596369711
C,0,1.0511391019,-0.4163709409,1.3672514464
H,0,1.3491256028,-1.416827583,1.0324828463
H,0,1.198888644,-0.3564317031,2.447570177
O,0,1.8720331319,0.5981732271,0.7955979058
H,0,1.9219700987,0.3908660405,-0.1527571844
O,0,-0.7289233529,1.2169752442,1.3074409313
H,0,0.0961785975,1.6903720634,1.1212494834
O,0,-2.3195963658,-0.2009527974,-0.4159158796
H,0,-2.3972914512,0.6584176959,0.0217882575
O,0,1.1111412926,-0.3204515711,-1.6527370875
H,0,1.4427057915,-0.0754904451,-2.5213792364

Erythritol, conformation 3
H CBS-QB3 -458.64985 H_{rel} = +0.48 kcal/mol
C,0,-0.0820013785,0.1590833887,-1.401735779
H,0,-0.3360972084,1.2207101223,-1.3567363785
H,0,-1.0039693712,-0.4288340122,-1.3442776679
C,0,0.8420470587,-0.2303058262,-0.2521945499
H,0,1.7151469047,0.4380141554,-0.2653651197
C,0,0.2171724964,-0.1979946659,1.147977044
H,0,1.0279149452,-0.4301678773,1.8538431414
C,0,-0.4149761668,1.1271308903,1.5627697227
H,0,0.2726425401,1.9548950258,1.3712102862
H,0,-0.6095073589,1.0931384626,2.6428423033
O,0,-1.6115239964,1.396412141,0.8463191578
H,0,-2.1281052769,0.5823324162,0.8995804912
O,0,-0.8110600913,-1.1828971596,1.2497798671

H,0,-0.4606121589,-1.9673979596,0.8067885471
O,0,1.2666840884,-1.5850152167,-0.4341098008
H,0,1.4121034945,-1.6850563409,-1.3845445696
O,0,0.6510353477,-0.1603924104,-2.5961473602
H,0,0.0531061314,-0.1036551336,-3.3459983352

Erythritol, conformation 4
H CBS-QB3 -458.64983 H_{rel} = +0.50 kcal/mol
C,0,0.5607211531,0.2678051097,-1.4553984819
H,0,1.2571919368,0.9981964773,-1.0402337232
H,0,0.3811324622,0.5333964034,-2.5009422054
C,0,-0.7790447969,0.3445016417,-0.7088461204
H,0,-1.4388774124,-0.4518802877,-1.0768754986
C,0,-0.7078065597,0.2613778018,0.8301821765
H,0,-1.7432937213,0.3352286141,1.1798679057
C,0,-0.0693580982,-0.9932409806,1.4378811844
H,0,-0.4642268048,-1.9000073555,0.9621185241
H,0,-0.3240455693,-1.0310978954,2.4991366342
O,0,1.3505284791,-0.9325078132,1.3727373637
H,0,1.5903100039,-1.1343622576,0.4525702126
O,0,-0.0274716512,1.4199697562,1.3171065535
H,0,0.8831301161,1.1298973555,1.4790093993
O,0,-1.3991545312,1.5770163561,-1.0592033163
H,0,-1.0036243536,2.2362223691,-0.4717171443
O,0,1.2208274344,-1.0053614516,-1.3605452353
H,0,0.7130619128,-1.6551528435,-1.8568472285

Erythritol, conformation 5
H CBS-QB3 -458.64910 H_{rel} = +0.95 kcal/mol
C,0,-0.1159608469,0.0918336771,-1.437671631
H,0,-0.3840869035,1.1498415446,-1.4371798661
H,0,-1.0325842479,-0.4954875908,-1.3900867816
C,0,0.7876950519,-0.2647973963,-0.2535500122
H,0,1.6587901874,0.4111904328,-0.2509559129
C,0,0.1338651189,-0.2141947181,1.133867046
H,0,0.9236223229,-0.45987235,1.8592642275
C,0,-0.4764185414,1.1260031843,1.5294065834
H,0,0.2446052341,1.9331158574,1.3755076558
H,0,-0.7188895531,1.0909013289,2.5996627097
O,0,-1.6297498956,1.4403672012,0.7626492923
H,0,-2.1851925042,0.6514720533,0.7969161547
O,0,-0.9167256352,-1.1756974311,1.2134473886
H,0,-0.5678030249,-1.9760825193,0.7988685393

O,0,1.2270546403,-1.6168872242,-0.4089345649
H,0,1.3177178081,-1.7501684071,-1.3633136881
O,0,0.5314105712,-0.2998997371,-2.6601871747
H,0,1.2026522179,0.3583630945,-2.8677099658

Erythritol, conformation 6
H CBS-QB3 -458.64904 H_{rel} = +0.99 kcal/mol
C,0,-0.2140773132,-0.1101282255,-1.643546675
H,0,-1.161803057,-0.6305598915,-1.4531823515
H,0,0.1904635443,-0.4968706051,-2.5807846101
C,0,0.791812175,-0.4646719466,-0.5416197278
H,0,1.7071440956,0.1102013087,-0.7293339406
C,0,0.3434466122,-0.2323337706,0.9162070382
H,0,1.216496786,-0.4550863096,1.5529968377
C,0,-0.1415498239,1.168394298,1.2904364807
H,0,0.5410500205,1.9317593081,0.9139273422
H,0,-0.1801814012,1.2444910069,2.3832334834
O,0,-1.4306801077,1.446338406,0.7436394593
H,0,-1.9615663916,0.6586001211,0.9260282724
O,0,-0.7290550986,-1.114943995,1.2421191538
H,0,-0.5392208389,-1.9423449033,0.7784893119
O,0,1.0369761902,-1.8788983419,-0.6849248014
H,0,1.8715743005,-2.0909217338,-0.2549262114
O,0,-0.3877023308,1.2833373751,-1.7963107093
H,0,-0.9531293613,1.573635899,-1.0624493524

Erythritol, conformation 7
H CBS-QB3 -458.64870 H_{rel} = +1.20 kcal/mol
C,0,-0.8010393678,-0.1430253385,-1.7407443
H,0,-1.4764619353,-0.9960959122,-1.5977110155
H,0,-0.3321644271,-0.2295991039,-2.7269907593
C,0,0.2769588524,-0.1823758264,-0.6665169996
H,0,0.9731469893,0.6463291774,-0.8455830631
C,0,-0.3040430999,0.0286483008,0.7531777979
H,0,-1.2506690582,-0.5258056316,0.8523876993
C,0,0.6497995029,-0.4624536471,1.8319762152
H,0,0.7087211623,-1.5509304098,1.8147721679
H,0,0.2660794784,-0.1490183769,2.8100793323
O,0,1.979362108,0.0165565559,1.6199620149
H,0,1.9164995193,0.9784637153,1.5864921104
O,0,-0.5079746987,1.4217113922,0.9884358762
H,0,-1.0201118324,1.7504242803,0.2364143652
O,0,0.9348631933,-1.434113275,-0.8112005189

H,0,1.7606047027,-1.3605832098,-0.3178072246
O,0,-1.5014765094,1.1013230692,-1.6068235874
H,0,-2.2720925798,1.09054524,-2.180319111

Erythritol, conformation 8
H CBS-QB3 -458.64863 H_{rel} = +1.25 kcal/mol
C,0,0.7523789157,0.2865070474,-1.5806897758
H,0,1.6240214829,0.9417762376,-1.4942741756
H,0,-0.1390043244,0.9202650839,-1.7013683046
C,0,0.6093062956,-0.5839433413,-0.3352418966
H,0,1.4875519722,-1.2338704497,-0.2798897841
C,0,0.4786259909,0.1603842417,1.003452191
H,0,1.4227588457,0.6780447678,1.2115674982
C,0,-0.6569215183,1.1855175271,1.0509782905
H,0,-0.554419422,1.9391033119,0.2681411261
H,0,-0.6350935264,1.6958259198,2.0201673965
O,0,-1.9311625844,0.5578151451,0.8468109366
H,0,-2.08119787,0.0073867686,1.6247018495
O,0,0.265025501,-0.7940511852,2.0474094238
H,0,-0.0806714845,-1.5861547119,1.614131377
O,0,-0.5013905231,-1.4858876223,-0.5038470726
H,0,-1.3013981919,-0.9469390992,-0.3888164001
O,0,0.953274848,-0.5218812391,-2.7267511164
H,0,0.2883155931,-1.2198964022,-2.6764815634

Erythritol, conformation 9
H CBS-QB3 -458.64851 H_{rel} = +1.32 kcal/mol
C,0,-0.1553971395,-0.1020829678,-1.7386526296
H,0,0.2845801867,-0.3937145135,-2.6946058069
H,0,-0.2600378293,0.9894033934,-1.7345401165
C,0,0.7526164287,-0.5760782971,-0.5970954062
H,0,1.7821329189,-0.259271116,-0.8030420956
C,0,0.3591045534,-0.052029904,0.7927400879
H,0,1.0673829343,-0.4682504036,1.5187192056
C,0,0.3428441564,1.4592903704,0.9686598984
H,0,-0.4012099336,1.9075081515,0.2947768321
H,0,1.3214369273,1.8759420339,0.717719817
O,0,0.0768445511,1.8109581748,2.3157710614
H,0,-0.7185204707,1.3248922623,2.563459258
O,0,-0.9730211935,-0.498803492,1.1395750326
H,0,-0.9138870778,-1.4505341807,1.2819252267
O,0,0.7352791886,-2.0047688916,-0.5515049881
H,0,-0.0844580425,-2.2647175231,-0.9954825553

O,0,-1.4237178397,-0.753916803,-1.6743648631
H,0,-1.7919723187,-0.5438242938,-0.8040559585

Erythritol, conformation 10
H CBS-QB3 -458.64850 H_{rel} = +1.33 kcal/mol
C,0,0.125254285,0.1643931305,-1.7942992846
H,0,-0.1171037922,0.7838343939,-2.6597946498
H,0,-0.6036934539,-0.6554816725,-1.7452527225
C,0,0.0718641936,1.0312673077,-0.5257572537
H,0,-0.8660660017,1.5993507299,-0.5143822321
C,0,0.12955091,0.2129622025,0.7748602253
H,0,0.4277158065,0.9017504722,1.5750065291
C,0,-1.2029174011,-0.4220947978,1.1555538495
H,0,-1.57372021,-1.0479862827,0.3339300399
H,0,-1.9428670319,0.3589824795,1.3713929125
O,0,-0.9403704941,-1.2187916676,2.3155305891
H,0,-1.6874297548,-1.8032690452,2.4688743893
O,0,1.1180874883,-0.8183568901,0.6459003731
H,0,0.9816858798,-1.4112448227,1.3963384335
O,0,1.1214718871,1.9855021005,-0.5707520116
H,0,1.8639926086,1.5261825111,-0.9882876995
O,0,1.4462189746,-0.3213149159,-2.0072497537
H,0,1.6483281062,-0.8656842334,-1.2316127336

Erythritol, conformation 11
H CBS-QB3 -458.64815 H_{rel} = +1.55 kcal/mol
C,0,0.0921508244,-0.0903338595,-1.6145054056
H,0,0.6540405071,-0.3546205351,-2.5131960434
H,0,-0.0613527006,0.9904033105,-1.5905527605
C,0,0.8670650159,-0.5648275444,-0.3793950606
H,0,1.9088674162,-0.2316083661,-0.4651610775
C,0,0.325608814,-0.075431184,0.9769117416
H,0,1.0132778806,-0.4572411085,1.7440523533
C,0,0.2105035519,1.4368089592,1.1617831263
H,0,1.1199914509,1.9289915416,0.8082218537
H,0,0.1087635369,1.650536677,2.2333057431
O,0,-0.8733429435,2.0029873508,0.4369111576
H,0,-1.66779208,1.5506798381,0.7425791481
O,0,-0.9896737614,-0.6083921721,1.2112396743
H,0,-0.8940509349,-1.567820865,1.1734472385
O,0,0.8616612139,-1.9968298268,-0.3471251377
H,0,0.134709792,-2.260393124,-0.9299310389
O,0,-1.1517404223,-0.7919679874,-1.7188206363

H,0,-1.6586891613,-0.5609411042,-0.929763876

Erythritol, conformation 12
H CBS-QB3 -458.64805 H_{rel} = +1.61 kcal/mol
C,0,0.2255964462,-0.258014348,-1.6471885027
H,0,0.7494759158,0.0022253857,-2.5726110223
H,0,-0.845947963,-0.3666400773,-1.8622166135
C,0,0.450298312,0.8777236229,-0.6478086593
H,0,-0.1171354119,1.7408685748,-1.0137514165
C,0,-0.0111972206,0.556807297,0.7862706136
H,0,0.0852612847,1.4691171027,1.3804913307
C,0,-1.4402875649,0.0399887677,0.9310780778
H,0,-1.5543449888,-0.897852758,0.3674325994
H,0,-2.1558906435,0.7663667684,0.5344596222
O,0,-1.7601291857,-0.1529074025,2.2989502409
H,0,-0.9942350139,-0.5967846842,2.6846150724
O,0,0.8998017288,-0.3740327668,1.3982100218
H,0,0.928899863,-1.1449163521,0.8098087264
O,0,1.8138901273,1.272671655,-0.6213571107
H,0,2.2392377493,0.7049922868,0.0351039478
O,0,0.7507437477,-1.4761034806,-1.0952142377
H,0,0.7359628174,-2.1635085915,-1.7662726905

Erythritol, conformation 13
H CBS-QB3 -458.64802 H_{rel} = +1.63 kcal/mol
C,0,0.5093938473,-0.8811181902,-1.4177917582
H,0,0.5553537901,-1.9698886543,-1.2978597004
H,0,1.498901791,-0.4677917445,-1.2115307334
C,0,-0.5340426511,-0.2768638084,-0.4781055912
H,0,-1.4988465843,-0.7775110569,-0.6472988222
C,0,-0.2042956044,-0.4562237888,1.0139579169
H,0,0.1858533071,-1.4729608898,1.1446331971
C,0,0.8307523061,0.5559029099,1.5310281069
H,0,1.7081474459,0.599085019,0.8725112509
H,0,1.1648209834,0.24866178,2.5237092125
O,0,0.2305136081,1.8368776789,1.6893868015
H,0,-0.0532550054,2.0988857336,0.8008211177
O,0,-1.3779890621,-0.3686238097,1.8076602936
H,0,-1.462094065,0.5694697422,2.0313999693
O,0,-0.6698925196,1.1181404502,-0.7808500402
H,0,-0.5720437796,1.1913225343,-1.7403440886
O,0,0.222171794,-0.5143274482,-2.7723959617

H,0,-0.5334496015,-1.0330374572,-3.0689341703

Erythritol, conformation 14
H CBS-QB3 -458.64764 H_{rel} = +1.87 kcal/mol
C,0,-0.8623640024,-0.114572707,-1.7442154409
H,0,-1.6375425611,-0.8668121415,-1.5836429623
H,0,-0.4153314761,-0.2946792281,-2.7267027502
C,0,0.2122920487,-0.2447592978,-0.6650185019
H,0,0.9606999978,0.5451750444,-0.8148712204
C,0,-0.3629767732,-0.0538613813,0.7562784268
H,0,-1.2805040395,-0.6544851928,0.858585077
C,0,0.6141063478,-0.4946360616,1.8364114293
H,0,0.7444306575,-1.5770098495,1.806361236
H,0,0.1983133839,-0.2239701712,2.8144823053
O,0,1.9079549736,0.0768045997,1.6470867866
H,0,1.7799704442,1.0326014939,1.6391685291
O,0,-0.6331736494,1.3292563003,0.9888751194
H,0,-1.1834913075,1.6243409001,0.24908823
O,0,0.7976306724,-1.5298762574,-0.8413179331
H,0,1.642635839,-1.5169526726,-0.3786232246
O,0,-1.5207329604,1.161911698,-1.6946245986
H,0,-0.9619185954,1.8015249243,-2.1473195074

Erythritol, conformation 15
H CBS-QB3 -458.64750 H_{rel} = +1.96 kcal/mol
C,0,-0.0630150342,0.0348577199,-1.5885893154
H,0,0.4531269045,0.0390468043,-2.5548409
H,0,-0.626566636,0.95840331,-1.4716219411
C,0,0.969409555,-0.1336612617,-0.4688720385
H,0,1.7503321931,0.6250985633,-0.5954506916
C,0,0.399760045,-0.0187421267,0.9547199505
H,0,1.2359608989,-0.1779246005,1.6456833427
C,0,-0.2405299025,1.3259190394,1.3187872493
H,0,0.4121579398,2.1512023216,1.0205903474
H,0,-0.3523479184,1.3643643255,2.4097249388
O,0,-1.4915943197,1.5470105333,0.6812232785
H,0,-2.0818665782,0.843982217,0.9748798787
O,0,-0.5194437548,-1.094848758,1.198092807
H,0,-1.0495290262,-1.1864808879,0.3876971024
O,0,1.5921900543,-1.4173964883,-0.6196805908
H,0,1.1800132531,-1.994249705,0.035998965
O,0,-1.0276855188,-1.0323778956,-1.5517356915
H,0,-0.5403711551,-1.8342031107,-1.7766076914

Erythritol, conformation 16
H CBS-QB3 -458.64716 H_{rel} = +2.17 kcal/mol
C,0,0.1378626982,-0.0387937521,-1.6442046025
H,0,0.5432314334,-0.5143063177,-2.544442747
H,0,0.2277040219,1.042000464,-1.7658369991
C,0,0.9229210959,-0.558882329,-0.4392457997
H,0,1.985802623,-0.3692943644,-0.6428824513
C,0,0.6000823763,0.0727579964,0.9730121599
H,0,1.5565537361,0.2263025819,1.4798945202
C,0,-0.177693391,1.3979334501,0.9679893388
H,0,0.2234171785,2.093220874,0.220612648
H,0,-0.0747494322,1.8674349366,1.9480918608
O,0,-1.5699629634,1.1621009176,0.7931790063
H,0,-1.6997624712,0.7603797053,-0.0862491999
O,0,-0.1290911068,-0.8623983569,1.7710324895
H,0,-1.0541095909,-0.5693822857,1.7174112022
O,0,0.700852471,-1.9646406735,-0.3931930024
H,0,0.3646609457,-2.1374300314,0.5021495545
O,0,-1.2584421743,-0.3301627131,-1.5264054686
H,0,-1.2992784502,-1.2768401021,-1.3309115098

Erythritol, conformation 17
H CBS-QB3 -458.64686 H_{rel} = +2.36 kcal/mol
C,0,0.4597813214,0.268834973,-1.6188934733
H,0,1.248096599,0.9376964447,-1.2422367491
H,0,0.2237783616,0.5889288144,-2.6349919658
C,0,-0.8188306541,0.4571356759,-0.7875501335
H,0,-1.5607090921,-0.2599181834,-1.154163997
C,0,-0.687534647,0.2870140716,0.7417075166
H,0,-1.6975348554,0.4227560096,1.1402775551
C,0,-0.165496418,-1.0493563514,1.2616186729
H,0,-0.653355315,-1.8841295469,0.7478211633
H,0,-0.3711999686,-1.1120939438,2.3343976636
O,0,1.2613842262,-1.0838883396,1.0492278177
H,0,1.6503651294,-1.7429390538,1.6310360008
O,0,0.0902848233,1.3579893221,1.2842255184
H,0,1.0096768439,1.0633775254,1.2608787577
O,0,-1.3513856005,1.7571226075,-1.02473699
H,0,-0.8468166029,2.3523831968,-0.4545074068
O,0,0.8983111951,-1.0785780017,-1.6920292121
H,0,1.3111836536,-1.2823342205,-0.8420797384

Erythritol, conformation 18
H CBS-QB3 -458.64684 H_{rel} = +2.37 kcal/mol
C,0,0.5020270707,0.2051633765,-1.6745288627
H,0,-0.4647913002,0.7052707947,-1.7699238335
H,0,0.6372031727,-0.4147410279,-2.5686762729
C,0,0.4999299133,-0.7045494264,-0.4428205906
H,0,1.42983204,-1.2852870752,-0.4256641386
C,0,0.3992885654,0.0364223499,0.9046452971
H,0,1.3155901728,0.6127717342,1.0609044354
C,0,-0.7880897286,0.9941417722,1.0016781817
H,0,-0.7453366834,1.7584966272,0.2248883676
H,0,-0.7609617035,1.4962623835,1.974887505
O,0,-2.0368953282,0.3024521448,0.8333660869
H,0,-2.1401263286,-0.2522788329,1.6153351523
O,0,0.3024109035,-0.9340197905,1.9514545627
H,0,-0.0589965776,-1.7290763715,1.5357601689
O,0,-0.5431711062,-1.6800097877,-0.5438767745
H,0,-1.3735047578,-1.1930056481,-0.4206775076
O,0,1.477000747,1.2412778188,-1.5895708337
H,0,2.3485899286,0.8407099583,-1.6671819435

Erythritol, conformation 19
H CBS-QB3 -458.64641 H_{rel} = +2.64 kcal/mol
C,0,0.4135970532,0.5880054677,-1.543099374
H,0,1.0969108436,0.273730409,-2.338771708
H,0,0.3496442968,1.6848932268,-1.5438486465
C,0,1.0151611044,0.1222184555,-0.2193816225
H,0,1.9967215414,0.6037190235,-0.1569889025
C,0,0.2589094429,0.5102572626,1.0727251809
H,0,0.2371441023,1.6025013886,1.1621711124
C,0,-1.1949924537,0.0071749967,1.1387150281
H,0,-1.8594772129,0.7242986398,0.6449380826
H,0,-1.4701860379,-0.0587741647,2.1925244225
O,0,-1.3847309359,-1.2956788306,0.5654885702
H,0,-1.5401467005,-1.1309123227,-0.3761730272
O,0,1.0242510467,0.044172395,2.1794976781
H,0,1.3579754325,-0.8257377899,1.9222431598
O,0,1.280976114,-1.2803140518,-0.2064797471
H,0,0.4248171888,-1.709733015,-0.04397933
O,0,-0.8859931909,0.0164427411,-1.7620985635
H,0,-1.1205816347,0.1237361682,-2.6874823132

Erythritol, conformation 20

H CBS-QB3 -458.64630 H_{rel} = +2.71 kcal/mol
C,0,0.3071373051,0.1309145065,-1.6777117823
H,0,0.7142747858,-0.4091463324,-2.5386557566
H,0,0.671087061,1.1600709049,-1.7243123693
C,0,0.7761283605,-0.5609292103,-0.3851019403
H,0,1.8030995556,-0.9031815607,-0.5439509919
C,0,0.7903687297,0.3150377254,0.8849442988
H,0,1.5439065772,1.1016351087,0.7599606703
C,0,-0.549625444,1.0047276693,1.1912795872
H,0,-0.6367693331,1.9330441238,0.6139917389
H,0,-0.5561073251,1.2585490082,2.2522406193
O,0,-1.6937789711,0.1723974378,0.9417980174
H,0,-1.9073282828,0.3181605255,0.0072353144
O,0,1.2347252487,-0.4797585093,1.9793347913
H,0,0.8482857545,-1.3561519572,1.8561528168
O,0,0.0049372084,-1.7451432861,-0.1425584827
H,0,-0.8106096354,-1.4361072404,0.2887722143
O,0,-1.1217708507,0.1983303493,-1.794245495
H,0,-1.4179597444,-0.702448263,-1.9691722506

Erythritol, conformation 21
H CBS-QB3 -458.64544 H_{rel} = +3.25
C,0,-0.1595327226,0.6321755074,-1.627621456
H,0,0.3224911288,0.959153297,-2.5504952145
H,0,-0.9279931406,1.3744848981,-1.3692531779
C,0,0.8963218998,0.5448956186,-0.5136862943
H,0,1.5862974189,1.3882667823,-0.6125413412
C,0,0.3584956425,0.6035771283,0.9491028911
H,0,0.1889437768,1.6599224534,1.1942752218
C,0,-0.9690075852,-0.099323605,1.2341313908
H,0,-1.8054506782,0.4661641865,0.8001202495
H,0,-1.0904583757,-0.1243022306,2.3204970424
O,0,-0.9528280529,-1.4376085828,0.7036663259
H,0,-1.6150192756,-1.962317554,1.1623514586
O,0,1.3726916945,0.1340787788,1.8173963155
H,0,1.8156997102,-0.5708256626,1.3213520581
O,0,1.6832165909,-0.6311446712,-0.6901967799
H,0,1.0999157608,-1.276650117,-1.1157651985
O,0,-0.7256460795,-0.6399313079,-1.9226606502
H,0,-1.0781357127,-1.0206139193,-1.1006728412

Threitol, global minimum
H CBS-QB3 -458.65433

H CBS-APNO -459.17582
C,0,-0.5678011279,0.4712800157,0.5131198758
H,0,-1.0054520368,0.327719464,1.5106344106
C,0,0.6777286882,-0.421368217,0.4134341049
H,0,1.3342015188,-0.1784532076,1.2603218855
O,0,-1.5578884649,0.0867270669,-0.4426246798
H,0,-1.4148196932,0.6814445985,-1.1884863618
O,0,1.4125804445,-0.1527030843,-0.7821467089
H,0,1.1069208945,-0.8212428716,-1.4067218139
C,0,0.3291154257,-1.9138932425,0.4523553018
H,0,1.241567151,-2.499513578,0.6009232957
H,0,-0.3605545788,-2.1184175921,1.2839752522
O,0,-0.2113032344,-2.3239441135,-0.7958057966
H,0,-1.0066204023,-1.7899622962,-0.9047840586
C,0,-0.2385157095,1.955029351,0.3112881782
H,0,0.6235270005,2.2378182447,0.9325606394
H,0,-1.0984058157,2.5645149292,0.6057505979
O,0,-0.009432465,2.2237206423,-1.0647300002
H,0,0.7451534055,1.6712448908,-1.2990651223

Threitol, conformation 2
H CBS-QB3 -458.65426 H_{rel} = +0.04 kcal/mol
C,0,-0.087908876,0.0741280133,0.8840047772
H,0,0.6204888324,-0.0503349523,1.7189571001
C,0,0.754639891,0.2645946858,-0.3939545966
H,0,1.4462718445,1.1003227517,-0.1998736492
O,0,-0.9773418893,-1.038356458,0.8105565356
H,0,-0.5535425015,-1.7163290984,0.2590954891
O,0,-0.0336952761,0.5247065258,-1.5536864658
H,0,-0.8200169845,1.0185012125,-1.268684115
C,0,1.5826054671,-0.9722390422,-0.7468301779
H,0,2.275352332,-0.7249447314,-1.5593141956
H,0,2.162229496,-1.3207424365,0.1102238608
O,0,0.7241777496,-2.0493914963,-1.1383914415
H,0,0.1741259219,-1.6780918767,-1.8429001313
C,0,-0.9823311655,1.2771979606,1.1880387901
H,0,-0.4136541405,2.2090760691,1.1749972384
H,0,-1.4290727156,1.1548789536,2.1813379194
O,0,-2.0072671288,1.3975462596,0.1953160895
H,0,-2.4350608568,0.5294776598,0.1811079728

Threitol, conformation 3
H CBS-QB3 -458.65099 H_{rel} = +2.10 kcal/mol

C,0,-0.4668724221,-0.4748902499,0.9349001524
H,0,-0.3333959654,-1.3373776513,1.6039506836
C,0,-0.1552747943,-0.9807990189,-0.495082481
H,0,-0.8705717937,-1.7890615561,-0.6996037284
O,0,-1.830068334,-0.0871309256,1.0095384372
H,0,-1.8173267633,0.8794035065,0.9374033579
O,0,1.1938436335,-1.4561959818,-0.6010032865
H,0,1.3297564529,-2.163258594,0.0369601121
C,0,-0.3060702356,0.0378832901,-1.6196827688
H,0,-0.2747292074,-0.4885746445,-2.5797824736
H,0,-1.2651588947,0.5480270422,-1.5356217979
O,0,0.7137984241,1.0429856946,-1.5901163314
H,0,1.5551341934,0.570045076,-1.556369841
C,0,0.4224771261,0.6637805306,1.454653254
H,0,1.4764007513,0.449424477,1.2451709944
H,0,0.2939732588,0.7372218928,2.536878536
O,0,0.011810103,1.9164787724,0.9202748165
H,0,0.3222734674,1.9320373399,-0.0024686356

Threitol, conformation 4
H CBS-QB3 -458.65050 H_{rel} = +2.40 kcal/mol
C,0,-0.2391836998,0.5966842463,0.9981278809
H,0,-1.0369384532,0.7113297351,1.7400021747
C,0,-0.9491858789,0.3762759842,-0.3558563287
H,0,-1.6005585448,1.2460626087,-0.5022768783
O,0,0.4939107196,1.8113466569,0.9450730023
H,0,1.4138485769,1.5417981872,0.8013092756
O,0,-1.7276587351,-0.8308638679,-0.2412883081
H,0,-2.5280691993,-0.7340315783,-0.7652913947
C,0,-0.0404670088,0.2568673947,-1.5807370888
H,0,-0.6440494739,0.3509245683,-2.4932190227
H,0,0.7040042647,1.0532168852,-1.5839460083
O,0,0.6602874571,-0.9902337735,-1.5960148766
H,0,-0.0055870096,-1.6608759465,-1.389058566
C,0,0.6834319453,-0.539057227,1.4640705182
H,0,0.1905339154,-1.5091443974,1.3415207134
H,0,0.8960106983,-0.394029743,2.5254253984
O,0,1.9418488771,-0.4822088961,0.7987545224
H,0,1.7878195491,-0.8040588369,-0.1065950139

Other Compounds, for reductions and isodesmic equations
H_2
H CBS-QB3

H CBS-APNO -1.16198
H,0,-2.2168688021,1.99152539,0.
H,0,-2.9599672779,1.99152539,0.

Two carbons
ethane
H CBS-APNO -79.74355
C,0,0.,-0.0000003823,0.7661987807
H,0,0.0000000002,1.0218907408,1.1610326419
H,0,0.8849836396,-0.5109461005,1.1610318206
H,0,-0.8849836398,-0.5109461002,1.1610318206
C,0,0.,0.0000003823,-0.7661987807
H,0,0.8849836398,0.5109461002,-1.1610318206
H,0,-0.8849836396,0.5109461005,-1.1610318206
H,0,-0.0000000002,-1.0218907408,-1.1610326419

ethanol, global minimum, *anti*
H CBS-APNO -154.95424
C,0,0.2183866907,-0.4481776432,-0.0042047854
C,0,1.738570034,-0.4549752253,0.0081820295
H,0,-0.152416816,0.5820434074,-0.0113944965
H,0,-0.1520521445,-0.9568987299,-0.9002410412
H,0,-0.1766816119,-0.9595743688,0.8810609912
H,0,2.1065490283,-1.4926096059,0.0256938211
H,0,2.1061833238,0.0485418626,0.9157777358
O,0,2.1812727117,0.2209195358,-1.1619208592
H,0,3.1385182939,0.2121807872,-1.1464325052

ethanol, *gauche* conformation
H CBS-APNO -154.95405 H_{rel} = +0.12 kcal/mol
H,0,0.0057163292,-0.0248242315,-0.0137281394
C,0,0.0022926822,-0.0160774846,1.0840920393
H,0,1.0413366271,-0.0033111859,1.4293135942
H,0,-0.4934849928,0.9049938135,1.413114581
C,0,-0.713760963,-1.2485830451,1.6289635655
H,0,-1.7588268649,-1.2628066079,1.2826334234
H,0,-0.7292227377,-1.2250243338,2.7229625154
O,0,-0.0466867903,-2.4538277011,1.281077825
H,0,-0.0117537764,-2.4787250151,0.32361396560

acetaldehyde
H CBS-QB3 -153.57761
H CBS-APNO -153.76592

C,0,0.2386355687,-0.4044053749,0.
H,0,0.2986376898,-1.5150016926,0.
O,0,1.2436045381,0.265790972,0.
C,0,-1.1657141155,0.1590359209,0.
H,0,-1.7049057935,-0.2022340984,0.884489444
H,0,-1.7049057935,-0.2022340984,-0.884489444
H,0,-1.1313520945,1.2510483716,0.

Three carbons
propane
H CBS-APNO -119.02627
C,0,0.003284881,0.,0.0014182617
C,0,-0.0044461562,0.,1.5340504039
C,0,1.4094118342,0.,2.1256810052
H,0,1.3857458919,0.,3.2216330921
H,0,1.9675022317,0.8863586524,1.7986140509
H,0,1.9675022317,-0.8863586524,1.7986140509
H,0,-1.0148200942,0.,-0.4049477222
H,0,0.5223876123,-0.8863586433,-0.3845481403
H,0,0.5223876123,0.8863586433,-0.3845481403
H,0,-0.552016906,-0.8801972549,1.8965075857
H,0,-0.552016906,0.8801972549,1.8965075857

1-propanol, global minimum
H CBS-QB3 -193.98920
H CBS-APNO -194.23686
C,0 ,-0.1090494324,-0.1172098468,1.4405534731
H,0,0.5262354848,0.7655125541,1.5570834102
H,0,0.5194981721,-1.0080877686,1.5682979081
H,0,-0.8600742577,-0.1156686281,2.2387147262
C,0,-0.7721219137,-0.1200459818,0.0595957336
H,0,-1.4159611903,0.7619215861,-0.0513659939
H,0,-1.4113172818,-1.0064035227,-0.052002861
C,0,0.2424272307,-0.1168692172,-1.0764484066
H,0,0.8899289101,-1.0051187404,-0.9913935285
H,0,-0.2822092398,-0.1681417117,-2.0430855116
O,0,1.0071144863,1.0789459341,-0.9777892292
H,0,1.6655290318,1.051165343,-1.6721597203

1-propanol, conformer 2
H CBS-QB3 -193.98880 H_{rel} = +0.25 kcal/mol
H CBS-APNO -194.23666 H_{rel} = +0.13 kcal/mol
C,0,-0.1256445436,-0.1300754728,1.3381291665

H,0,0.4406388175,0.7926322353,1.5146201607
H,0,0.5580364301,-0.9814020086,1.4541305439
H,0,-0.8864792482,-0.1988586915,2.122973263
C,0,-0.7644083422,-0.1278214067,-0.0555137268
H,0,-1.4209662486,0.7449941081,-0.1609122309
H,0,-1.3862673536,-1.0252957174,-0.1784284171
C,0,0.2661569715,-0.0977631232,-1.1850884762
H,0,0.9013601806,-0.9973651871,-1.1315136103
H,0,-0.2412799082,-0.1074025197,-2.1549458976
O,0,1.059145144,1.0819328134,-1.1766197645
H,0,1.5997081006,1.0464249701,-0.3868310109

1-propanol, conformer 3
H CBS-QB3 -193.98883 H_{rel} = +0.23 kcal/mol
H CBS-APNO -194.23664 H_{rel} = +0.14 kcal/mol
C,0,-0.018387794,-0.1875722877,1.4005758742
H,0,0.6246536387,0.691116847,1.5095059218
H,0,0.6068664945,-1.0795001255,1.5323541413
H,0,-0.7665060639,-0.1747369102,2.2012031162
C,0,-0.6819428656,-0.1966096776,0.0201078035
H,0,-1.3575228257,0.6670477904,-0.075383614
H,0,-1.303728923,-1.0949475061,-0.0975425674
C,0,0.3335180683,-0.1539181873,-1.1221257656
H,0,1.0179413551,-1.006277883,-1.0456771121
H,0,-0.1849403605,-0.2264122908,-2.0906905772
O,0,1.1581601841,1.0038313918,-1.0744747817
H,0,0.5718880919,1.7579778391,-1.157852439

1-propanol, conformer 4
H CBS-QB3 -193.98886 H_{rel} = +0.21 kcal/mol
H CBS-APNO -194.23663 H_{rel} = +0.14 kcal/mol
C,0,0.0255945853,0.,1.5704749382
H,0,0.6566734858,0.8869544696,1.7080817474
H,0,0.6566734858,-0.8869544696,1.7080817474
H,0,-0.7310000894,0.,2.3625800468
C,0,-0.6279535357,0.,0.1849740489
H,0,-1.2679514428,0.8828914903,0.0653391359
H,0,-1.2679514428,-0.8828914903,0.0653391359
C,0,0.40133085,0.,-0.936012781
H,0,1.0440679107,0.8906582941,-0.8432880046
H,0,1.0440679107,-0.8906582941,-0.8432880046
O,0,-0.2964723988,0.,-2.1740453073
H,0,0.3629216817,0.,-2.8682377048

1-propanol, conformer 5
H CBS-QB3 -193.98872 H_{rel} = +0.30 kcal/mol
H CBS-APNO -194.23659 H_{rel} = +0.17 kcal/mol
C,0,0.1418203937,0.0759886277,1.5282307603
H,0,0.7645607735,0.9723168225,1.638006543
H,0,0.7873753796,-0.8008166383,1.6629829995
H,0,-0.5974520972,0.0781652088,2.3364328074
C,0,-0.5374764721,0.0521127158,0.1550627137
H,0,-1.1887932456,0.9262660059,0.0322199822
H,0,-1.1785615078,-0.838393907,0.0735364899
C,0,0.4684653614,0.0402070271,-0.994368038
H,0,1.1050728701,0.9303804175,-0.9422863196
H,0,1.1237913226,-0.8417608861,-0.9055384132
O,0,-0.1576330841,0.0859887417,-2.268140687
H,0,-0.731169694,-0.6804551358,-2.3161398382

2-propanol, global minimum
H CBS-QB3 -193.99603
H CBS-APNO -194.24385
H,0,0.3318440111,1.2464035918,-1.7225588587
C,0,0.3464488748,1.2327014166,-0.6262010264
H,0,1.3874609224,1.2425426561,-0.2831814271
H,0,-0.1424562306,2.1488667735,-0.2696242436
C,0,-0.363055355,-0.0067262452,-0.0821710949
H,0,-1.4109609071,0.0026238728,-0.4247532771
C,0,0.2933652411,-1.2998606391,-0.5466379033
O,0,-0.3240435464,-0.0268970202,1.3431340231
H,0,1.3387884039,-1.3232807898,-0.2176410345
H,0,0.263274134,-1.3777685775,-1.6394202334
H,0,-0.2262039047,-2.1614403218,-0.1151022662
H,0,-0.7004035372,0.8017566127,1.643823307

2-propanol, conformer 2, Cs
H CBS-QB3 -193.99549 H_{rel} = +0.33 kcal/mol
H CBS-APNO -194.24346 H_{rel} = +0.24 kcal/mol
H,0,0.3750950258,1.2270474511,-1.7385111062
C,0,0.3617359952,1.2316230489,-0.6419934718
H,0,1.4025769774,1.2426679895,-0.2894079632
H,0,-0.1292271153,2.1467861301,-0.2957058325
C,0,-0.3662146577,0.0048600052,-0.0926452255
H,0,-1.4027207494,0.0139508079,-0.4493703351
C,0,0.2981124463,-1.3032686323,-0.5217285715

O,0,-0.4780562187,0.0749908363,1.3263367499
H,0,1.3379308406,-1.3329883084,-0.1672103044
H,0,0.3090793247,-1.403134346,-1.6137251969
H,0,-0.2372188521,-2.1558359084,-0.0915738891
H,0,0.4187739993,0.0686531587,1.6679718161

propanal, global minimum, Cs symmetry
H CBS-QB3 -192.80249
H CBS-APNO -193.04914
C,0,0.3537436665,0.,1.3647781786
H,0,1.2822744486,0.,1.977497584
O,0,-0.7328486223,0.,1.8940623362
C,0,0.5768759314,0.,-0.1353433799
H,0,1.1984501128,0.8766873367,-0.3703438121
H,0,1.1984501128,-0.8766873367,-0.3703438121
C,0,-0.7215045442,0.,-0.9407913147
H,0,-1.3230720346,0.8826309632,-0.7021807753
H,0,-1.3230720346,-0.8826309632,-0.7021807753
H,0,-0.5092960375,0.,-2.0151552258

propanal, conformer 2
H CBS-QB3 -192.80078 H_{rel} = +1.07 kcal/mol
H CBS-APNO -193.04758 H_{rel} = +0.98 kcal/mol
C,0,-0.3755360457,-0.0037674616,1.3504749266
H,0,0.2288849453,0.8581602675,1.7151736882
O,0,-0.7870734675,-0.8378533486,2.1219310097
C,0,-0.6032150316,-0.0246329802,-0.147866441
H,0,-1.1877919343,-0.917082036,-0.3919796467
H,0,-1.1993160555,0.8625788152,-0.4051084395
C,0,0.7280504338,0.0091365338,-0.9145487249
H,0,1.3283894708,-0.8797567642,-0.6901340646
H,0,1.3154175268,0.8947634671,-0.6434372868
H,0,0.5521901579,0.038453507,-1.994507021

acetone
H CBS-QB3 -192.81316
H CBS-APNO -193.06028
O,0,1.4133443322,0.0050976581,-0.0000037351
C,0,0.2012801851,0.000341091,-0.000001202
C,0,-0.6098880536,1.2882373792,0.021553459
H,0,0.0557857708,2.1448118056,-0.1034593155
H,0,-1.3669202624,1.2786353715,-0.7714024746
H,0,-1.1383674828,1.371047154,0.9795736469

C,0,-0.5997551941,-1.2938818421,-0.0215525897
H,0,-1.356838152,-1.2902208958,0.7714045194
H,0,-1.1275701814,-1.3808373307,-0.9795718221
H,0,0.0726211003,-2.1452053193,0.1034595503

Four carbons
butane, *anti*
H CBS-APNO -158.30914
C,0,-1.9583234134,0.121402043,0.
H,0,-2.7474933358,-0.6395049254,0.
C,0,-0.5653507352,-0.5175088696,0.
H,0,-2.0964720753,0.7528148316,-0.8866688863
H,0,-2.0964720753,0.7528148316,0.8866688863
C,0,0.5653507352,0.5175088696,0.
H,0,-0.4572335895,-1.166491667,-0.8808258555
H,0,-0.4572335895,-1.166491667,0.8808258555
C,0,1.9583234134,-0.121402043,0.
H,0,0.4572335895,1.166491667,-0.8808258555
H,0,0.4572335895,1.166491667,0.8808258555
H,0,2.7474933358,0.6395049254,0.
H,0,2.0964720753,-0.7528148316,-0.8866688863
H,0,2.0964720753,-0.7528148316,0.8866688863

butane, *gauche*
H CBS-APNO -158.30809 H_{rel} = +0.66 kcal/mol
C,0,0.1034144093,-1.5644296597,0.5655374745
H,0,-0.2598579775,-1.1463453543,1.5110234606
H,0,1.1969037025,-1.6385005878,0.6274006954
H,0,-0.2993228142,-2.5800141011,0.4750268906
C,0,-0.3124749032,-0.7015919345,-0.6326539059
H,0,-1.4078931133,-0.6065182751,-0.6526309552
H,0,-0.0267015375,-1.2155641528,-1.56046837
C,0,0.3124749032,0.7015919362,-0.632653904
H,0,1.4078931133,0.6065182769,-0.6526309535
H,0,0.0267015375,1.215564157,-1.5604683667
C,0,-0.1034144093,1.5644296581,0.5655374787
H,0,0.2598579775,1.1463453503,1.5110234636
H,0,-1.1969037025,1.6385005861,0.6274006997
H,0,0.2993228142,2.5800140998,0.4750268975

1-butanol, global minimum
H CBS-APNO -233.51963
H,0,-1.1774071931,-0.9668710074,2.1449199331

C,0,-0.5186824017,-0.090274264,2.096351487
H,0,-1.1492542806,0.8066621993,2.1387378102
H,0,0.1193106944,-0.0971800182,2.9878096106
C,0,0.3181085375,-0.1044199346,0.8128666249
H,0,0.9615794352,-0.9964717128,0.8025073834
H,0,0.9832989351,0.7657697057,0.7873962559
C,0,-0.5466875436,-0.1026315301,-0.4519912408
H,0,-1.2029797683,0.7786120844,-0.4514763334
H,0,-1.1948311914,-0.9910234844,-0.4601635116
C,0,0.2734238148,-0.0906788473,-1.7349612566
H,0,-0.3982374902,-0.1508823304,-2.6053448962
H,0,0.9371507808,-0.9706643171,-1.7537012433
O,0,1.0286003244,1.1152719777,-1.7606227016
H,0,1.5666063466,1.0947824791,-2.5523269217

1-butanol, conformer 2
H CBS-APNO -233.51939 H_{rel} = +0.16 kcal/mol
H,0,-1.1453339968,-1.0546020088,2.0368083462
C,0,-0.5460482715,-0.1360507283,2.0145515173
H,0,-1.2342279564,0.7165540862,2.066084822
H,0,0.0835814674,-0.1192939992,2.9115960321
C,0,0.2990408247,-0.0703898081,0.737859302
H,0,0.9932426143,-0.9236456787,0.710732688
H,0,0.9124141481,0.8410927092,0.7631278026
C,0,-0.5530713345,-0.0746727887,-0.5366467772
H,0,-1.2061200724,0.8085892393,-0.5422709994
H,0,-1.2017662644,-0.9631496453,-0.5358291087
C,0,0.274119129,-0.0790275,-1.8226515927
H,0,-0.3889021105,-0.0939713975,-2.6936333977
H,0,0.8956216135,-0.9887923301,-1.8610355527
O,0,1.0746547258,1.0870518459,-1.9669329429
H,0,1.7427944839,1.0503050041,-1.281760139

1-butanol, conformer 3
H CBS-APNO -233.51938 H_{rel} = +0.16 kcal/mol
H,0,-1.1065292814,-1.0211388235,2.1080011226
C,0,-0.4478992209,-0.1449107458,2.055715175
H,0,-1.0789479881,0.7518869157,2.0996567458
H,0,0.1934559326,-0.1501219603,2.9446369211
C,0,0.3842861989,-0.1616730566,0.7692834403
H,0,1.0293403909,-1.0518508501,0.7575105009
H,0,1.0512490559,0.7077570982,0.7405363671
C,0,-0.4807567381,-0.162441667,-0.4951739769

H,0,-1.163380277,0.702315028,-0.472939444
H,0,-1.1154951887,-1.0607994981,-0.5128818102
C,0,0.3380570639,-0.1129388169,-1.7850227927
H,0,-0.328229699,-0.1953525555,-2.6576968126
H,0,1.0357942896,-0.9572323502,-1.8176433446
O,0,1.1453015338,1.0549312933,-1.8730904377
H,0,0.5437519277,1.8015699886,-1.8608936541

1-butanol, conformer 4
H CBS-APNO -233.51926 H_{rel} = +0.23 kcal/mol
H,0,-1.0341671911,-0.8866495632,2.2235638219
C,0,-0.3895862997,0.,2.1812363746
H,0,-1.0341671911,0.8866495632,2.2235638219
H,0,0.2471542893,0.,3.0735532261
C,0,0.4483784282,0.,0.8979496417
H,0,1.1055573108,-0.8812773264,0.8898067608
H,0,1.1055573108,0.8812773264,0.8898067608
C,0,-0.4148696397,0.,-0.3676888568
H,0,-1.0672070917,0.8834475693,-0.3760231276
H,0,-1.0672070917,-0.8834475693,-0.3760231276
C,0,0.4126036256,0.,-1.6446794193
H,0,1.0612090961,-0.8908945133,-1.6618019434
H,0,1.0612090961,0.8908945133,-1.6618019434
O,0,-0.4838670577,0.,-2.7480779553
H,0,0.0494014153,0.,-3.5433820355

1-butanol, conformer 5
H CBS-APNO -233.51922 H_{rel} = +0.26 kcal/mol
H,0,-0.9357204437,-0.8364808824,2.2025360225
C,0,-0.3048636155,0.0591031765,2.1420523501
H,0,-0.9617267359,0.9367582396,2.1814667307
H,0,0.3417428502,0.0788989132,3.0268771448
C,0,0.5190369224,0.056903794,0.8497212066
H,0,1.1880012665,-0.8153374709,0.8412822074
H,0,1.1640597948,0.946443435,0.8239067337
C,0,-0.3550814568,0.0373123614,-0.4086243171
H,0,-1.0171894543,0.9134261012,-0.4228835144
H,0,-1.003979124,-0.8525876326,-0.3875790787
C,0,0.4575333268,0.0265012252,-1.7018317814
H,0,1.1159047768,-0.8572820609,-1.7204131888
H,0,1.0960654959,0.9154567976,-1.7494326011
O,0,-0.3625397481,0.076845543,-2.8607656755
H,0,-0.9412448553,-0.68596054,-2.8163102387

1-butanol, conformer 6
H CBS-APNO -233.51892 H_{rel} = +0.45 kcal/mol
H,0,0.1993554858,1.9250962271,1.1053758611
C,0,0.7704547892,0.9934523331,1.2142991742
H,0,1.535087547,0.9673414813,0.4297982837
H,0,1.2884545985,1.0269752128,2.1798570443
C,0,-0.1491613152,-0.2308590821,1.1329038169
H,0,-0.810528748,-0.2475616469,2.0096317227
H,0,0.4569194547,-1.1428174788,1.1699312285
C,0,-1.0206849153,-0.2651753499,-0.1311683498
H,0,-1.7073673968,0.5940495842,-0.1342685912
H,0,-1.6366598186,-1.1739734981,-0.1228205023
C,0,-0.2213013511,-0.2360758483,-1.4288610138
H,0,0.3017637827,0.7272366541,-1.5294110246
H,0,-0.9105464521,-0.3339768808,-2.2818328089
O,0,0.7059120836,-1.3150564606,-1.3963359198
H,0,1.1983022557,-1.2886552471,-2.2171009208

1-butanol, conformer 7
H CBS-APNO -233.51873 H_{rel} = +0.56 kcal/mol
H,0,0.266983284,1.9827065733,1.0577158134
C,0,0.838505902,1.0511145656,1.1630588418
H,0,1.5980635346,1.0243443678,0.3741020405
H,0,1.3611776461,1.0829615151,2.1259834703
C,0,-0.0851993753,-0.1702236622,1.0858155567
H,0,-0.7399099529,-0.1913238136,1.9672668124
H,0,0.521464931,-1.0830818333,1.1171716492
C,0,-0.9627384427,-0.1960388094,-0.1741068296
H,0,-1.6325867067,0.6767119517,-0.1809829861
H,0,-1.6092444203,-1.0867285314,-0.1453577994
C,0,-0.1710122476,-0.2064210913,-1.4832621907
H,0,0.404259397,0.7177408373,-1.5945713795
H,0,-0.8661369928,-0.2643331891,-2.3351498506
O,0,0.7837958321,-1.259288296,-1.5332145628
H,0,0.2925766116,-2.0781385845,-1.4444685856

1-butanol, conformer 8
H CBS-APNO -233.51852 H_{rel} = +0.70 kcal/mol
H,0,0.1760151374,1.9356157135,1.0433989134
C,0,0.7524880444,1.0075779217,1.1455671905
H,0,1.5195063323,1.0058858313,0.361271504
H,0,1.2698883085,1.0347038627,2.1113324599

C,0,-0.167431436,-0.2172704451,1.051962769
H,0,-0.8352550593,-0.2335550807,1.9226130098
H,0,0.4305102555,-1.1370332436,1.1151038114
C,0,-1.025135826,-0.256281333,-0.2222150683
H,0,-1.6908562517,0.6194960205,-0.2409007114
H,0,-1.6606177594,-1.1509816754,-0.1994940514
C,0,-0.216359911,-0.2734016647,-1.5218122002
H,0,0.3329442809,0.6720729249,-1.6462663653
H,0,-0.8961202017,-0.3685684154,-2.3746288391
O,0,0.6655269401,-1.3860147917,-1.6039296727
H,0,1.3448961459,-1.252244625,-0.9420027496

1-butanol, conformer 9
H CBS-APNO -233.51818 H_{rel} = +0.91 kcal/mol
H,0,1.888777141,-0.3477696977,1.2349460917
C,0,0.9821717353,-0.9641067899,1.2843238428
H,0,1.0400633283,-1.7123163307,0.4857429576
H,0,0.9881227182,-1.4993689773,2.240841166
C,0,-0.2724128638,-0.0894397525,1.1558508235
H,0,-0.3540913629,0.5529335259,2.0422415414
H,0,-1.1669867008,-0.7287499971,1.1513451815
C,0,-0.2780317574,0.7996105314,-0.0959248196
H,0,0.6002387704,1.4594335849,-0.0959469323
H,0,-1.1653459644,1.4508421018,-0.0708237276
C,0,-0.2964747547,0.0196750098,-1.4105008755
H,0,-1.1536129806,-0.674207691,-1.4155662391
H,0,0.6135981232,-0.5771706279,-1.5216928154
O,0,-0.3254899981,0.8777956478,-2.5426124741
H,0,-1.1005254335,1.4328394624,-2.4422247209

1-butanol, conformer 10
H CBS-APNO -233.51813 H_{rel} = +0.94 kcal/mol
H,0,0.2985192972,1.8310605424,1.2932567752
C,0,0.9002154714,0.9149961715,1.3510861703
H,0,1.6639572716,0.9645946393,0.5664364008
H,0,1.4182949131,0.9105439704,2.3171193453
C,0,0.0092091138,-0.3258852425,1.2016553923
H,0,-0.6473308356,-0.4026306422,2.0781062146
H,0,0.635489423,-1.2296201846,1.2035576766
C,0,-0.8643760447,-0.3168741784,-0.0602004907
H,0,-1.5227688612,0.5624581063,-0.0545960127
H,0,-1.5108127425,-1.2037259352,-0.0698290304
C,0,-0.0652301564,-0.3169373934,-1.3569446331

H,0,0.6494838554,-1.1566754583,-1.3440394247
H,0,0.5095832045,0.616250965,-1.4527244577
O,0,-0.9894591969,-0.4492032487,-2.4303319665
H,0,-0.4847727127,-0.3983511115,-3.2425529593

1-butanol, conformer 11
H CBS-APNO -233.51791 H_{rel} = +1.08 kcal/mol
H,0,1.7810072989,-0.3845109949,1.2789493011
C,0,0.8630874786,-0.9857978671,1.3070835384
H,0,0.9258288841,-1.7334323155,0.508401696
H,0,0.840418822,-1.522571233,2.2625041034
C,0,-0.3742828968,-0.0904272055,1.1539569134
H,0,-0.4639152184,0.5526161008,2.0391323736
H,0,-1.2790055544,-0.7145849182,1.1308205557
C,0,-0.3483072926,0.7996606255,-0.096896497
H,0,0.5428787221,1.4467994737,-0.0659608755
H,0,-1.2230831003,1.4625747829,-0.1018292343
C,0,-0.3519145671,0.0254550099,-1.415247384
H,0,-1.2126195257,-0.6524890489,-1.4444041114
H,0,0.5575876025,-0.5859892663,-1.5108807806
O,0,-0.4970996806,0.8849937225,-2.5386577976
H,0,0.2394170277,1.497701134,-2.5069718014

1-butanol, conformer 12
H CBS-APNO -233.51713 H_{rel} = +1.57 kcal/mol
H,0,0.1096724211,1.7350191752,1.4035852587
C,0,0.7479815148,0.843248969,1.3685847194
H,0,1.4819222307,0.9933050235,0.5738603371
H,0,1.2741839521,0.7643126297,2.3278937263
C,0,-0.0992872005,-0.4112671432,1.1175950091
H,0,-0.7664976853,-0.566730457,1.976706571
H,0,0.5540949328,-1.2951597155,1.0711029727
C,0,-0.9623519379,-0.3695967007,-0.1536660884
H,0,-1.5646059499,0.549317014,-0.1715691105
H,0,-1.6655384117,-1.2142828556,-0.1283250442
C,0,-0.1952617328,-0.4643206863,-1.4679879727
H,0,-0.9107307497,-0.6307413629,-2.2883911102
H,0,0.4859712245,-1.3304424716,-1.430712195
O,0,0.5286350921,0.7433469929,-1.6797921907
H,0,0.9818132997,0.6539925885,-2.5188848823

1-butanol, conformer 13
H CBS-APNO -233.51705 H_{rel} = +1.62 kcal/mol

H,0,1.5895738155,-0.8802169339,0.4868552897
C,0,0.8688910732,-0.7548267313,1.2980965624
H,0,0.2597734343,-1.6670483193,1.3553050227
H,0,1.4156192545,-0.6585353598,2.2437880093
C,0,-0.0253147365,0.4710172951,1.0665958081
H,0,0.5992209132,1.3731261833,0.9933081692
H,0,-0.6690275517,0.6112010082,1.945899849
C,0,-0.925381684,0.3938778107,-0.1767252586
H,0,-1.6515263964,1.2190905251,-0.1413158041
H,0,-1.51515654,-0.536755558,-0.1491600215
C,0,-0.2009254237,0.4845723856,-1.5215638145
H,0,0.4512917571,1.3652796228,-1.5325263773
H,0,-0.9425799684,0.6101380504,-2.3261472804
O,0,0.6477228897,-0.623554517,-1.7971188852
H,0,0.0978201632,-1.407366462,-1.745291269

1-butanol, conformer 14
H CBS-APNO -233.51669 H_{rel} = +1.85 kcal/mol
H,0,0.3263861684,1.6955653365,1.0710167154
C,0,0.8180429025,0.7283293397,1.2334401293
H,0,1.6611605727,0.6438821422,0.5349499271
H,0,1.246124836,0.7320436523,2.2423402129
C,0,-0.1746428619,-0.4318175188,1.0689363803
H,0,-0.8745823906,-0.418346037,1.9149002408
H,0,0.3725616498,-1.3831555974,1.1345701714
C,0,-0.9924909713,-0.4151643609,-0.2326178141
H,0,-1.6394205945,0.4722878565,-0.2607579268
H,0,-1.6519159051,-1.2946569455,-0.2333892407
C,0,-0.1702414678,-0.4276696316,-1.5267047307
H,0,-0.8016164781,-0.7601699805,-2.3566909891
H,0,0.6616569301,-1.1458082123,-1.4356767094
O,0,0.3026448364,0.8571385069,-1.9137835784
H,0,0.9163317731,1.1475404497,-1.240533788

2-butanol, global minimum
H CBS-APNO -233.52668
H,0,0.4093459987,1.8313909474,-1.5213771891
C,0,0.4063777784,1.8065240122,-0.4250452249
H,0,1.4409233249,1.8314532796,-0.0638817471
H,0,-0.1051936925,2.7096419862,-0.0668719051
C,0,-0.2903636824,0.5490536915,0.0926526059
H,0,-1.3315641357,0.5368180434,-0.2737039267
C,0,0.3988224297,-0.7302775482,-0.3736870717

H,0,1.4310161215,-0.7145920813,0.0023677292
H,0,0.4500857917,-0.7134578495,-1.4708063565
C,0,-0.3155716588,-1.9991071394,0.1017424651
H,0,0.2032708811,-2.8975885498,-0.2518668934
H,0,-1.3442734639,-2.0291284599,-0.2799767703
H,0,-0.3570454675,-2.0258143753,1.1943526585
O,0,-0.2840895577,0.520380592,1.5191907316
H,0,-0.7117396676,1.3247034512,1.8169108945

2-butanol, conformer 2
H CBS-APNO -233.52658 H_{rel} = +0.06 kcal/mol
H,0,0.4512741361,1.923526396,-1.4943322183
C,0,0.4185826312,1.8990084259,-0.3990540265
H,0,1.4445506548,1.915927129,-0.0132008692
H,0,-0.1022352743,2.7917071937,-0.0381589706
C,0,-0.2917796245,0.6437891536,0.089370146
H,0,-1.3291896779,0.6492807145,-0.2881074558
C,0,0.3936455494,-0.6365942753,-0.3994011406
H,0,1.4376306071,-0.616515316,-0.0591434458
H,0,0.4043296046,-0.625822152,-1.4980002091
C,0,-0.2892388583,-1.9173365502,0.0939367947
H,0,0.1902054339,-2.8076665271,-0.327179673
H,0,-1.3477919406,-1.9343548799,-0.1975262768
H,0,-0.2297529561,-2.0021143083,1.1859073542
O,0,-0.3009178506,0.7179605205,1.5140924159
H,0,-0.8493134348,-0.0007955245,1.830799575

2-butanol, conformer 3
H CBS-APNO -233.52625 H_{rel} = + 0.27 kcal/mol
H,0,0.2775182193,1.8861829064,-1.5209067233
C,0,0.3210099649,1.8538113701,-0.4257247885
H,0,1.3781855242,1.8740318658,-0.1260116794
H,0,-0.168720137,2.7465290514,-0.0233870907
C,0,-0.3558010859,0.5952962481,0.1160961272
H,0,-1.408032277,0.5882152,-0.1956827363
C,0,0.3146084822,-0.6876414326,-0.3855353141
H,0,1.3580108568,-0.6915187597,-0.0324169417
H,0,0.3550323818,-0.6593111809,-1.4832106539
C,0,-0.4015399707,-1.9574006323,0.0838010362
H,0,0.1257520858,-2.8570831809,-0.2534870434
H,0,-1.4232464148,-1.9923937539,-0.3146227297
H,0,-0.4655662378,-1.9772502004,1.1759608644
O,0,-0.4090625856,0.6242112903,1.5414575603

H,0,0.5018501939,0.6543212086,1.8436711129

2-butanol, conformer 4
H CBS-APNO -233.52595 H_{rel} = +0.46 kcal/mol
H,0,0.1253668828,1.7305065012,-0.9490055044
C,0,0.1760772789,1.6284227442,0.1400185766
H,0,1.2279204032,1.6315281415,0.4504067228
H,0,-0.322794109,2.4898006529,0.595721642
C,0,-0.4977733717,0.3451221515,0.6085330375
H,0,-1.532865213,0.3261586096,0.2253116315
C,0,0.2180349793,-0.9245866219,0.1367902386
H,0,-0.2972995854,-1.7899505754,0.5811503149
H,0,1.2345561177,-0.9143425191,0.552447587
C,0,0.2592306682,-1.091812049,-1.38630375
H,0,0.674659566,-2.0682196036,-1.6592568986
H,0,0.8802119669,-0.3218642344,-1.8569553801
H,0,-0.7490617739,-1.0226708366,-1.814292046
O,0,-0.5134774122,0.4055272521,2.0332823575
H,0,-0.8827863979,-0.4236216132,2.3421514706

2-butanol, conformer 5
H CBS-APNO -233.52589 H_{rel} = +0.50 kcal/mol
H,0,0.1956443404,1.6271061231,-0.9541936637
C,0,0.1951070508,1.5399804925,0.1378525114
H,0,1.2298607759,1.5601682613,0.4998608183
H,0,-0.3275776958,2.4173870898,0.5412858623
C,0,-0.488141717,0.2542424767,0.6030832672
H,0,-1.5193254579,0.234403074,0.2099287668
C,0,0.2271340799,-1.0134004921,0.1412582521
H,0,-0.2755532072,-1.8634439252,0.6188553979
H,0,1.2537007295,-0.9880547383,0.5309739225
C,0,0.2361851122,-1.1998731607,-1.3797810307
H,0,0.6711126829,-2.1695005421,-1.6465076728
H,0,0.8244716998,-0.4232585946,-1.8814632867
H,0,-0.7836116151,-1.1668023597,-1.7841848108
O,0,-0.5147968418,0.1904267117,2.0275590187
H,0,-0.9242099367,1.0006205838,2.3354746475

2-butanol, conformer 6
H CBS-APNO -233.52553 H_{rel} = +0.72 kcal/mol
H,0,0.0564537466,1.6803391456,-0.9584472659
C,0,0.1060451701,1.5850973386,0.1318619851
H,0,1.1641648658,1.6057235361,0.4293491733

H,0,-0.3961290386,2.4473127628,0.5823010896
C,0,-0.5528784927,0.2953714489,0.62224763
H,0,-1.5954399738,0.272514821,0.2801315922
C,0,0.1535272774,-0.9720887338,0.1304061417
H,0,-0.3437492866,-1.8271662831,0.6047544762
H,0,1.1896067886,-0.9588460523,0.5041188423
C,0,0.1532742756,-1.1431829916,-1.3931793703
H,0,0.5726707346,-2.1155262055,-1.6745604795
H,0,0.7475801709,-0.3675398247,-1.8887541313
H,0,-0.8693419524,-1.0909542591,-1.7880170162
O,0,-0.6440327644,0.290188753,2.0452243285
H,0,0.2582474789,0.2987555441,2.3725610043

2-butanol, conformer 7
H CBS-APNO -233.52538 H_{rel} = +0.82 kcal/mol
H,0,-0.102801491,1.6155797925,-1.4809648843
C,0,-0.1922462597,1.6139131547,-0.3882991785
H,0,0.8056406409,1.713596329,0.0507815963
H,0,-0.7880932189,2.4793375045,-0.0805600731
C,0,-0.8594527434,0.3307904088,0.0919846689
H,0,-1.8914312742,0.3089427945,-0.2957639931
C,0,-0.1528360225,-0.9476420291,-0.3814806713
H,0,-0.2208531606,-1.0016427729,-1.4770640228
H,0,-0.7184469152,-1.8088191579,0.0060971324
C,0,1.308934541,-1.0588098473,0.0665778107
H,0,1.7136512508,-2.047730882,-0.1769775442
H,0,1.3870929555,-0.9086284568,1.1481907475
H,0,1.9379828244,-0.3101273459,-0.427354254
O,0,-0.8877131869,0.4041257621,1.5173495917
H,0,-1.3394299403,-0.3828862543,1.8274830739

2-butanol, conformer 8
H CBS-APNO -233.52531 H_{rel} = +0.86 kcal/mol
H,0,-0.1207968317,1.5231678333,-1.5068601872
C,0,-0.1916324316,1.5212478937,-0.4123449207
H,0,0.8129937324,1.6230508408,0.0104202667
H,0,-0.7772764695,2.3997757135,-0.1106366931
C,0,-0.8591992818,0.2404258634,0.0905001727
H,0,-1.8930887161,0.2122075943,-0.2916819289
C,0,-0.1598353596,-1.0406870396,-0.3687666982|
H,0,-0.2206990065,-1.0931137696,-1.4644454667
H,0,-0.7354563967,-1.8868503561,0.0275056094
C,0,1.2992522959,-1.1573275989,0.0879570869

H,0,1.7055492202,-2.13965013,-0.1790020759
H,0,1.367456853,-1.0374669911,1.1734433281
H,0,1.9328424964,-0.3973420421,-0.3835786958
O,0,-0.8784877331,0.2069687454,1.5172002481
H,0,-1.2816213715,1.025593443,1.8102889548

2-butanol, conformer 9
H CBS-APNO -233.52490 H_{rel} = +1.12 kcal/mol
H,0,-0.2364750225,1.5674119584,-1.5088631012
C,0,-0.2839305293,1.5689366938,-0.4131586922
H,0,0.7376986968,1.6825110103,-0.0281817169
H,0,-0.8664679054,2.4357620731,-0.0844824844
C,0,-0.927640857,0.2846303755,0.1122930257
H,0,-1.9695483752,0.2519809777,-0.2278286616
C,0,-0.2344103516,-0.9958866369,-0.3780678305
H,0,-0.2931780205,-1.0271653453,-1.4750283126
H,0,-0.8109791396,-1.8481310252,0.002783277
C,0,1.227758229,-1.1386257605,0.0636352337
H,0,1.6359597521,-2.1003969644,-0.2657589425
H,0,1.3128512351,-1.1075973033,1.1569965398
H,0,1.8613197793,-0.3481599636,-0.3545191068
O,0,-1.0206777614,0.3136399428,1.5352411768
H,0,-0.1322807297,0.4610879675,1.8649405957

butanal, Cs, global minimum
H CBS-APNO -232.33163
C,0,0.3211737488,0.,2.0742125677
H,0,1.2150064612,0.,2.73678371
O,0,-0.7924809289,0.,2.5444366983
C,0,0.6251181396,0.,0.5886931743
H,0,1.2587517822,0.8769931178,0.3832192166
H,0,1.2587517822,-0.8769931178,0.3832192166
C,0,-0.6198147563,0.,-0.2983653922
H,0,-1.2312371213,0.8766711967,-0.0532074075
H,0,-1.2312371213,-0.8766711967,-0.0532074075
C,0,-0.2694407305,0.,-1.7898764583
H,0,0.3201724008,0.8868974141,-2.0543802494
H,0,-1.1749370527,0.,-2.407146418
H,0,0.3201724008,-0.8868974141,-2.0543802494

butanal, conformer 2
H CBS-APNO -232.33152 H_{rel} = +0.07 kcal/mol
C,0,1.2268232869,0.9953914845,0.8450642844

H,0,2.1872557897,0.9603587263,1.4054813929
O,0,0.9064583883,1.9921569134,0.2401302642
C,0,0.3891480665,-0.2667311186,0.9389207507
H,0,1.0370538391,-1.1186473291,0.6823119233
H,0,0.1417840172,-0.3920092602,2.0045025001
C,0,-0.8744429973,-0.2503925739,0.0743337715
H,0,-1.4285888118,0.6739309101,0.2750578119
H,0,-1.5166614452,-1.0866576842,0.3785120255
C,0,-0.5730109955,-0.3550163036,-1.4257020196
H,0,0.0234146404,0.497685911,-1.7640689426
H,0,-1.5006912298,-0.3734686182,-2.0089262329
H,0,-0.0185445484,-1.2766020575,-1.6456165294

butanal, conformer 3
H CBS-APNO -232.33066 H_{rel} = +0.61 kcal/mol
C,0,1.2284052875,0.9503716431,0.7804293347
H,0,0.6780107093,1.8991828816,0.9782459646
O,0,2.3871022408,0.97016185,0.4377390715
C,0,0.399174969,-0.3044162704,0.9686276312
H,0,1.0243239723,-1.1708827471,0.7257056256
H,0,0.1218585862,-0.3606284471,2.0314197207
C,0,-0.8731855128,-0.2725369697,0.104046835
H,0,-1.4556268097,0.6265237321,0.3506589826
H,0,-1.5011066847,-1.1325869033,0.3662490279
C,0,-0.5633012545,-0.2953306485,-1.3975905774
H,0,0.0338377887,0.5748974633,-1.6962515937
H,0,-1.4857581635,-0.2898434487,-1.9888297529
H,0,0.0062638715,-1.1949121354,-1.6604492696

butanal, conformer 4
H CBS-APNO -232.33041 H_{rel} = +0.77 kcal/mol
C,0,0.3284126088,-0.008041745,2.06033596
H,0,-0.2505568289,-0.9024524568,2.3850562017
O,0,0.6287168251,0.852604087,2.8541111628
C,0,0.6651169,0.0266901939,0.5838020053
H,0,1.2667911147,0.9210812816,0.3881297005
H,0,1.2722398442,-0.8611069795,0.3513502343
C,0,-0.6047409586,0.0119266985,-0.2834946892
H,0,-1.2079301386,0.899009925,-0.0486321443
H,0,-1.2139980169,-0.8642600608,-0.0212295682
C,0,-0.2842676776,-0.0140343021,-1.7815728557
H,0,0.3106318927,0.8621721696,-2.0658329997
H,0,-1.2011882253,-0.0120180254,-2.3815642445

H,0,0.2907736605,-0.9115697861,-2.0404587631

butanal, conformer 5
H CBS-APNO -232.32866 H_{rel} = +1.86 kcal/mol
C,0,1.2819010177,-0.9109837661,0.738186279
H,0,2.0311163703,-0.8315256217,-0.0792637182
O,0,1.1661048801,-1.9392652368,1.3652867722
C,0,0.4141931334,0.3119663149,0.9777703012
H,0,0.1541363716,0.3326699444,2.0418609005
H,0,0.9824619866,1.2189515551,0.730749727
C,0,-0.8714050636,0.2540703461,0.1276447146
H,0,-1.50568897,1.1069633068,0.3992014388
H,0,-1.4244723826,-0.6545029395,0.398082119
C,0,-0.6060952158,0.2729323276,-1.3833357995
H,0,-0.0267484689,1.1605817645,-1.6669938654
H,0,-1.5482290551,0.2915176517,-1.942244377
H,0,-0.0472756037,-0.613372647,-1.7069434923

2-butanone, Cs, global minimum
H CBS-APNO 232.34358
C,0,-0.4689018862,0.0361562644,-0.6506414833
O,0,-1.6785882157,0.0534629108,-0.5648247232
C,0,0.4307487219,0.0781808073,0.5814196941
H,0,1.1759364588,-0.7241985134,0.4859077381
H,0,0.9989618088,1.0189847457,0.5363101903
C,0,-0.3412079011,-0.0315615284,1.8952474071
H,0,-0.8908092217,-0.9771481026,1.9421699173
H,0,-1.0716161134,0.7783174602,1.98004955
H,0,0.3425983583,0.0184726199,2.7496074904
C,0,0.2407850637,-0.0392526131,-1.9949953397
H,0,0.6659696342,-1.0431835116,-2.1206326173
H,0,1.06812458,0.6782282259,-2.0397475495
H,0,-0.472002288,0.1535412351,-2.7998702713

2-butanone, conformer 2
H CBS-APNO -232.34151 H_{rel} = +1.30 kcal/mol
C,0,-0.4404037234,-0.6655387155,-0.5323201921
O,0,-0.5503199564,-1.8735320874,-0.5051702386
C,0,-0.5545563911,0.1630898299,0.7441908909
H,0,-0.8308746532,1.1976078067,0.5065666609
H,0,-1.3464691644,-0.2792522915,1.3579352757
C,0,0.7788078829,0.1310203019,1.5116923079
H,0,1.5906955843,0.5608253196,0.9123046977

H,0,1.0432799443,-0.9026085332,1.7578176942
H,0,0.7052066746,0.7034161445,2.4423590175
C,0,-0.1464626004,0.093328699,-1.8167562008
H,0,0.6937613225,0.7825215646,-1.6686646306
H,0,-1.0212004998,0.6981717781,-2.087665785
H,0,0.07853558,-0.6090508166,-2.6222894977

tetrahydrofuran
H CBS-APNO -232.32216
O,0,0.,0.,1.4880704065
C,0,-1.160356441,0.1373782618,0.6648477645
C,0,-0.7277320817,-0.2420323965,-0.7551302541
C,0,0.7277320817,0.2420323965,-0.7551302541
C,0,1.160356441,-0.1373782618,0.6648477645
H,0,-1.5098183669,1.1803142865,0.6961914559
H,0,-1.951376585,-0.5078449613,1.0621202854
H,0,-0.7625783077,-1.3306870298,-0.8859560904
H,0,-1.3486927508,0.2247321021,-1.5261088656
H,0,0.7625783077,1.3306870298,-0.8859560904
H,0,1.3486927508,-0.2247321021,-1.5261088656
H,0,1.951376585,0.5078449613,1.0621202854
H,0,1.5098183669,-1.1803142865,0.6961914559

Five carbons
cyclopentane
H CBS-APNO -196.40232
H,0,-0.1534814618,-1.3354102032,-1.8431391701
C,0,0.2574373909,-0.7239414084,-1.0319015997
C,0,-0.2562134737,0.7233689173,-1.032695165
C,0,0.1437504547,1.2330116852,0.3650915839
C,0,0.0013526572,-0.0002882499,1.3022227904
C,0,-0.1463425167,-1.2325361663,0.3649507483
H,0,1.3521411293,-0.7229519183,-1.1310789672
H,0,-1.3506260409,0.7220471135,-1.134847042
H,0,0.1566216852,1.334724327,-1.8430405082
H,0,1.1895955157,1.5655414108,0.3420081319
H,0,-0.4619685665,2.0836275616,0.6963121503
H,0,-0.8715700104,0.0968872658,1.957577527
H,0,0.8787739126,-0.0982728879,1.951456509
H,0,-1.1939758638,-1.5594061074,0.3403810148
H,0,0.4545051884,-2.0864013401,0.6967009881

cyclopentanol, global minimum

H CBS-APNO -271.62012
C,0,-0.0035671084,-0.0637930541,-1.1479268336
C,0,-1.2854092767,0.1042810486,-0.3155034369
C,0,-0.8290287288,0.1509520777,1.172697261
C,0,0.7050984276,-0.0415609434,1.1427401253
C,0,1.1026651923,0.4927234453,-0.2421675341
H,0,-0.0636085425,0.4549765561,-2.1154152704
H,0,-1.9574015534,-0.7329662633,-0.5266286968
H,0,-1.8005714837,1.0298563622,-0.5974902274
H,0,-1.0751619123,1.1266164245,1.609760877
H,0,-1.3262434236,-0.6122689276,1.7799346418
H,0,0.9482715239,-1.1079444515,1.1989405801
H,0,1.2118524463,0.4713686098,1.9672183565
H,0,2.1054435492,0.1863403266,-0.566375166
H,0,1.0634467965,1.5914002685,-0.2520901663
O,0,0.1710428601,-1.4668140318,-1.3519112852
H,0,1.0331712335,-1.5831664477,-1.7557842249

cyclopentanol, conformer 2
H CBS-APNO -271.61882 H_{rel} = +0.81 kcal/mol
C,0,0.4322144703,0.,-1.1428319079
C,0,0.4903169807,1.1977082395,-0.1853435287
C,0,-0.4124132278,0.7806822562,1.0030112452
C,0,-0.4124132278,-0.7806822562,1.0030112452
C,0,0.4903169807,-1.1977082395,-0.1853435287
H,0,1.2478137607,0.,-1.8716546779
H,0,0.1644618251,2.1218137325,-0.6730575848
H,0,1.5244886128,1.3338682297,0.1570087415
H,0,-0.0464163923,1.1902394239,1.949851297
H,0,-1.430694678,1.1659321273,0.8673666178
H,0,-1.430694678,-1.1659321273,0.8673666178
H,0,-0.0464163923,-1.1902394239,1.949851297
H,0,0.1644618251,-2.1218137325,-0.6730575848
H,0,1.5244886128,-1.3338682297,0.1570087415
O,0,-0.7624264011,0.,-1.9200086188
H,0,-1.4970880184,0.,-1.3031783382

cyclopentanol, conformer 3
H CBS-APNO -271.61844 H_{rel} = +1.05 kcal/mol
C,0,0.1236714258,-0.6416886559,-0.8604404646
C,0,1.2113330065,0.2569416524,-0.2784500463
C,0,0.7881853846,0.4017009241,1.1930116445
C,0,-0.7704186215,0.4067588497,1.1679719741

C,0,-1.1654441666,-0.0590110306,-0.2579452121
H,0,0.2731789544,-1.670398257,-0.4890444915
H,0,1.1611481323,1.2233205064,-0.7979812572
H,0,2.2188344633,-0.1518413404,-0.4068966424
H,0,1.1488862153,-0.4624175462,1.7652849257
H,0,1.2014902053,1.2989772068,1.6648914929
H,0,-1.1690854231,1.4063768522,1.3717878902
H,0,-1.1751834112,-0.2612802614,1.9362674684
H,0,-1.4547940917,0.7965748057,-0.8813681299
H,0,-1.9971471107,-0.7739674656,-0.2607067724
O,0,0.1682802624,-0.6170683159,-2.2773468692
H,0,-0.5629332254,-1.1529769242,-2.5890385104

cyclopentanol, conformer 3
H CBS-APNO -271.61829 H_{rel} = +1.15 kcal/mol
C,0,-0.4072318333,0.,-1.0456909909
C,0,0.1243241209,-1.1973775424,-0.2467436023
C,0,-0.0751794506,-0.781870544,1.22607765
C,0,-0.0751794506,0.781870544,1.22607765
C,0,0.1243241209,1.1973775424,-0.2467436023
H,0,-1.5048619103,0.,-1.0178472909
H,0,1.1964069999,-1.3065857421,-0.4712086364
H,0,-0.3701735878,-2.1383812802,-0.5084294489
H,0,-1.0339630978,-1.1598495365,1.5997530638
H,0,0.7058491669,-1.1928583357,1.874113458
H,0,0.7058491669,1.1928583357,1.874113458
H,0,-1.0339630978,1.1598495365,1.5997530638
H,0,1.1964069999,1.3065857421,-0.4712086364
H,0,-0.3701735878,2.1383812802,-0.5084294489
O,0,-0.0705544169,0.,-2.4200576014
H,0,0.8881207973,0.,-2.4635270858

Radicals
No carbon
H*
H CBS-QB3 -0.49746
H CBS-APNO -0.49759

HO*
H CBS-QB3 -75.64642
H CBS-APNO -75.71976

O,0,0.,0.,-0.1111997427
H,0,0.,0.,0.8581997427

One carbon
*CHO
H CBS-QB3 -113.70096
H CBS-APNO -113.83157
C,0,0.5579686982,-0.1575938368,0.
H,0,1.3330306383,0.6587756743,0.
O,0,-0.6079993365,0.0268181625,0.

HOC*H_2
H CBS-QB3 -114.88385
H CBS-APNO -115.01509
C,0,0.713667869,0.0131204643,0.2032832655
H,0,1.0930774552,0.9621972632,0.1698308116
H,0,1.2760766192,-0.8964677527,-0.0294805931
O,0,-0.6417118364,-0.1901773655,-0.1998750282
H,0,-1.0631101071,0.6653273907,-0.2901919249

*CH_3
H CBS-QB3 -39.74079
H CBS-APNO -39.79954
H,0,1.0829151208,0.,-0.1517651767
C,0,0.0002474032,0.,-0.1517046496
H,0,-0.5410812418,0.9376197917,-0.1517650869
H,0,-0.5410812418,-0.9376197917,-0.1517650869

Two carbons
*$COCH_2OH$
H CBS-QB3 -228.07255
H CBS-APNO -228.33350
C,0,0.9775608134,-0.2753336506,-0.0907034419
O,0,0.6809039311,-1.4187641148,-0.002777335
C,0,-0.0056120839,0.9087886842,-0.0493356251
H,0,0.1690164571,1.3940504172,0.9239181149
H,0,0.2709067485,1.6145814837,-0.8355440802
O,0,-1.3435298275,0.5307859515,-0.2529469785
H,0,-1.5074840387,-0.2306797711,0.3073893459

$CHOCH_2O$*
H CBS-QB3 -228.04054
H CBS-APNO -228.30173

C,0,-0.6191393578,0.6093216357,0.0003891257
H,0,-0.9212580894,1.219461433,-0.8718311489
H,0,-0.9207888036,1.2150934017,0.8758005528
C,0,0.9019060534,0.4503105973,-0.0001168088
H,0,1.4560820022,1.4165094913,0.0031068672
O,0,1.4723607618,-0.609370073,-0.003399825
O,0,-1.3374165666,-0.558370486,-0.0038797631

CHOC*HOH
H CBS-QB3 -228.09247
H CBS-APNO -228.35323
C,0,0.9205259426,-0.2812437267,0.0490714155
H,0,1.9951349693,-0.0641043642,0.148145411
O,0,0.4987835959,-1.4046253891,-0.2608031436
C,0,-0.0039406376,0.7822034439,0.2901921919
H,0,0.251781195,1.7978107339,0.5653890826
O,0,-1.3082733575,0.5211964148,0.1706056393
H,0,-1.3393037077,-0.4187661126,-0.0759775968

$CHOCH_2$*
H CBS-QB3 -152.92835
H CBS-APNO -153.11611
C,0,-1.0821091381,-0.2240992075,-0.1008084436
H,0,-1.9258079087,0.3347007651,-0.4943772253
H,0,-1.2165459873,-1.2533483092,0.2172851269
C,0,0.2239164295,0.3741773791,0.010142199
H,0,0.3171510219,1.4242436495,-0.3249675246
O,0,1.2003955828,-0.2316742771,0.4517258676

*$COCH_3$
H CBS-QB3 -152.93713
H CBS-APNO -153.12623
C,0,-0.2463357599,-0.4482320964,0.
O,0,-1.2658902816,0.1541643738,0.
C,0,1.171620188,0.1160295784,0.
H,0,1.1468106164,1.2118139768,0.
H,0,1.6938976185,-0.2578879162,-0.8855557698
H,0,1.6938976185,-0.2578879162,0.8855557698

*CH(OH)CH_2OH
H CBS-QB3 -229.25644
H CBS-APNO -229.51793
C,0,0.7339975333,0.569264567,-0.2807820144

H,0,1.3436095059,1.463628341,-0.3697290656
O,0,1.4422027471,-0.5231403602,0.1370641844
H,0,0.7843331056,-1.2138633862,0.2758345632
C,0,-0.6886940414,0.6415254178,0.1849980943
H,0,-1.2118986107,1.4765493615,-0.2996647989
H,0,-0.7478095361,0.7870036323,1.2738504946
O,0,-1.342114955,-0.6101506627,-0.0447589318
H,0,-1.3626257486,-0.7268169104,-0.9978125264

Three carbons
*COCH(OH)CH_2OH, glyceraldehyde loss of C1-H
H CBS-QB3 -342.44352
H CBS-APNO -342.83466
C,0,-1.1087596843,0.6707123304,-0.4267053786
C,0,-0.0538927809,-0.4393116724,-0.6170843147
H,0,-0.2537682243,-0.8692343102,-1.6109137459
C,0,1.3484794992,0.1530677086,-0.5610416551
H,0,1.4550062126,0.9283006418,-1.3248134709
H,0,2.0723081371,-0.6497406467,-0.7624049308
O,0,-0.1388451617,-1.414147912,0.405997703
H,0,-1.0509783348,-1.4343272386,0.7054122554
O,0,1.5715305562,0.7603956088,0.6933763628
H,0,1.3787475019,0.077947037,1.3413502025
O,0,-2.0228277211,0.619338453,0.3248269725

CHOC*(OH)CH_2OH, glyceraldehyde loss of C2-H
H CBS-QB3 -342.46299
H CBS-APNO -342.85378
C,0,1.2684977561,0.7471545571,-0.044051494
H,0,1.1930432346,1.845722127,-0.0424937869
O,0,2.339507729,0.1581872176,-0.24921137
C,0,0.0774050756,-0.0056289759,0.2034768645
O,0,0.1890946313,-1.3429403781,0.1770140532
H,0,1.1261396366,-1.504248859,-0.0270363439
C,0,-1.3022462618,0.5119032718,0.4569075785
H,0,-1.635588416,0.1776896688,1.451735422
H,0,-1.288640374,1.6054568239,0.447630132
O,0,-2.2141630419,0.1012996221,-0.5505004477
H,0,-2.1830499694,-0.8575950753,-0.5644706077

CHOC*HCH_2OH, glyceraldehyde loss of C2-OH
H CBS-QB3 -267.29833
H CBS-APNO -267.61590

C,0,1.3350095025,0.3748683692,0.0469813022
H,0,2.2735101148,0.9295614338,-0.1312596912
C,0,0.1025199613,1.0969987294,-0.1542340566
H,0,0.1566161449,2.1568625154,-0.39495348
O,0,1.3716813372,-0.802850274,0.4163690168
C,0,-1.2357175013,0.4498866249,0.0930558125
H,0,-2.0202322819,1.0325819384,-0.3995605993
H,0,-1.4366244863,0.4890180127,1.1797442831
O,0,-1.330756283,-0.8693149413,-0.4003620467
H,0,-0.5790065081,-1.3396124085,-0.0297805408

CHOCH(O*)CH_2OH, glyceraldehyde loss of H from C2OH
H CBS-QB3 -342.41525
H CBS-APNO -342.80501
C,0,1.6440358174,-0.0362942273,-0.3798948316
H,0,2.164322804,-0.2963042404,-1.3238027893
O,0,2.2048409612,0.4582465569,0.56525878
C,0,0.1425240275,-0.3615833277,-0.3831088689
H,0,0.0699495427,-1.456346135,-0.2069312455
C,0,-0.6705035408,0.3528504736,0.7025158905
H,0,-0.5813893028,1.4389129681,0.5517888619
H,0,-0.2688953208,0.1132826967,1.6892339391
O,0,-0.3902340145,-0.1856653827,-1.6385694986
O,0,-2.0133316683,-0.0779748097,0.6652654874
H,0,-2.3013183054,0.0508764274,-0.241754725

CHOCH(OH)C*HOH, glyceraldehyde loss of C3-H
H CBS-QB3 -342.43859
H CBS-APNO -342.82941
C,0,1.1840429172,0.6595421423,-0.1873010755
H,0,1.2805190577,1.6918661152,-0.5734316027
O,0,1.9290796191,0.2157167602,0.657082611
C,0,0.0341552496,-0.1735892788,-0.760769862
H,0,0.1927010519,-0.2517881609,-1.8477030143
C,0,-1.2840995621,0.5064848592,-0.4850641409
H,0,-1.5118756427,1.4705439249,-0.9310530097
O,0,0.0255811365,-1.44067036,-0.1381044308
H,0,0.8504270637,-1.4776705926,0.3617506911
O,0,-1.7724481574,0.356230945,0.7773846914
H,0,-1.4720827333,-0.5186663546,1.0542091422

CHOCH(OH)C^*H_2, glyceraldehyde loss of C3-OH
H CBS-QB3 -267.28590

H CBS-APNO -267.60453
C,0,0.825468393,0.6751749791,-0.2289307318
H,0,0.8536564388,1.735526554,-0.5437790706
O,0,1.7916895935,0.116113985,0.2360777869
C,0,-0.5151162933,-0.0501472538,-0.4048290932
H,0,-0.730655646,-0.0280194592,-1.488773305
O,0,-0.4082473061,-1.3726204106,0.0545849612
H,0,0.5209663863,-1.4843638897,0.2858297148
C,0,-1.5810107435,0.6990261861,0.3343058953
H,0,-1.8335723365,0.3798460821,1.3393205421
H,0,-2.0141784863,1.6054632269,-0.0768066997

CHOCH(OH)CH_2O*, glyceraldehyde loss of H from C3OH
H CBS-QB3 -342.41611
H CBS-APNO -342.80598
C,0,-1.5404282626,0.0005897947,0.0283375475
H,0,-2.158816298,-0.1914918274,-0.8713245979
O,0,-1.9766592855,-0.0938333594,1.1516348638
C,0,-0.1008931102,0.3967635823,-0.2666782151
H,0,-0.021724622,1.4882094289,-0.1263617683
C,0,0.8761885886,-0.2884457015,0.6985559299
H,0,0.7483942847,-1.3852363792,0.6115449953
H,0,0.6873104593,-0.0233643577,1.747487084
O,0,0.1744190795,0.0319941733,-1.6002124815
H,0,1.1244599984,0.129584214,-1.7055095485
O,0,2.1877491677,-0.0647695681,0.3325261908

*CH(OH)$COCH_2OH$, dihydroxyacetone loss of C1-H
H CBS-QB3 -342.46600
H CBS-APNO -342.85659
C,0,-0.247654225,0.0276234018,0.1366398522
O,0,-1.4790883693,0.1774446616,0.164888695
C,0,0.4912904217,0.028944866,-1.1950337392
H,0,0.9420120073,1.0277265297,-1.3220203253
H,0,1.3053396714,-0.7048863816,-1.1781440354
C,0,0.4657529297,-0.0996527057,1.3743542267
H,0,1.5327656232,-0.2499529845,1.4825047934
O,0,-0.3818823498,-0.2845186993,-2.249379208
H,0,-1.2479496981,0.0113123362,-1.9509431132
O,0,-0.2303479842,-0.0310117259,2.5120895326
H,0,-1.1502380278,0.0969707017,2.2250433205

*CH_2COCH_2OH, dihydroxyacetone loss of C1-OH

H CBS-QB3 -267.30318
H CBS-APNO -267.62156
C,0,-0.446089645,-0.0104371073,0.3341415238
O,0,-1.6659238174,0.0662621852,0.1909704736
C,0,0.4592830056,0.0656744961,-0.8951094427
H,0,1.1532412082,0.9088478804,-0.7584104423
H,0,1.0620386272,-0.854307512,-0.9346609338
C,0,0.1663765582,-0.1717407873,1.6377516881
H,0,-0.4738737195,-0.2261345756,2.5117032439
H,0,1.2443628237,-0.2383395883,1.7525351466
O,0,-0.2911924249,0.2218458713,-2.0678983057
H,0,-1.2082236161,0.2383291375,-1.7710249514

$CH_2(O^*)COCH_2OH$, dihydroxyacetone loss of H from C1OH
H CBS-QB3 -342.42062
H CBS-APNO -342.81228
C,0,0.2937009861,0.0950677206,-0.4123537195
O,0,0.8844951243,-0.1039479305,-1.4537166448
C,0,-0.5096301954,-1.0079745985,0.2578435479
H,0,-0.1137604588,-1.1474758022,1.274191306
H,0,-1.5453577425,-0.6554569243,0.3680039655
C,0,0.3390548799,1.4662341476,0.2604332463
H,0,-0.0264751353,2.2285034526,-0.4504706574
H,0,1.3924131607,1.7409363518,0.447578894
O,0,-0.4499354222,-2.1970145442,-0.4820625488
H,0,0.1026203191,-1.9931959398,-1.2442792788
O,0,-0.3671265112,1.5743240654,1.4348308925

$^*CH(OH)CH(OH)CH_2OH$, erythrose or threose loss of formyl
H CBS-QB3 -343.63104
C,0,-0.5130148534,-0.0905397586,-1.3381631753
C,0,0.1373810689,-0.7188179877,-0.1631263777
H,0,-0.0049941965,-1.8047648347,-0.2200138277
C,0,-0.3976362494,-0.195811893,1.1732190166
H,0,-1.4818098895,-0.2956582245,1.2280379174
H,0,0.055053134,-0.7561342949,1.9984105054
O,0,-0.7963050224,1.2376196843,-1.3656730788
H,0,-0.6458324616,1.5895375158,-0.4671306433
O,0,1.5751851871,-0.4338532656,-0.0656150476
H,0,1.9217247757,-0.4054374252,-0.9635994835
O,0,-0.099283438,1.2018574464,1.2982373227
H,0,0.8597265959,1.2574716885,1.1804757766
H,0,-0.6101936508,-0.5854676506,-2.2950599048

Four carbons
Erythrose, loss of C1-H
H CBS-QB3 -456.81471
C,0,0.2279618375,0.9845671992,1.9187759481
O,0,-0.3488400055,1.9672851171,2.2280700221
C,0,0.7549173115,0.6616790094,0.5120997651
H,0,1.8455371433,0.7831714236,0.5870589353
C,0,0.4839966307,-0.8045656322,0.1214564471
H,0,0.755469631,-1.4150093928,0.9883254697
C,0,-0.9884153545,-1.0513443013,-0.2454722062
H,0,-1.1697671599,-2.1263436082,-0.2869116717
H,0,-1.6580738019,-0.6177242763,0.5091444609
O,0,0.2051547709,1.5333746569,-0.4699829839
H,0,0.1571871915,2.4229221167,-0.1009558023
O,0,1.318079956,-1.1895102623,-0.9514232081
H,0,0.8103191628,-1.001689562,-1.7543062348
O,0,-1.2660030696,-0.5514778075,-1.5495528689
H,0,-1.1275262437,0.4046643199,-1.5063250724

Threose, loss of C1-H
H CBS-QB3 -456.81533
C,0,-0.8998075518,-1.3074992978,1.7857278348
O,0,-1.4822050934,-0.8137358182,2.6841844685
C,0,-0.7728776744,-0.7291497926,0.3701721652
C,0,0.7092071097,-0.3877595342,0.0969491191
H,0,1.296499031,-1.3014059292,0.2380558518
C,0,0.9104934268,0.1413250049,-1.3312035617
H,0,1.9785971391,0.1734981299,-1.5538527846
H,0,0.4258895219,-0.5184311982,-2.0624079237
O,0,1.159286855,0.5786357172,1.0313149534
H,0,1.0826374838,1.4307566475,0.577458111
O,0,0.4458230229,1.4834215227,-1.4367962906
H,0,-0.502003464,1.4597838465,-1.2420050114
H,0,-1.1026329486,-1.5084629468,-0.3233558329
O,0,-1.6279485841,0.3944275918,0.172860453
H,0,-1.6209582738,0.9045960565,0.992897448

Erythrose or Threose, loss of C2-H
H CBS-QB3 -456.83511
C,0,-1.7379685036,-0.6371098016,-0.9677434854
H,0,-1.9553517129,0.227773447,-1.6022952825

O,0,-2.2731253297,-1.7505822998,-1.1353968895
C,0,-0.806368028,-0.4856548258,0.0999422644
C,0,-0.0640366818,0.7593852044,0.4979358387
H,0,-0.4945222919,1.1302019135,1.4377747398
C,0,1.4341998096,0.4961033153,0.7261965388
H,0,1.8857956465,1.4010592856,1.1345096345
H,0,1.5814579334,-0.3289058935,1.4287537127
O,0,-0.59951455,-1.5747810354,0.8466700629
H,0,-1.1932211225,-2.249672832,0.4441425707
O,0,-0.2353318099,1.7765011741,-0.4719950177
H,0,0.4586725966,1.6247957382,-1.1297039132
O,0,2.0840885549,0.2608947934,-0.5227213752
H,0,1.9152284893,-0.6500081833,-0.7860683991

Erythrose or Threose, loss of C2-OH
H CBS-QB3 -381.67105
C,0,-0.22769674,-0.7891117913,1.9777748214
H,0,-0.4229970619,-1.7434516314,2.4945978133
O,0,-0.427714782,0.2817119408,2.5755982166
C,0,0.2598629329,-0.8711427618,0.6436667407
C,0,0.5979219281,0.2990129165,-0.2177484302
H,0,1.7059788048,0.3227099286,-0.2807628373
C,0,0.0722883546,0.1361691019,-1.6564335657
H,0,0.4940512667,0.953110828,-2.2569669571
H,0,0.4033523261,-0.8136716268,-2.0827257883
O,0,0.1065423315,1.5435762628,0.2439818823
H,0,-0.0758005531,1.4572894161,1.1958402236
O,0,-1.3393468741,0.1446319419,-1.6789690036
H,0,-1.5990632435,0.9370264552,-1.1916012373
H,0,0.4526203101,-1.8578619804,0.2337481217

Erythrose, loss of H from C2OH
H CBS-QB3 -456.78988
C,0,1.0832256591,-1.0336989927,1.6200879553
H,0,0.85479822,-1.8174066664,2.3679337417
O,0,2.1477791034,-0.4737273278,1.5726877119
C,0,-0.0758776524,-0.7915582196,0.6287782568
H,0,-0.8101615149,-0.1805918947,1.2090935326
C,0,0.2733593685,0.0567293324,-0.6235254965
H,0,0.8842813221,-0.5577599693,-1.2987408038
C,0,-1.0099603932,0.4880988235,-1.3225161678
H,0,-0.7532095551,0.9930098855,-2.262076231
H,0,-1.6248444017,-0.3841755673,-1.5464098063

O,0,-0.6838767637,-1.9675139164,0.3634243537
O,0,0.940434353,1.2583334424,-0.2590981952
H,0,1.7268321756,1.0169423228,0.2478156852
O,0,-1.7715290892,1.3351602408,-0.4722106102
H,0,-1.1812488315,2.0581575067,-0.2252439265

Threose, loss of H from C2OH
H CBS-QB3 -456.79164
C,0,-1.8975289756,-1.0273162308,0.5137121331
H,0,-1.480095027,-1.992581054,0.8555772237
O,0,-3.0124776474,-0.8786238593,0.1083859166
C,0,-0.8920885464,0.1513100173,0.6248973039
C,0,0.0550320325,-0.0437502939,-0.7092824242
H,0,-0.6142295211,0.1352858178,-1.5546756038
C,0,1.1891620306,0.9840853308,-0.6051536295
H,0,1.722500853,1.006061782,-1.5589958683
H,0,0.7927744408,1.9839434303,-0.3975195864
O,0,0.5575485587,-1.3301847238,-0.7997709978
H,0,1.3541988451,-1.3435628592,-0.2442563572
O,0,2.1228319431,0.5570156173,0.3693783465
H,0,1.6767190601,0.6149742762,1.2312877434
H,0,-1.4271699332,1.1052104509,0.4576673052
O,0,-0.1471781132,0.0781332984,1.7087494951

Erythrose or Threose, loss of C3-H
H CBS-QB3 -456.81283
C,0,-1.1376261687,1.1814397031,0.3342096466
H,0,-0.5970265786,1.5080077438,1.2361953243
O,0,-2.3456797886,1.1650454201,0.2662330879
C,0,-0.2864662896,0.667155492,-0.8337779852
H,0,0.4049224821,1.469523658,-1.1312879139
C,0,0.4997365551,-0.5492152408,-0.4007335711
C,0,1.5757577634,-0.5026816695,0.6159423382
H,0,2.1760543018,-1.4169815263,0.5479547818
H,0,2.2297259493,0.3524393872,0.433601919
O,0,-1.1469606608,0.2987469298,-1.8959320227
H,0,-2.0360036833,0.5699816663,-1.6067626218
O,0,-0.155976553,-1.7361006112,-0.5182301005
H,0,-0.8337150045,-1.5918494003,-1.2014763187
O,0,1.0922022355,-0.3201318166,1.9701303882
H,0,0.5610564398,-1.0953777357,2.183932048

Erythrose or Threose, loss of C3-OH

H CBS-QB3 -381.65521
C,0,-1.440038753,0.1454248471,-1.2616394566
H,0,-2.1411705434,0.9949743573,-1.1763237229
O,0,-1.0743518318,-0.2837363612,-2.3290353867
C,0,-0.9093842238,-0.4366540195,0.0516807554
C,0,-0.1395265495,0.6411358073,0.7549204589
H,0,-0.6464004367,1.2479497532,1.4962643001
C,0,1.3397713071,0.7301906425,0.5684187918
H,0,1.5971127503,0.6938181125,-0.5006398968
H,0,1.7225914441,1.6686378716,0.9712180101
O,0,2.0142025934,-0.3162882374,1.2793116322
H,0,1.6493827314,-1.1378595241,0.9246301442
H,0,-1.7810230281,-0.7363485638,0.6537911061
O,0,-0.1005111536,-1.5641299553,-0.2321842094
H,0,-0.0906543065,-1.6471157301,-1.2004125264

Erythrose, loss of H from C3OH
H CBS-QB3 -456.78965
C,0,2.0213514437,0.310361491,0.5800495343
H,0,2.3673240515,1.3330233805,0.345534234
O,0,2.781321109,-0.6146832913,0.7380472976
C,0,0.5227692947,0.1137652229,0.6603542811
H,0,0.1248113877,0.7929192697,1.4265670768
C,0,-0.1175518132,0.5567866246,-0.6961918247
H,0,0.2547276503,-0.0624824614,-1.5312371026
C,0,-1.7022878375,0.1757971806,-0.6357288201
H,0,-2.0777447246,0.5863181944,0.3084018506
H,0,-2.177443151,0.6726994163,-1.4791965153
O,0,0.2272663793,-1.227464854,0.9831628157
H,0,1.0793552751,-1.6957877605,1.0019442729
O,0,-0.0351002411,1.8653096814,-0.895590788
O,0,-1.8803463773,-1.1920071621,-0.7640991209
H,0,-1.3884524467,-1.6145559321,-0.0420171914

Threose, loss of H from C3OH
H CBS-QB3 -456.78882
C,0,1.5303279756,0.9946523127,0.4113184033
H,0,1.8026852862,0.5986060244,1.4078486512
O,0,2.3560655772,1.267409274,-0.4253160578
C,0,0.0483637075,1.1834294402,0.1500399396
C,0,-0.7213383829,-0.1352445283,0.4388090114
H,0,-1.8048374702,0.092687933,0.336815542
C,0,-0.4515570359,-1.2870101073,-0.5689158404

H,0,-0.7436080891,-0.9762544757,-1.5706101372
H,0,0.6291711289,-1.4970408205,-0.5713528136
O,0,-0.5782168315,-0.5538164979,1.7201973471
O,0,-1.2058301262,-2.4205299695,-0.2207002534
H,0,-1.0655646684,-2.5634126886,0.7240754679
H,0,-0.3066751246,1.9208387956,0.8874357286
O,0,-0.1822380019,1.6185432103,-1.1620162507
H,0,0.6932530553,1.7571410976,-1.557629738

Erythrose, loss of C4-H
H CBS-QB3 -456.81080
C,0,1.1325925431,0.3106196204,1.0591131664
H,0,1.3293874719,1.3380711909,0.712513732
O,0,2.0010923498,-0.3779213257,1.5442700927
C,0,-0.2867346316,-0.2137934559,0.9311143702
H,0,-0.9265564474,0.4186658287,1.5664095324
C,0,-0.8421774599,-0.1445851959,-0.5294910681
H,0,-1.9018146802,-0.4259737871,-0.4682318741
C,0,-0.7080393365,1.183643861,-1.1887901066
H,0,-1.2800671479,2.0481065018,-0.8808066214
O,0,-0.3297743641,-1.5631162762,1.3484715749
H,0,0.5355819781,-1.7344210742,1.7581396502
O,0,-0.1102143832,-1.0761391612,-1.3220749373
H,0,0.0055930192,-1.8595843079,-0.7662602479
O,0,0.4949337842,1.474675081,-1.7578226046
H,0,0.8861983046,0.6217525003,-2.0065556588

Threose, loss of C4-H
H CBS-QB3 -456.81108
C,0,0.5341444695,-1.1330733969,-1.5924382054
H,0,1.3606630415,-0.672926203,-2.1651615551
O,0,0.3420049491,-2.3232157264,-1.5893044052
C,0,-0.3613129349,-0.1877982647,-0.8080609625
C,0,0.4854810939,0.6574784558,0.1703058061
H,0,1.1034380418,1.3441746083,-0.4260454403
C,0,-0.3331702728,1.471290886,1.103318956
H,0,-0.0047444268,2.4377678645,1.4626514545
O,0,1.3321639341,-0.316977244,0.8325421646
H,0,1.6856997933,0.0949324947,1.6278665697
O,0,-1.3027258717,0.8976474289,1.8562565112
H,0,-1.528016484,0.0459992288,1.4369340031
H,0,-0.8456964029,0.494346973,-1.5196766395
O,0,-1.3545293485,-0.9382744381,-0.1311621316

H,0,-1.1133995815,-1.871372667,-0.2580241257

Erythrose, loss of C4-OH
H CBS-QB3 -381.65859
C,0,0.1274935373,0.6935809514,1.4280355302
H,0,1.1928291517,0.9782412507,1.3573228005
O,0,-0.4234581738,0.5288395385,2.4893885494
C,0,-0.6256514233,0.5345398819,0.1200745153
H,0,-0.6651518889,1.5269051892,-0.3589592858
C,0,0.0977275778,-0.453408306,-0.8389592558
H,0,-0.5451249271,-0.496744137,-1.7369325849
C,0,1.4610321685,-0.0204619616,-1.2252404429
H,0,2.3080768431,-0.645433302,-0.9791299155
H,0,1.6037002149,0.8648994448,-1.8310885881
O,0,-1.9210556192,0.0376498775,0.371811046
H,0,-2.0397290575,0.0933650875,1.3342662671
O,0,0.162768898,-1.726587834,-0.2186539065
H,0,-0.7334573015,-1.9153856811,0.0880632712

Threose, loss of C4-OH
H CBS-QB3 -381.65735
C,0,-0.3600443527,-0.163338043,-1.843308066
H,0,-1.4158699862,0.1047501184,-2.0395231668
O,0,0.4794640476,-0.1167556632,-2.7082085247
C,0,-0.0217955295,-0.619269396,-0.4353247895
C,0,-0.4976193797,0.408863034,0.6048805933
H,0,-1.5629642413,0.60892944,0.4500258913
C,0,-0.2500105179,-0.0418245656,2.0032183004
H,0,-0.7455969589,0.4675648517,2.8198156032
H,0,0.5835389401,-0.6967736392,2.2235727475
O,0,0.1525545367,1.6659211278,0.338576871
H,0,1.0997893311,1.4781943414,0.3810022729
H,0,-0.5586354501,-1.5693206362,-0.2691534662
O,0,1.3719549982,-0.7984975791,-0.311987401
H,0,1.7252355624,-0.728445391,-1.2135878655

Erythrose, loss of H from C4OH
H CBS-QB3 -456.79218
C,0,-1.9753114315,-0.3749963518,-0.4217610736
H,0,-1.9322273527,-0.7815144325,-1.445649275
O,0,-2.9982067144,0.0454966796,0.0668525735
C,0,-0.6766363755,-0.3534247895,0.3592828113

H,0,-0.3432712497,-1.4007620624,0.4651957701
C,0,0.4245404813,0.3979982087,-0.4315580516
H,0,0.0888312856,1.4263589013,-0.5926892691
C,0,1.7430782397,0.400230427,0.3638004802
H,0,2.4269840381,1.15809066,-0.0733172327
H,0,1.6081313461,0.6923537228,1.4157541172
O,0,-0.8674617535,0.2418927208,1.6172403597
H,0,-1.8161437836,0.4429772662,1.6719071327
O,0,0.6281372253,-0.2014688106,-1.6954081146
H,0,1.2685092731,-0.913344825,-1.5479162302
O,0,2.4210457716,-0.7798863145,0.2482660024

Threose, loss of H from C4OH
H CBS-QB3 -456.79277
C,0,-0.1846494253,-1.0563208716,-1.7264309211
H,0,0.0317340323,-2.1211472158,-1.5164239754
O,0,-0.7911203958,-0.7108858479,-2.7109825565
C,0,0.3338542077,-0.0474146329,-0.7243977393
C,0,-0.1627419904,-0.34779833,0.6985921399
H,0,0.0058628867,-1.4028056748,0.9353687708
C,0,0.6078454493,0.5065265973,1.7318117249
H,0,0.5097077657,1.5733089116,1.4356169328
H,0,0.1304547619,0.4381237716,2.7238582239
O,0,-1.5534630162,-0.1192181641,0.7982965473
H,0,-1.7146317723,0.7169972595,0.3389792623
O,0,1.9448965015,0.2695636998,1.7788067726
H,0,1.4334893269,-0.1155755544,-0.7200910531
O,0,-0.0979022518,1.2516746686,-1.0800371728
H,0,-0.4933350803,1.1649723831,-1.9629669562

Erythulose, loss of C1-H
H CBS-QB3 -456.83847
C,0,-1.3160565367,1.2970193316,-0.2290909104
H,0,-1.0876686929,1.6443557089,-1.2262496936
C,0,-0.8329096701,0.1080400343,0.3856651058
C,0,0.0876139489,-0.8688758169,-0.3434494531
H,0,-0.460099253,-1.2741151146,-1.2093623836
C,0,1.3877849873,-0.2204835568,-0.8349567486
H,0,1.1821349549,0.624629583,-1.4966237628
H,0,1.9445736972,-0.9728175601,-1.4104698369
O,0,-1.2057859507,-0.15144158,1.5476187348
O,0,-2.1334665739,2.0732188619,0.4731750989
H,0,-2.2313010633,1.6251051898,1.3388780861

O,0,0.4384927275,-1.9106889788,0.5467557309
H,0,-0.1219250753,-1.7807538728,1.3301055905
O,0,2.1445708868,0.2708197577,0.2519987631
H,0,2.2040446134,-0.4640099872,0.8760056789

Erythulose, loss of C1-OH
H CBS-QB3 -381.67488
C,0,-0.9062327765,1.7285431227,-0.6992357587
H,0,-1.126768949,2.0844120331,0.2992168359
H,0,-1.3932576844,2.1925222796,-1.547098023
C,0,0.0234814927,0.6693149055,-0.943811601
C,0,0.7328659505,-0.0195337508,0.2308399643
H,0,1.2995976129,0.7506950524,0.7785551244
C,0,-0.2399777586,-0.7061603724,1.1997440472
H,0,-0.9723916855,0.0014584523,1.595156628
H,0,0.3444438727,-1.096442986,2.0441969672
O,0,0.3054486398,0.3095903131,-2.091752529
O,0,1.6047188935,-1.0079604785,-0.276421782
H,0,1.5767403417,-0.89944123,-1.2426339686
O,0,-0.9584780228,-1.7310847514,0.5441791632
H,0,-0.2901899271,-2.2759105897,0.1090659323

Erythulose, loss of H from C1OH
H CBS-QB3 -456.79225
C,0,0.053861231,-0.4461013178,-1.9838206394
H,0,0.8451288858,-1.0776041625,-1.5273793418
H,0,-0.571170897,-1.0205676456,-2.6788913084
C,0,-0.8532817221,-0.2199070114,-0.6479047647
C,0,-0.2273703996,0.6806659284,0.4344813944
H,0,-0.9020102112,1.5506871922,0.4940119753
C,0,-0.2159560424,-0.0586160305,1.7769786864
H,0,-1.2098521455,-0.4485064099,1.9969899471
H,0,0.0668550575,0.6602031966,2.5565419327
O,0,-1.923440617,-0.7285662095,-0.5730330815
O,0,0.4761503106,0.7357424831,-2.4123346095
O,0,1.0979105688,1.0488194517,0.1580057059
H,0,1.1602076408,1.2777037353,-0.7901063226
O,0,0.672926398,-1.1579232682,1.7264083642
H,0,1.5300429423,-0.7960289319,1.4700520618

Erythulose, loss of C3-H
H CBS-QB3 -456.83551
C,0,0.9567724362,0.0616552473,-1.3355862784

H,0,0.026019957,-0.178179868,-1.864190337
H,0,1.0521067371,1.159230155,-1.3444313792
C,0,0.8376521341,-0.4024095748,0.1049971966
C,0,-0.2379171332,0.0218604216,0.952316391
C,0,-1.3977006515,0.885814107,0.5712982848
H,0,-1.0499542119,1.8166400946,0.1168815569
H,0,-1.9553286776,1.1386186043,1.4796350495
O,0,1.6948063113,-1.1736873323,0.5783326959
O,0,2.0610765443,-0.5358838611,-1.9637627354
H,0,2.5017585995,-1.0567128932,-1.2746649949
O,0,-0.2288599395,-0.4668157586,2.1983042148
H,0,0.5761168446,-1.0252104595,2.242917531
O,0,-2.2312040992,0.27918062,-0.4201127909
H,0,-2.6053438511,-0.5240995022,-0.0419364048

Erythulose, loss of C3-OH
H CBS-QB3 -381.67317
C,0,-0.1923683012,1.4632379976,1.2551025526
H,0,0.3542735608,1.0915580515,2.1349054947
H,0,0.4350639021,2.2434109836,0.7976902
C,0,-0.3381929796,0.320593694,0.2573011953
C,0,0.8325300351,-0.3450334418,-0.2200430172
H,0,1.7925920769,-0.0725273065,0.2096969745
C,0,0.8225899791,-1.4717166669,-1.1914837715
H,0,1.0563995707,-2.3884214011,-0.6072985504
H,0,1.6724579376,-1.3561736899,-1.8787358988
O,0,-1.4863946773,0.0003443224,-0.1000217628
O,0,-1.43837308,1.9801069608,1.6269599094
H,0,-2.0926202853,1.4645721131,1.1289191642
O,0,-0.3377607563,-1.6464493315,-1.9601381395
H,0,-1.0801979827,-1.2835002853,-1.4528533505

Erythulose, loss of H from C3OH
H CBS-QB3 -456.79264
C,0,-1.3974092766,1.4064733812,-0.5016292104
H,0,-0.9913850769,1.648127752,-1.4984113339
H,0,-2.4868510067,1.4410373791,-0.5543154992
C,0,-0.9909788116,-0.0580504456,-0.2824640203
C,0,0.4207018278,-0.2282617528,0.5994581046
H,0,-0.0376481053,-0.1135410267,1.6070839475
C,0,1.0141407457,-1.6207947372,0.3523341959
H,0,0.2316085869,-2.3805925551,0.3501324844
H,0,1.7083259191,-1.8313508226,1.1797808901

O,0,-1.5710899657,-1.011930085,-0.6738925337
O,0,-0.9644365736,2.2749050466,0.4973686321
H,0,0.007242836,2.2094530016,0.5205386655
O,0,1.2244452015,0.761089662,0.2540959288
O,0,1.6617322744,-1.6581409217,-0.8988734617
H,0,2.1716014251,-0.8384248758,-0.95120779

Erythulose, loss of C4-H
H CBS-QB3 -456.81371
C,0,-1.7787013738,0.0003994424,0.0531142494
H,0,-1.7465692544,-1.0672169493,-0.1948957538
H,0,-2.0928949429,0.0727696004,1.1080176191
C,0,-0.3841502633,0.5863747743,-0.0290398815
C,0,0.7739799374,-0.0738027594,0.7286294211
H,0,0.4546836477,-0.2836132846,1.7586416542
C,0,1.1620655265,-1.354626371,0.0336433563
H,0,0.5128141554,-2.2176785346,-0.0194725218
O,0,-0.1774881657,1.5846367208,-0.6967187641
O,0,-2.6775257895,0.6671354397,-0.7907501819
H,0,-2.1958863895,1.4263844947,-1.1509713073
O,0,1.8916060404,0.798261063,0.709300582
H,0,1.5991252288,1.5845064471,0.2188232905
O,0,2.0738066765,-1.2646000558,-0.958907705
H,0,2.5851369662,-0.4589310276,-0.7694130573

Erythrulose, loss of C4-OH
H CBS-QB3 -381.66132
C,0,1.3127667977,0.7807463343,0.2539794711
H,0,0.7189117988,1.6401605847,0.6026251543
H,0,1.6626809608,0.2521014865,1.1501629952
C,0,0.3747528401,-0.1219492119,-0.5207251905
C,0,-0.7756423354,-0.8494412344,0.2087255944
H,0,-0.2805353138,-1.5239969315,0.9304399859
C,0,-1.6390800437,0.1161650221,0.944108431
H,0,-2.5190981284,0.5007939822,0.4468442343
H,0,-1.3926453766,0.4447216507,1.9453091202
O,0,0.5229682916,-0.2772691054,-1.7151684413
O,0,2.4070891371,1.1907330309,-0.5211371794
H,0,2.1958681798,0.9519129972,-1.435287502
O,0,-1.523850009,-1.5960874976,-0.7201990944
H,0,-1.064185799,-1.5085901077,-1.569677579

Erythrulose, loss of H from C4OH

H CBS-QB3 -456.79501
C,0,-1.1660716369,-1.2143968523,-0.1112023643
H,0,-1.3753134304,-0.7675380745,-1.0911318542
H,0,-0.5974535664,-2.1372456378,-0.3134890612
C,0,-0.2499622581,-0.299685671,0.6682175189
C,0,1.0402323686,0.2154198364,0.0415815445
H,0,1.5876877801,-0.6355777258,-0.3846229299
C,0,0.7409247194,1.2104623812,-1.1114932419
H,0,1.7089226109,1.6351639622,-1.4451460911
H,0,0.1998405049,2.0953615761,-0.7243902173
O,0,-0.5283505829,0.024219252,1.8081560704
O,0,-2.3561760313,-1.4820429864,0.5792610605
H,0,-2.2327703268,-1.1394673044,1.4763330401
O,0,1.8275450119,0.8690303449,1.0045725779
H,0,1.2854153953,0.9151269391,1.8079840675
O,0,0.1155294418,0.7111709603,-2.20463112

α-Erythrofuranose, loss of C1-H
H CBS-QB3 -456.81557
O,0,0.2414294287,1.2370011675,-1.0221937094
C,0,-0.9622629006,0.6170830267,-0.7537166007
C,0,-0.7606043674,-0.5819431269,0.1209772789
C,0,0.689151774,-0.9659088402,-0.2281111222
C,0,1.3179070735,0.4144839122,-0.5163620683
H,0,-1.4810188477,-1.3830247457,-0.0631061467
H,0,0.7000477602,-1.5816703868,-1.1302329017
H,0,2.1140857671,0.3893043183,-1.2599706293
H,0,1.6951094291,0.8529441822,0.4122248739
O,0,-1.9652833737,1.4718209369,-0.3670920674
H,0,-1.7896664984,2.3336812613,-0.7638518949
O,0,-0.7590806531,-0.2407822455,1.5217802065
H,0,-1.4291220969,0.4400871613,1.6497616869
O,0,1.34669749,-1.6894496247,0.7810813188
H,0,1.0426090152,-1.3136269967,1.6188127755

α-Erythrofuranose, loss of C1-OH
H CBS-QB3 -381.66197
O,0,0.3317290837,0.3118099819,-1.7235263386
C,0,0.1587267444,1.3096313953,-0.809354725
C,0,-0.3051288584,0.8130305393,0.5068811003
C,0,-0.6764925067,-0.6603148582,0.1814531158
C,0,0.229503795,-0.9504775214,-1.0295476097
H,0,0.0841965979,2.3052561769,-1.2198979213

H,0,-1.1402046197,1.3720703794,0.9330371194
H,0,-1.7257142675,-0.7124260643,-0.1230364093
H,0,-0.1678437484,-1.6826092208,-1.7302976417
H,0,1.2225013514,-1.2678786453,-0.6925194594
O,0,0.725214395,0.8111254973,1.536419735
H,0,1.5625650669,0.9989198546,1.0962088221
O,0,-0.4884512843,-1.5357706154,1.26205478
H,0,0.1893992509,-1.1123668994,1.8121254324

α-Erythrofuranose, loss of H from C1OH, BDE 90.8 kcal/mol
H CBS-QB3 -456.82395
O,0,0.0892410914,0.7694028402,-1.5788702148
C,0,0.9845893204,-0.1507569949,-1.9242126553
C,0,0.4680253254,0.7704377015,1.2940785818
C,0,-0.6572509673,-0.0301090715,0.7065838481
C,0,-1.0243427301,0.3802561278,-0.7231384072
H,0,1.7059145442,0.2904428606,-2.6233365825
H,0,0.4934851522,1.8526220515,1.2454773251
H,0,-1.5828373063,0.108090758,1.2922925275
H,0,-1.6431535422,1.2763738213,-0.7101796621
H,0,-1.5732265114,-0.4336996434,-1.2016078039
O,0,1.022091259,-1.2976683411,-1.551238771
O,0,1.0329222787,0.2548103846,2.4181710626
H,0,0.8443936924,-0.6985365454,2.3866286547
O,0,-0.3726387535,-1.4246522642,0.8535197266
H,0,0.2127861471,-1.6670146849,0.1158333703

α-Erythrofuranose, loss of H from C1OH, BDE 106.9 kcal/mol
H CBS-QB3 -456.79837
O,0,0.4058257356,0.0739542753,-1.7049965075
C,0,0.711554058,0.9670674333,-0.6638039004
C,0,-0.4288702433,0.7575036913,0.4028801316
C,0,-0.5858760756,-0.7737130869,0.3010266848
C,0,-0.3446305921,-1.044892571,-1.1836800162
H,0,0.662938457,2.0020015756,-1.0553378201
H,0,-1.3397661106,1.2638627553,0.0739870055
H,0,-1.5810986735,-1.0953694633,0.6321931386
H,0,-1.2820531657,-1.1142397542,-1.741676936
H,0,0.2257785219,-1.9666882767,-1.3126983118
O,0,1.9091878323,0.8322215902,-0.0883963968
O,0,-0.0765351709,1.2181433549,1.6793561645
H,0,0.8872820167,1.3138427432,1.6740110775
O,0,0.4317249085,-1.4087815668,1.0504540814

H,0,0.4045385018,-1.0249117002,1.936680605

α-Erythrofuranose, loss of C2-H
H CBS-QB3 -456.81402
O,0,0.1159736922,-0.2861001442,-1.6270705655
C,0,0.440293936,-1.1346709663,-0.5495921027
C,0,0.719707775,-0.2110414512,0.6083174203
C,0,0.0683419826,1.1013387056,0.3104903567
C,0,0.1140250802,1.0966098084,-1.2116603789
H,0,1.2728039763,-1.7875811333,-0.8287461062
H,0,0.5656304555,1.9732991032,0.7480623661
H,0,1.0248774094,1.583507555,-1.5743652905
H,0,-0.7600553594,1.5878321128,-1.6401512273
O,0,-0.6177027702,-2.0340140587,-0.2356459762
H,0,-1.400875962,-1.4828412852,-0.0952594603
O,0,0.6370013708,-0.6898929898,1.8814879224
H,0,0.4557848903,-1.6404376354,1.8288246881
O,0,-1.3184526515,1.0627343505,0.721089959
H,0,-1.3173558253,0.8612590287,1.6642193951

α-Erythrofuranose, loss of C2-OH
H CBS-QB3 -381.66086
O,0,0.4377328303,0.1215159413,-1.3849315559
C,0,0.085921153,1.1651150774,-0.503171289
C,0,-0.9925551678,0.6020534903,0.356814153
C,0,-0.6757824462,-0.8491757549,0.5080552628
C,0,0.0401545177,-1.1519620344,-0.8217347847
H,0,-0.1867912162,2.0340138441,-1.1098555436
H,0,-1.5887667582,1.1809206547,1.0478284389
H,0,-1.5428416176,-1.4916355793,0.6661874194
H,0,-0.6221144158,-1.6400140406,-1.5421150777
H,0,0.9192795829,-1.777724453,-0.6541227793
O,0,1.1826852185,1.5001821301,0.3630474303
H,0,1.932974624,1.7225585323,-0.2023376549
O,0,0.1671840745,-1.0539963455,1.6557691532
H,0,0.8429196209,-0.3618524624,1.6205678276

α-Erythrofuranose, loss of H from C2OH
H CBS-QB3 -456.79967
O,0,-0.0552476867,0.1147627124,-1.6302516636
C,0,0.3315670561,1.1172845123,-0.7462135321
C,0,-0.6893146372,0.7768040774,0.5510122012
C,0,-0.4416436064,-0.7620426118,0.5516665755

C,0,-0.3091900848,-1.1204425032,-0.9274496865
H,0,0.0825938546,2.0868848033,-1.1699540226
H,0,-1.6836552292,1.0524753393,0.161926008
H,0,-1.2597629957,-1.2957866523,1.0470278266
H,0,-1.2282686684,-1.5391555863,-1.3394767238
H,0,0.5104696711,-1.8280455319,-1.0801879175
O,0,1.6498059598,1.1194800146,-0.3833112077
H,0,1.8321796118,0.3023580953,0.1111227774
O,0,-0.3185453262,1.3885994872,1.6483522861
O,0,0.7922930717,-1.0072851305,1.2211679337
H,0,0.7867180094,-0.4058910259,1.9845701454

α-Erythrofuranose, loss of C3-H
H CBS-QB3 -456.81731
O,0,-0.0476152554,-0.2121845655,-1.6581608869
C,0,-1.0153025912,0.0408428535,-0.6749100145
C,0,-0.5169642509,-0.6699544172,0.6046263756
C,0,0.9846687677,-0.6494968874,0.3808659829
C,0,1.2616481376,-0.247431559,-1.0351075008
H,0,-1.9677444039,-0.3235993031,-1.0675180414
H,0,-0.9203918294,-1.6864646568,0.6476467699
H,0,1.8593242518,-0.9557292574,-1.6137540173
H,0,1.7448954289,0.7389671219,-1.0752402812
O,0,-1.1030002156,1.4167588047,-0.3341468642
H,0,-1.3610931746,1.9112525924,-1.118481308
O,0,-0.8795392318,-0.0293388741,1.8194646307
H,0,-1.003506512,0.9049216562,1.5960189113
O,0,1.7694711849,-0.1535657943,1.3732911323
H,0,1.1951486939,-0.0849777137,2.1554051117

α-Erythrofuranose, loss of C3-OH
H CBS-QB3 -381.66526
O,0,-0.3305415856,0.0231483717,-1.4665048866
C,0,-0.5792823982,-0.809765606,-0.3623128495
C,0,0.6352653648,-0.6389132399,0.5867619611
C,0,1.0492386777,0.7605548534,0.2508534821
C,0,0.3855736767,1.1933250309,-1.0053388618
H,0,-0.7165319127,-1.8219679858,-0.7504714665
H,0,1.4125851907,-1.3634591288,0.3096981679
H,0,1.7371259288,1.3510890812,0.8383479215
H,0,1.079134033,1.4857770441,-1.8027305938
H,0,-0.316662439,2.0281218364,-0.8554002107
O,0,-1.7127637955,-0.3933626273,0.3808014581

H,0,-2.4842543844,-0.4492248146,-0.1917823056
O,0,0.3463131949,-0.8934543681,1.9473582811
H,0,-0.5052005512,-0.4718694472,2.1207199028

α-Erythrofuranose, loss of H from C3OH
H CBS-QB3 -456.80020
O,0,-0.0593082002,0.2191852525,-1.5823733246
C,0,1.0036589316,0.2097223634,-0.6643124569
C,0,0.4061963932,0.6148009724,0.6950309077
C,0,-1.0317258357,-0.2229687603,0.5620854253
C,0,-1.3245144484,0.1796309396,-0.8966092184
H,0,1.7763217107,0.9061812261,-0.9964185986
H,0,0.1036781311,1.6636503579,0.7379376623
H,0,-1.7159702112,0.2074402089,1.3103398436
H,0,-1.8026487849,1.1661103055,-0.935221756
H,0,-1.9639593291,-0.5526080386,-1.3909158773
O,0,1.6246544382,-1.0449830043,-0.5542889805
H,0,0.9066964332,-1.6835900611,-0.3940030549
O,0,1.1842960931,0.3366875453,1.7832902147
H,0,1.6476681351,-0.4936089971,1.5914624558
O,0,-0.7550434567,-1.5056503103,0.7339957579

α-Erythrofuranose, loss of C4-H
H CBS-QB3 -456.81914
O,0,0.4004485648,-0.0899879128,-1.761503481
C,0,0.2603669852,0.9802469516,-0.818654847
C,0,-0.8112962031,0.4765732426,0.1749883572
C,0,-0.4707743027,-1.0336849478,0.2187081119
C,0,-0.0571472893,-1.2515438898,-1.1828614867
H,0,-0.0016919883,1.8800430142,-1.3711518638
H,0,-1.8121828068,0.6215054248,-0.2394473078
H,0,-1.3090089621,-1.6629596476,0.5315599565
H,0,0.2289065895,-2.1675790366,-1.67490497
O,0,1.4675582592,1.2470599625,-0.1567637547
H,0,1.7224888903,0.4215885149,0.2905808974
O,0,-0.7352003389,1.1070131004,1.4356801622
H,0,0.1138289873,1.5710205245,1.4549738478
O,0,0.6489368582,-1.2289941338,1.1244221405
H,0,0.3547667567,-0.8703021672,1.9743732374
